# Myeloid-specific S100A8/A9 deficiency attenuates atrial fibrillation through prevention of TLR4/NF-kB-mediated immune cell recruitment and inflammation

**DOI:** 10.3389/fimmu.2025.1623486

**Published:** 2025-09-04

**Authors:** Qing Wang, Hua Shen, Jing Wang, Guifang Wang, Yufeng Zhang

**Affiliations:** ^1^ Department of Thoracic Surgery, Shanghai Chest Hospital, Shanghai Jiaotong University School of Medicine, Shanghai, China; ^2^ Department of Cardiovascular Surgery, Chinese PLA General Hospital, Beijing, China; ^3^ Department of Cardiothoracic Surgery, Shuguang Hospital Affiliated to Shanghai University of Traditional Chinese Medicine, Shanghai, China; ^4^ Department of Cardiology, Xinxiang City No.1 People’s Hospital, Xinxiang, Henan, China; ^5^ Department of Cardiothoracic Surgery, Changzheng Hospital, Naval Medical University, Shanghai, China

**Keywords:** atrial fibrillation, S100A8/A9, monocytes, inflammation, TLR4/NF-κB signaling, conditional knockout

## Abstract

**Background:**

Atrial fibrillation (AF) is the most common sustained arrhythmia, frequently associated with inflammation and atrial remodeling. S100A8/A9, a calcium-binding protein complex enriched in myeloid cells, has been implicated in cardiovascular inflammation, yet its role in AF remains unexplored. This study aims to investigate the mechanistic contribution of myeloid-derived S100A8/A9 to AF pathogenesis and assess its therapeutic potential through targeted genetic deletion.

**Methods:**

Transcriptomic and single-cell RNA sequencing data from AF patients were analyzed to identify differentially expressed genes (DEGs) and immune cell signatures. S100A8/A9 emerged as top hub genes. Monocyte/macrophage-specific S100A9 conditional knockout (CKO) mice were subjected to Ach-CaCl_2_–induced AF, with assessments of electrophysiology, fibrosis, inflammation, and TLR4/NF-κB signaling. The functional role of this pathway was further tested using the NF-κB activator HY-18739.

**Results:**

S100A8/A9 expression was significantly elevated in atrial tissues and myeloid cell clusters of AF patients. S100A9 CKO mice exhibited reduced AF inducibility and duration, accompanied by attenuation of atrial fibrosis, inflammatory cytokine production, and monocyte infiltration. Mechanistically, S100A9 deletion suppressed activation of the TLR4/IRAK1/TRAF6/NF-κB pathway. These effects were reversed by pharmacologic NF-κB reactivation with HY-18739, confirming the centrality of this pathway.

**Conclusion:**

Myeloid-derived S100A8/A9 amplifies AF by promoting monocyte recruitment and inflammation via the TLR4/NF-κB axis. Targeting this pathway may offer a promising therapeutic strategy for AF prevention and treatment.

## 1 Introduction

Atrial fibrillation (AF) is a prevalent cardiac arrhythmia associated with substantial health and socioeconomic implications (1). Beyond compromising patient quality of life, AF correlates with an increased risk of stroke, incident heart failure, dementia, and heightened mortality (2). Identifying the precise mechanisms underlying AF in individual patients remains challenging, and the efficacy of current therapeutic interventions is suboptimal.

Inflammation is increasingly recognized as a key factor in the pathogenesis of AF (3). Chronic inflammation has been shown to disrupt the atrial myocardium, leading to electrical remodeling and structural changes that promote AF initiation and maintenance (4). Pro-inflammatory cytokines, such as interleukin-6 (IL-6), tumor necrosis factor-alpha (TNF-α), and C-reactive protein (CRP), are elevated in AF patients and contribute to both atrial fibrosis and electrophysiological disturbances (5). Furthermore, inflammatory cells, including monocytes and macrophages, infiltrate the atrial tissue, where they release pro-inflammatory mediators that exacerbate the arrhythmogenic substrate (6, 7). These findings highlight the intricate relationship between inflammation and AF, suggesting that targeting inflammatory pathways could be a promising strategy for managing AF.

S100 proteins, encompassing S100A8 and S100A9, represent the predominant subgroup within the Ca^2+^-binding EF-hand superfamily (8). Primarily localized in the cytoplasm of neutrophils and monocytes, S100A8/A9 belongs to a vast family of Ca^2+^-binding proteins and is integral to the inflammatory response (9, 10). Recent investigations have unveiled a direct association between S100A8/A9, inflammation onset, and cardiac damage post-myocardial infarction (11). Furthermore, elevated serum levels of S100A8/A9 correlate with major adverse cardiac events in myocardial infarction patients. This implicates that strategies targeting neutrophils or their inflammatory constituents might attenuate heart failure risks (12). Yet, there is a lack of clinical and experimental research examining the role of S100A8/A9 in AF.

In this study, we employed bioinformatics methodologies to analyze three GEO datasets, aiming to identify differentially expressed genes (DEGs) in AF patients relative to matched controls. Preliminary analysis suggested a pivotal role for S100A8/A9 in AF-related pathological processes. Consequently, our research also investigated S100A8/A9 expression patterns in both AF patients and murine models, seeking to clarify its function and the mechanisms contributing to AF development. ​This study aims to identify a new therapeutic target for enhancing AF treatment modalities. The study’s schematic representation is depicted in **Supplementary Figure S1**.

## 2 Materials and methods

### 2.1 Microarray data retrieval

We obtained microarray gene expression datasets related to AF and control groups with sinus rhythm (SR) from the GEO Datasets. Using the search term “atrial fibrillation,” and applying filters for “Homo sapiens” and “Expression profiling by array,” we selected datasets based on three criteria: 1. Tissue samples from the atria or appendage from AF patients and SR controls; 2. mRNA gene expression profiling; 3. A minimum of three samples per group. Following this criteria, we selected three datasets: GSE41177 (13), GSE79768 (14), and GSE115574 (15). Combined, these datasets comprise data from 38 AF patients and 24 SR controls. **Supplementary File 1** provides detailed information on these datasets.

### 2.2 Identification of DEGs

We accessed the data using the R package “GEO query”. DEGs were identified with the “limma” package. The Benjamini-Hochberg method adjusted p-values, with significance set at adjusted p < 0.05 and an absolute log2-fold change ≥ 0.5. Subsequent analysis used the Robust Rank Aggregation (RRA) method to rank the DEGs. Visualization of DEGs was achieved with Volcano Plots [using “ggplot2” (16)] and Heatmaps [using “ComplexHeatmap” (17)]. We also employed a Venn Diagram to determine overlapping DEGs (18).

### 2.3 Functional enrichment analysis

We conducted Gene Ontology (GO) and Kyoto Encyclopedia of Genes and Genomes (KEGG) pathway enrichment analyses on DEGs with the “ClusterProfiler” package (19). Statistically significant items had p-values less than 0.05 following the Benjamini-Hochberg test.

### 2.4 Protein-Protein Interaction network and hub genes identification

To build the PPI network of DEGs, we utilized the STRING database (V12.0) to predict protein associations (20). The network visualization was performed using Cytoscape software (V3.10.1) (21). After network construction, we assessed each node’s “degree” using the CytoHubba and MCODE plugins. Nodes with high connectivity indicated their relevance to AF, designating them as hub genes for further *in vivo* studies.

### 2.5 Immune infiltration analysis

We combined gene matrices from GSE41177, GSE79768, and GSE115574 using a Perl script, followed by normalization. The ImmuCellAI tool analyzed the resultant normalized gene expression matrix for immune infiltration, employing the Wilcoxon rank-sum test for group comparisons (22).

### 2.6 Singe-cell analysis

Public sc-RNA sequencing data of GSE224959 was utilized to validate the key DEGs found in previous microarray analysis (23). Seurat 4.0 conducted post-processing, discarding cells with under 250 genes or 500 UMIs and over 25% mitochondrial UMIs. Genes found in less than 10 cells were excluded. Normalization and integration of data across samples were performed to reveal common cell states, with principal component analysis clustering the cells based on the top 40 components. Marker identification for clusters utilized logistic regression to pinpoint DEGs. **Supplementary File 1** provides detailed information on GSE224959 dataset.

### 2.7 Human sample acquisition

We validated S100A8/A9 expression by collecting atrial appendage samples from 7 AF individuals and 7 matched controls who underwent open-heart surgeries at the Chinese PLA General Hospital. Ethical standards ensured anonymous processing, with protocol approval from the hospital’s Ethics Committee. Patient consent was documented, and **Supplementary File 2** provides patient details. We used immunohistochemical staining and RT-PCR to assess S100A8/A9 protein and mRNA expression, respectively.

### 2.8 Animal model

Wild Type (WT) male C57BL/6J mice were sourced from the Experimental Animal Center of the Chinese PLA General Hospital. Housed in an environment free from specific pathogens, our mice were kept at a stable temperature ranging between 20 °C and 25 °C, with relative humidity set between 50% and 60%, and subjected to a regular diurnal cycle.

This study utilized myeloid-specific S100A9 conditional knockout (CKO) mice. The S100A9^fl/fl^mice (Caygen stock #S-CKO-04902) carrying loxP sites flanking exons 1 and 2 were commercially obtained and crossed with Cx3cr1-Cre mice (Caygen stock #C001032) to generate monocyte/macrophage-targeted knockouts. Littermate controls (S100A9^fl/fl^ Cx3cr1^Cre−/−^) and knockouts (S100A9^fl/fl^Cx3cr1^Cre+/−^) were maintained on a C57BL/6 background and genotyped. The S100A9 CKO mice developed without morphological or histological abnormalities in the lungs. Despite normal S100A8 mRNA levels in mature phagocytes, the absence of S100A9 protein prevented formation of functional S100A8/A9 complexes (24, 25).

The study consisted of the following treatment groups: control group (S100A9 WT mice; n = 11), AF model group (S100A9 WT mice +Ach-CaCl_2_; n = 12), S100A9 KO group (S100A9 KO mice+Saline; n = 10), and S100A9 KO + AF model group(S100A9 KO mice+ Ach-CaCl_2_; n = 13). AF was induced in these mice by administering a mixture of Ach (66 μg/kg) and CaCl_2_ (10 mg/kg, Sigma-Aldrich, St Louis, MO) daily through tail vein injection (i.v.) over a period of 4 weeks, as previously described (26).

### 2.9 Transthoracic echocardiography overview

Using the Vevo 1100 imaging device (VisualSonics, Toronto, Canada), we conducted transthoracic echocardiography to assess various cardiac features of the mice. Left atrium diameter (LAD), left ventricle ejection fraction (LVEF), left ventricular fractional shortening (LVFS), left ventricular end-systolic diameter (LVESD), and left ventricular end-diastolic diameter (LVEDD) were measured by a technician who was blind to the grouping, with every parameter measured three times to calculate the average values.

### 2.10 Atrial pacing and AF induction

Mice were anesthetized with 2% isoflurane in 0.5 L/min oxygen and maintained with 1.5% isoflurane. Atrial pacing was performed in 12-week-old mice as per Schrickel, Bielik et al., 2002 (27). Detailed procedures involved anesthesia, electrocardiogram (ECG) recordings, catheter placement, and AF induction assessments were described in our previous study (28). LabChart 8 (version 8.1.13) provided measurements of electrical parameters.

### 2.11 Histology

Tissues were fixed in 4% PFA, embedded in paraffin, sectioned (5 μm), and stained with H&E or Masson’s trichrome. IHC used anti-S100A8 (1:200, Abcam, ab92331), anti-S100A9 (1:200, CST, #73425), CD11b (1:200, BioLegend, #480109), and Ly6C (1:200, BioLegend) with DAB visualization.

### 2.12 Western blotting

Atria-derived protein lysates were subjected to SDS-PAGE and transferred to PVDF membranes. After incubation with primary and secondary antibodies, protein bands were visualized and analyzed using ImageJ, normalized to GAPDH levels.

### 2.13 Quantitative Real-Time PCR

RNA was extracted using TRIzol reagent and reverse-transcribed into cDNA. Quantitative PCR involved SYBR Green Master Mix on QuantStudio^®^ 3 system. Gene expression was normalized to GAPDH levels and computed with the 2^^-ΔΔCT^ method. The PCR primers are listed in **Supplementary File 3**.

### 2.14 HY-18739 preparation and administration

Phorbol 12-myristate 13-acetate (PMA, catalog number HY-18739) was purchased from MedChemExpress (MCE). For *in vivo* experiments, PMA was dissolved as following: a stock solution of 25 mg/mL was prepared in 10% DMSO and further diluted with 40% PEG300, 5% Tween-80, and 45% saline to achieve a final working concentration of 2.5 mg/mL. The solution was prepared fresh daily and administered via intraperitoneal injection at a dose of 0.1 mg/kg in animal models to induce inflammatory activation (29–32).

### 2.15 Statistical analysis

All analyses used GraphPad Prism (Version 9.5.1, Graphpad Software, LLC) except single-cell data (Seurat 4.0 in R). The data is expressed as mean ± standard error of the mean (SEM). For normally distributed variables, comparisons were made using Student’s t-test or one-way analysis of variance (ANOVA), followed by Bonferroni correction for multiple comparisons when applicable. For variables not normally distributed, Kruskal-Wallis test was applied. The chi-square test was utilized for the analysis of count data. Statistical significance was defined as a p-value less than 0.05.

### 2.16 Ethical approval and compliance statement

To ensure humane endpoints for all animals involved in this study, euthanasia was carried out by cervical dislocation. This method was selected for its effectiveness and rapidity, minimizing potential suffering in accordance with ethical guidelines for the humane treatment of laboratory animals. Cervical dislocation was performed by trained personnel with demonstrated proficiency in the technique to ensure immediate loss of consciousness and death without causing distress to the animals.

This study was carried out in strict accordance with the recommendations and approval of the Chinese PLA General Hospital’s Institutional Ethics Committee. We confirm that all experiments and procedures performed in this research involving animals were in compliance with the ethical standards of the institution at which the studies were conducted, and adhere to the guidelines established by Directive 2010/63/EU of the European Parliament and of the Council of 22 September 2010 on the protection of animals used for scientific purposes, as well as the NIH Guide for the Care and Use of Laboratory Animals. The welfare of all animals used in this study was our foremost consideration, and every effort was made to minimize suffering.

## 3 Results

### 3.1 S100A8/A9 identified as the hub genes related to AF

We identified a total of 4847 DEGs from 3 gene expression profiles in left atria of patients with valvular atrial fibrillation [GSE41177 (**Supplementary File 4A**), GSE79768 (**Supplementary File 4B**) and GSE115574 (**Supplementary File 4C**)]. Heatmap plot (**Supplementary Figures S2A–C**) and Volcano plot (**Supplementary Figures S2D–F**) and were used to visualize the DEGs of three dataset. Venn Diagram showed that there were common up-regulated genes and down-regulated genes among three datasets (**Supplementary Figures S2G, H**).

**Figure 1:**
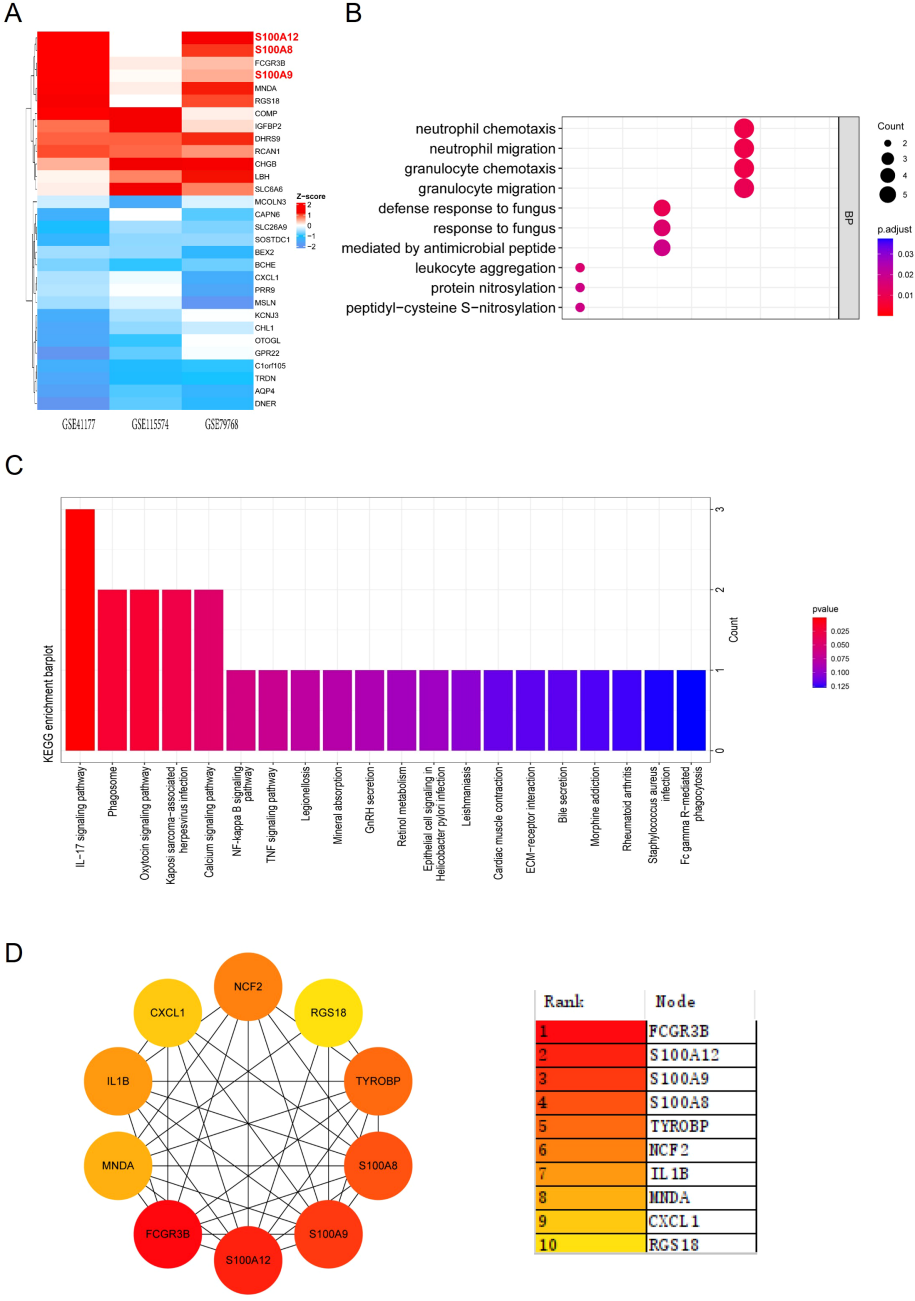
GO and KEGG enrichment analysis and PPI network of DEGs. **(A)** Heatmap showing the top 30 DEGs identified from RRA analysis across the datasets GSE41177, GSE115574, and GSE79768. Red indicates upregulation, while blue indicates downregulation. **(B)** Dot plot illustrating GO enrichment analysis of biological processes related to the top 30 DEGs, with significant terms including neutrophil migration and granulocyte chemotaxis. **(C)** Bar plot depicting KEGG enrichment analysis, highlighting pathways such as the IL-17 signaling pathway and NF-κB signaling pathway. **(D)** PPI network constructed for the top 30 DEGs. The color gradient ranks the hub genes by their significance, with S100A8, S100A9, and S100A12 among the most central nodes.

We then applied the RRA analysis (**Supplementary File 5**) and choose the Top30 DEGs for further investigation (**Figure 1A**). GO function enrichment analysis revealed significant enrichment in biological processes related to inflammation and immunity, including chemotaxis and migration of neutrophils and granulocytes (**Supplementary File 6**, **Figure 1B**). Simultaneously, the KEGG pathway analysis revealed the enrichment of DEGs in immunological regulation pathways, such as “IL-17 signaling pathway”, “phagosome”, “NF-kappa B signaling pathway”, and “chemokine signaling pathway” (**Supplementary File 6**, **Figure 1C**).

The PPI analysis of the Top30 DEGs was conducted using the MCC (Maximal Clique Centrality) algorithm from the CytoHubba plugin, we identified 10 candidate hub genes within the PPI network. These hub genes were S100A8, IL1B, CXCL1, RGS18, MNDA, TYROBP, FCGR3B, S100A9, NCF2, S100A12 (**Figure 1D**). Among these genes, S100 family (S100A8, S100A9, and S100A12) were the most significantly upregulated genes in the atrium tissues of AF patients compared with SR patients.

### 3.2 Single-cell analysis revealed increased monocyte infiltration in AF patients

**Figure 2:**
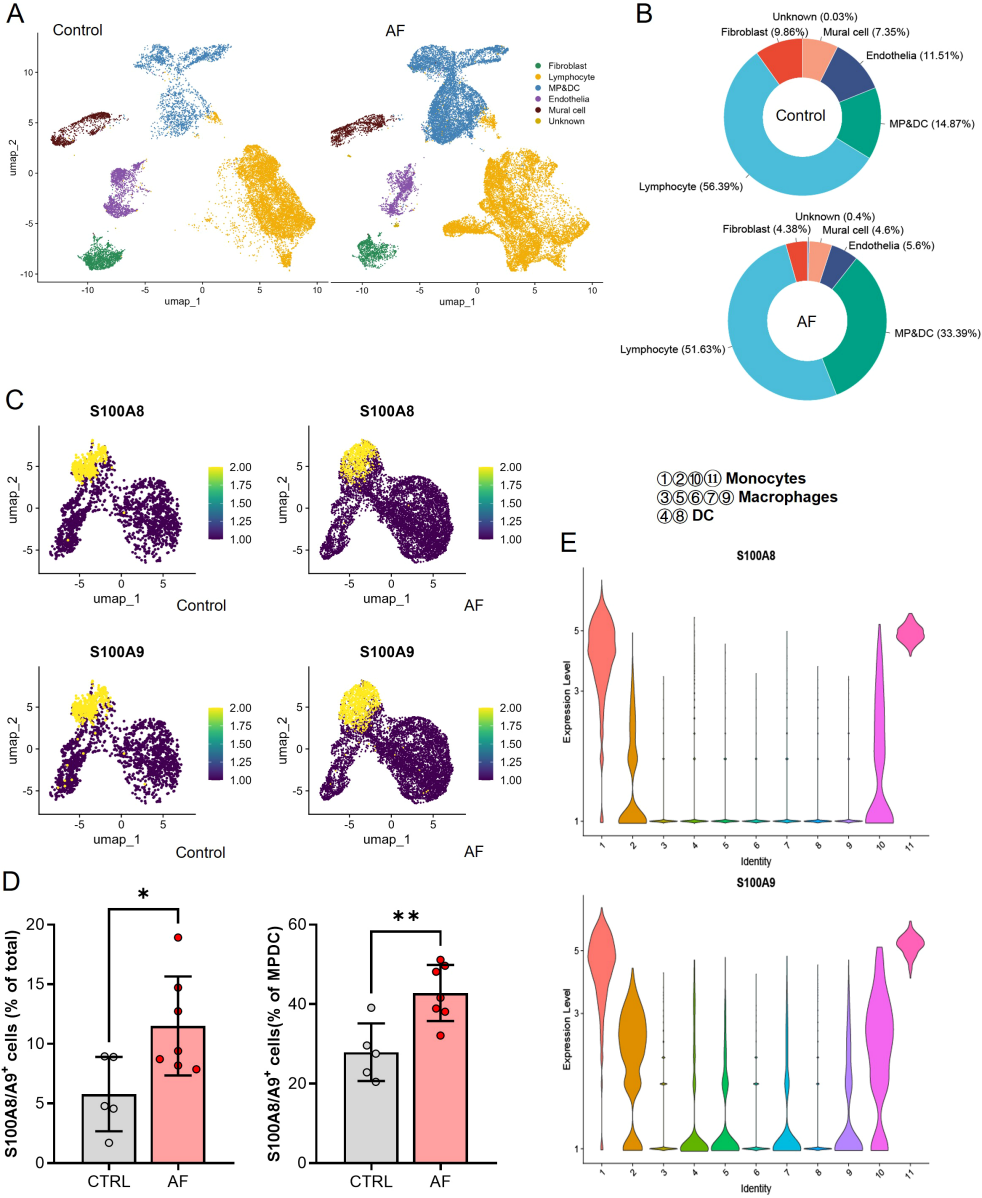
Single-cell RNA sequencing analysis of non-cardiomyocyte cell types in atrial tissues from control and AF patients. **(A)** UMAP visualization showing the clustering of six major non-cardiomyocyte cell types, including lymphocytes, fibroblasts, mural cells, endothelia, and mononuclear phagocyte/dendritic cells (MP/DCs), in control and AF samples. **(B)** Proportional distribution of the six non-cardiomyocyte cell types in control and AF samples, highlighting an increased proportion of MP/DCs in AF. **(C)** UMAP plots showing the expression levels of S100A8 and S100A9 in the identified clusters, with elevated expression observed in the MP/DC population of AF samples. **(D)** Bar plots quantifying the percentage of S100A8/A9+ cells in total cells and within the MP/DC cluster, demonstrating a significant increase in AF samples compared to controls (*P < 0.05, **P < 0.01). **(E)** Violin plots of S100A8 and S100A9 expression levels across different MP/DC subsets, revealing higher expression in monocytes and macrophages in AF samples.

In our single-cell analysis of non-cardiomyocyte cell types in human atrial tissue, as illustrated in **Figure 2** from the dataset GSE224959, we identified six primary cell types using UMAP in both AF and control patient samples (**Figure 2A**). We observed that the cell proportions of mononuclear phagocytes and dendritic cells (MP/DCs) clusters were higher in AF patients compared with Control (33.39% vs 14.87%, **Figure 2B**). Upon examining the expression of S100A8/A9, we discovered a significant upregulation in AF patients compared to the control group across all non-cardiomyocyte cell types, particularly within the MP/DCs (**Supplementary Figure S3**, **Figures 2C, D**). The violin plots underscored this finding, showcasing elevated S100A8/A9 gene expression levels in distinct MP/DC subsets, with S100A8 expressed in monocytes and S100A9 mostly monocytes and macrophages, suggesting that these proteins could be implicated in the heightened inflammatory response observed in AF (**Figure 2E**). This is also supported by the analysis of the infiltration of 36 different immune cell types using the ImmuCellAI algorithm. This analysis involved a comparison between the AF and SR patients in the GSE41177, GSE79768, and GSE115574 datasets. The heatmap visualized the immune cell abundance between AF and SR patients (**Supplementary Figure S4A**). The most abundant immune cells in atrium of AF and SR patients were DC, B cell, Monocyte, Macrophages, NK, Neutrophil, CD4_T, CD8_T, NKT, and Tgd (**Supplementary Figure S4B**).We further analyzed the relationship between immune cell abundance and the expression level of hub genes (**Supplementary Figure S4C**) and found that the monocyte abundance was significantly associated with S100A8/A9 expression. These data indicated that S100A8/A9 might play a key role in the development of AF via inflammation and infiltration of immune cells, especially the monocytes.

### 3.3 S100A8/A9 expression is elevated in AF patients

**Figure 3:**
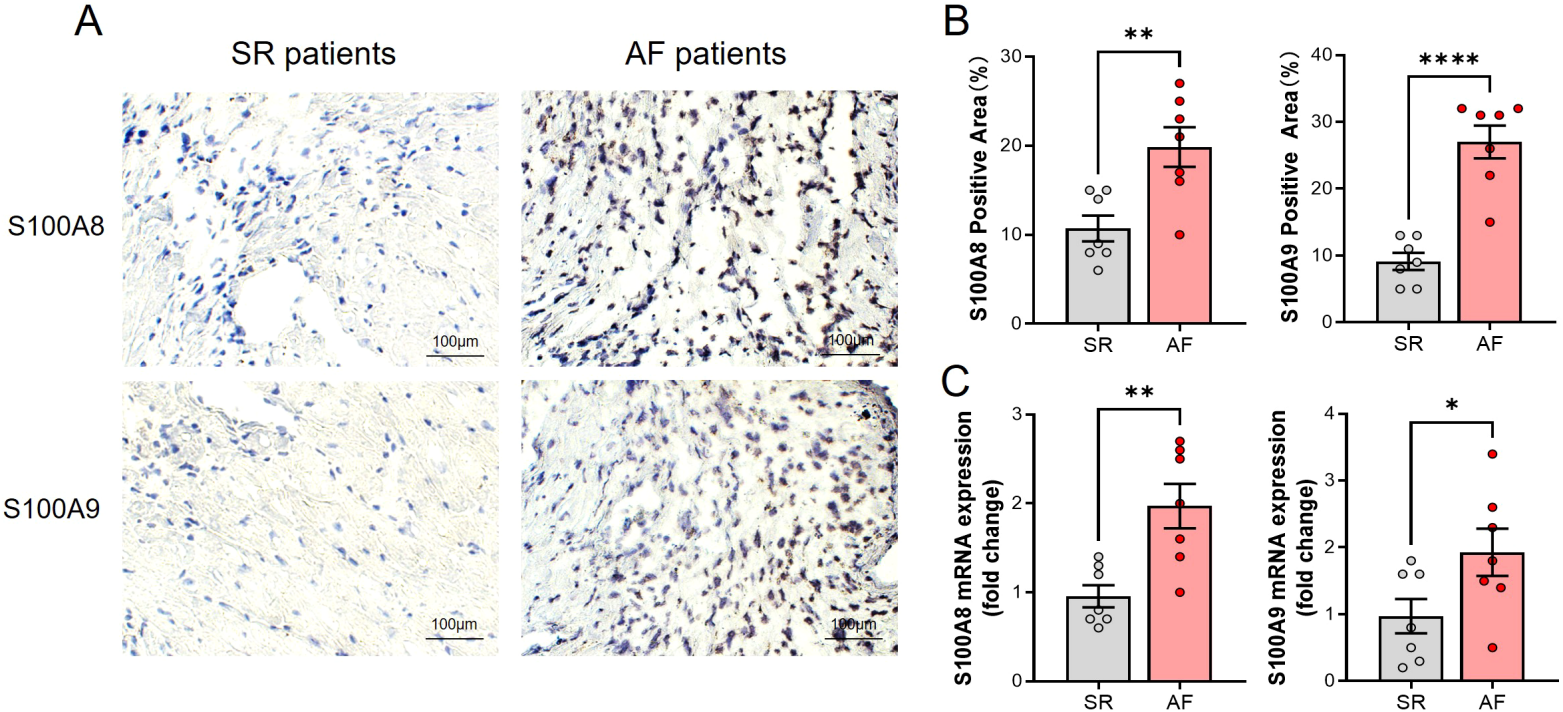
Elevated S100A8/A9 expression and increased monocyte infiltration in atrial tissues of AF patients. **(A)** Representative immunohistochemical staining of S100A8 and S100A9 in atrial tissues from SR and AF patients, showing increased staining intensity in AF patients. **(B)** Quantification of S100A8- and S100A9-positive areas (%), demonstrating a significant increase in AF samples (**P < 0.01, ****P < 0.0001). **(C)** Relative mRNA expression levels of S100A8 and S100A9 in atrial tissues, showing significant upregulation in AF patients compared to SR patients (*P < 0.05, **P < 0.01).

Consistent with the result of bioinformatic analysis, the S100A8/A9 level in the atrium tissues of AF patients was significantly higher than the SR patients as evidenced by immunohistochemistry (IHC) (**Figures 3A, B**). Meanwhile, the S100A8/A9 mRNA level was significantly increased in AF patients compared with the SR patients (**Figure 3C**).

### 3.4 S100A9 CKO reduces AF inducibility and duration

**Figure 4:**
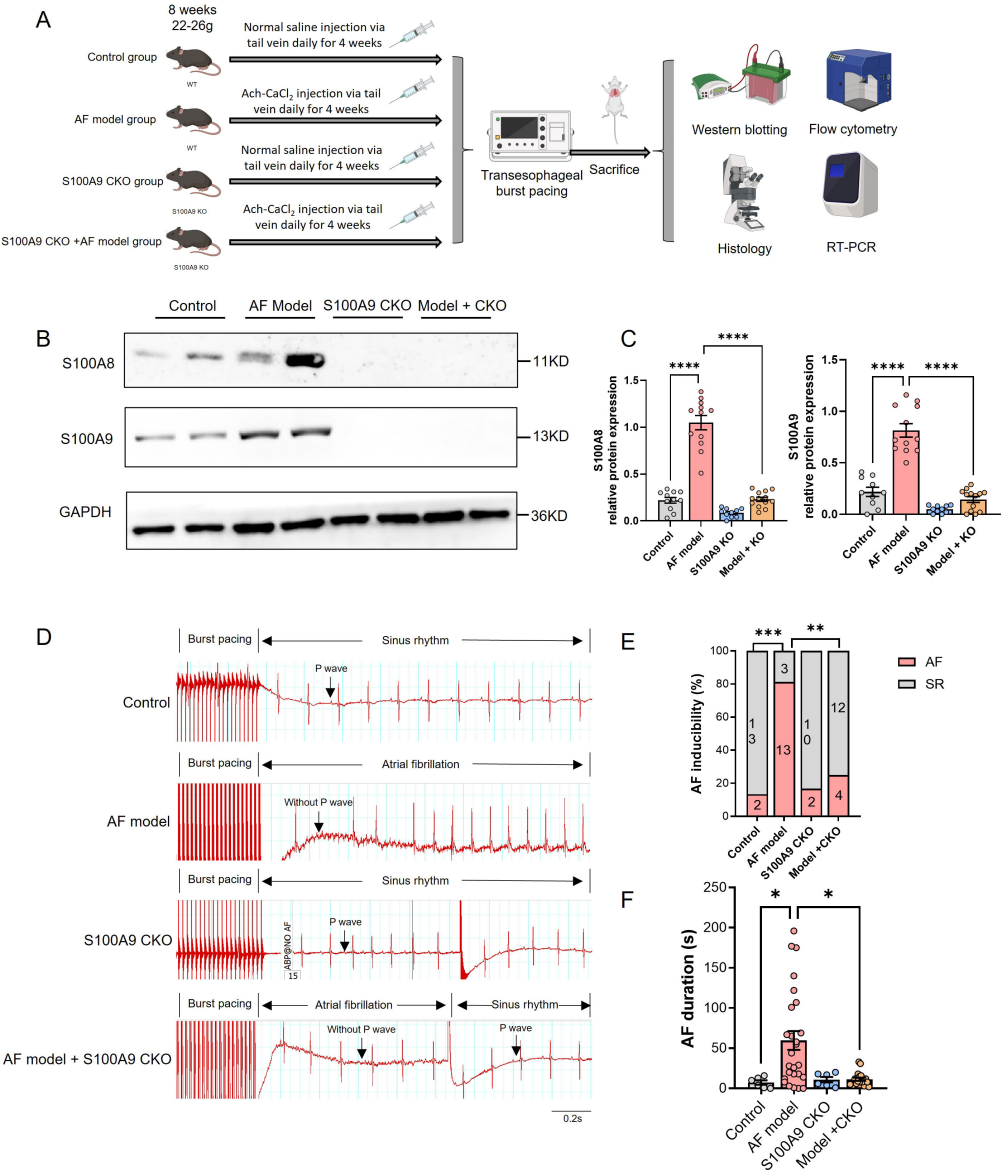
S100A9 CKO reduces AF inducibility and duration in a mouse model. **(A)** Experimental design showing the establishment of four groups: control, AF model (wild-type mice treated with Ach-CaCl2), S100A9 CKO (knockout mice treated with saline), and AF model + S100A9 CKO (knockout mice treated with Ach-CaCl2). **(B)** Western blot analysis of S100A8 and S100A9 protein levels in atrial tissues across the four groups. GAPDH served as a loading control. **(C)** Quantification of S100A8 and S100A9 protein expression levels, showing significant upregulation in the AF model group and attenuation in the S100A9 CKO groups (****P < 0.0001). **(D)** Representative electrocardiograms of mice after transesophageal burst pacing, demonstrating AF inducibility and recovery to SR in each group. **(E)** Percentage of AF inducibility across the groups, showing a significant reduction in the S100A9 CKO groups compared to the AF model group (**P < 0.01, ****P < 0.0001). **(F)** AF duration (in seconds) following burst pacing, significantly reduced in the S100A9 CKO groups compared to the AF model group (*P < 0.05, **P < 0.01, ***P < 0.001).

To elucidate the role of monocyte S100A8/A9 in the development of AF, we constructed S100A9 monocyte specific knockout (CKO) mice and established AF models through administration with Ach-CaCl_2_ for consecutive 4 weeks (**Figure 4A**). The immunoblotting results showed that S100A8 and S100A9 protein level of AF model group was increased compared with Control group, while S100A9 CKO could not only blocked S100A9 expression, but also the S100A8 level (**Figures 4B, C**).

The transesophageal pacing results are presented in **Figure 4**. Compared to other groups, the AF model group exhibited significantly higher AF inducibility and longer AF duration. ​​Importantly, while S100A9 CKO alone did not affect baseline AF susceptibility, it significantly reduced AF inducibility in the AF model group (**Figures 4D, E**).​​ Similarly, AF duration was markedly prolonged in the AF model group versus controls, but substantially shortened in AF model + S100A9 CKO mice (**Figure 4F**). These findings demonstrate that S100A9 CKO attenuates the Ach-CaCl_2_-induced increases in both AF susceptibility and duration.

### 3.5 S100A9 CKO mitigates atrial fibrosis and atrial dilation

**Figure 5:**
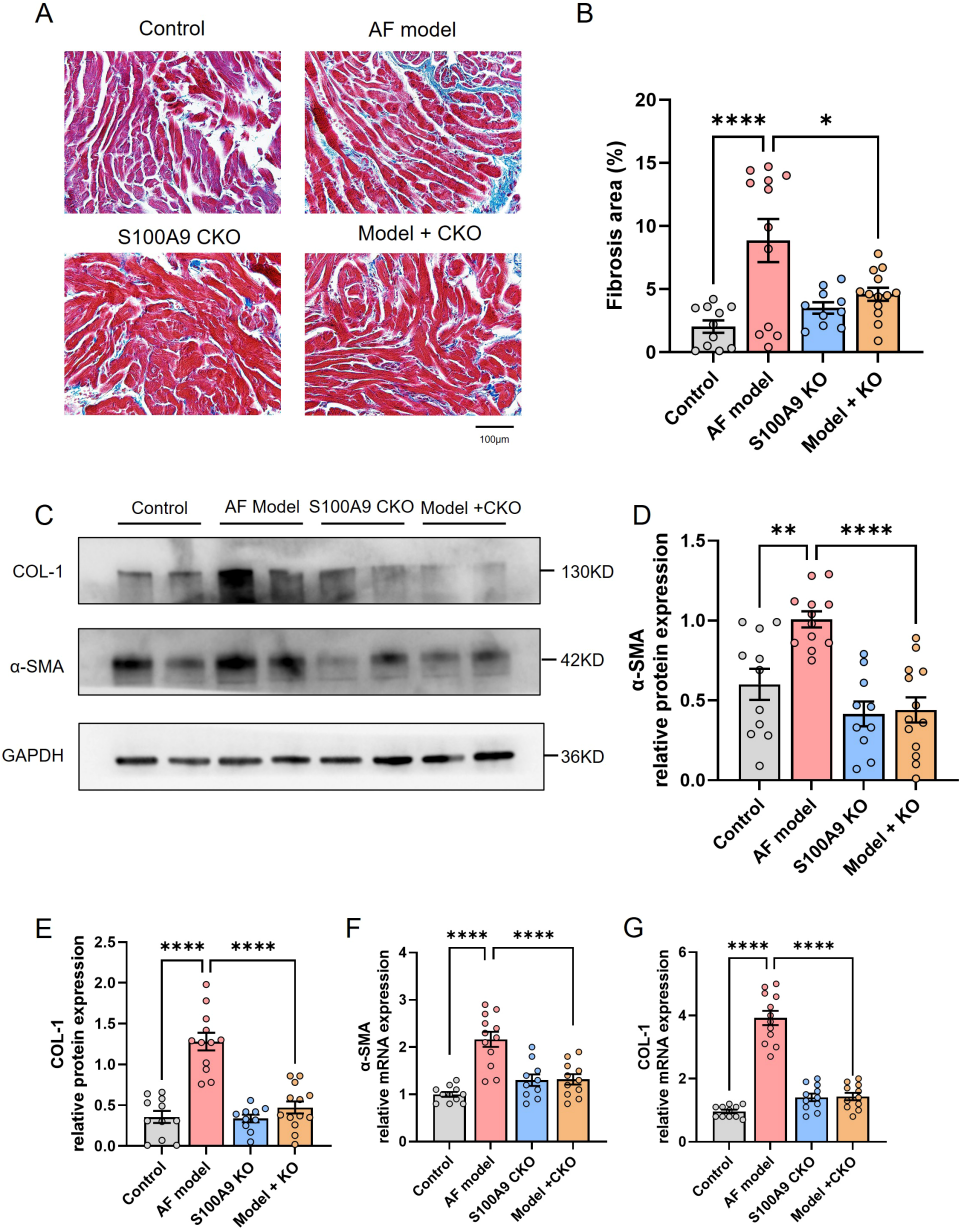
S100A9 CKO mitigates atrial fibrosis and reduces fibrosis-related protein expression in a mouse model of AF. **(A)** Representative Masson’s trichrome staining of atrial tissues showing increased fibrosis (blue staining) in the AF model group compared to the control group, with significant attenuation in S100A9 CKO groups. **(B)** Quantification of fibrotic areas (%) in atrial tissues, demonstrating a significant reduction in fibrosis in the S100A9 CKO groups (*P < 0.05, **P < 0.01, ****P < 0.0001). **(C)** Western blot analysis of fibrosis-related proteins, collagen-1 (COL-1) and alpha-smooth muscle actin (α-SMA), in the atrial tissues of the four groups. GAPDH was used as the loading control. **(D, E)** Quantification of COL-1 and α-SMA protein levels, showing a significant increase in the AF model group and attenuation in the S100A9 CKO groups (**P < 0.01, ****P < 0.0001). **(F, G)** Relative mRNA expression levels of COL-1 and α-SMA, with significant upregulation in the AF model group and downregulation in the S100A9 CKO groups (****P < 0.0001).

Since atrial fibrosis is a central feature of structural remodeling in AF, we conducted an assessment to determine whether S100A8/A9 had an impact on the development of atrial fibrosis. Masson staining revealed that the mice in the AF model group displayed noticeable atrial fibrosis in comparison to the control group, which was significantly reduced by the KO of S100A9 (**Figures 5A, B**). The zoomed out photos of the atrium and whole heart were shown in **Supplementary Figure S5**.

**Figure 6:**
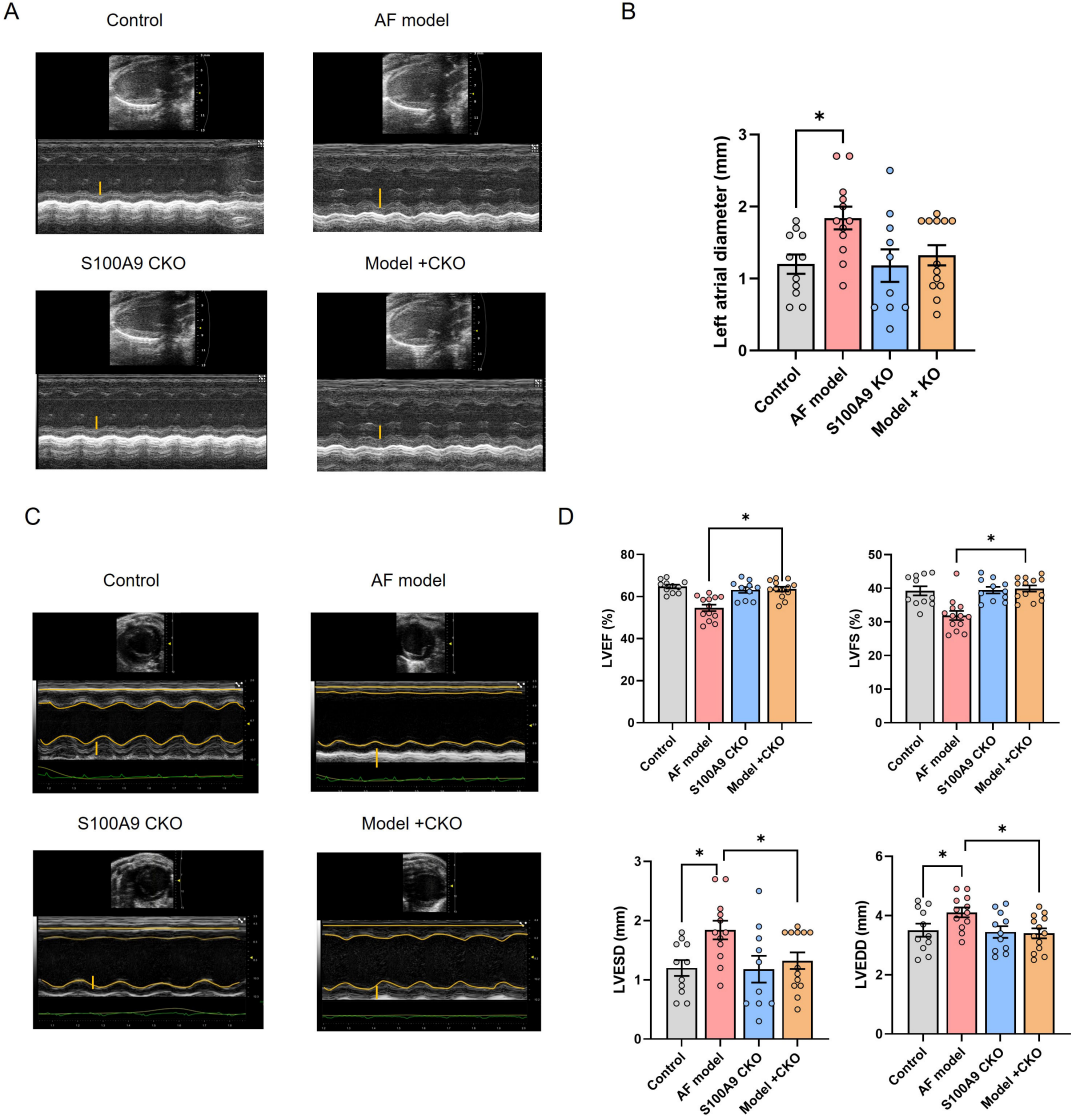
S100A9 CKO preserves cardiac function and attenuates remodeling in a mouse AF model. **(A)** Representative M-mode echocardiography of the left atrial chamber. **(B)** Quantification of ​​left atrial diameter (LAD, mm).​​ **(C)** Representative M-mode echocardiographic images of left ventricular function. **(D)** Cardiac function analysis: ​​Left ventricle ejection fraction (LVEF, %), fractional shortening (LVFS, %), end-systolic diameter (LVESD, mm), and end-diastolic diameter (LVEDD, mm) (*P < 0.05 vs. AF model).

Correspondingly, we examined the effect of S100A9 CKO on the protein levels of fibosis related protein collagen-1 and α-SMA in the atria of different groups. We observed increased levels of collagen-1 and α-SMA in the atria of AF model mice in comparison to controls. A significant decrease in the levels of these mRNAs and proteins was observed in mice of AF model+ S100A9 CKO group (**Figures 5C–G**). Echocardiography demonstrated that the AF model group had a significantly higher left atrial diameter (LAD) than the control group and S100A9 CKO group. The LAD of AF model+ S100A9 CKO group was lower than the AF model group but not significant (**Figures 6A, B**). AF mice exhibit ​​significant ventricular dilation (increased LVESD/LVEDD) and impaired contractility (decreased LVEF/LVFS)​​ versus controls. S100A9 CKO partially restores cardiac function (**Figures 6C, D**).

### 3.6 S100A9 CKO decreases immune cell infiltration and inflammation

**Figure 7:**
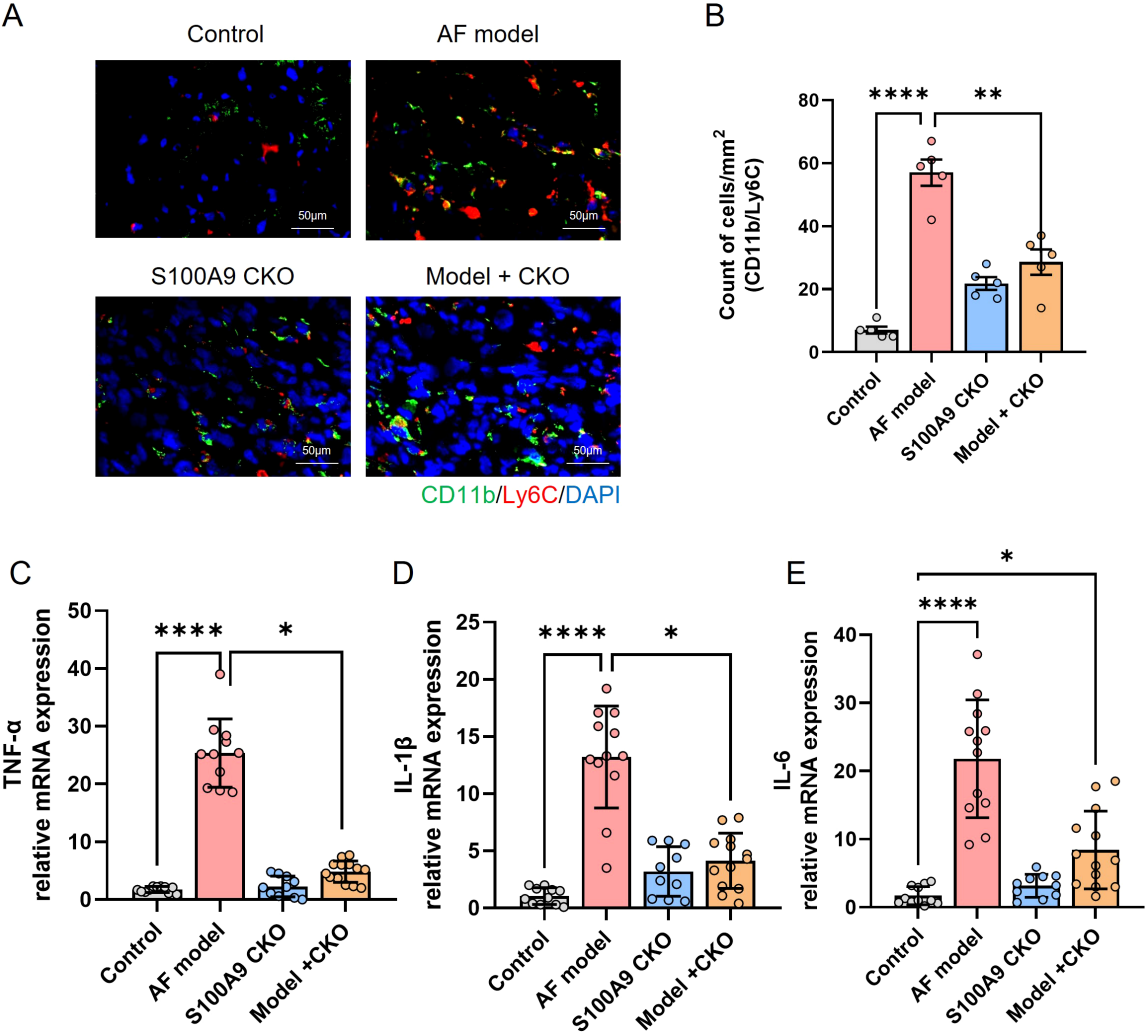
S100A9 CKO reduces immune cell infiltration and inflammatory cytokine expression in a mouse model of AF. **(A)** Representative immunohistochemical staining of CD11b/Ly6C monocytes in atrial tissues, showing increased infiltration in the AF model group (red arrowheads) and significant reduction in the S100A9 CKO groups. **(B)** Quantification of CD11b/Ly6C cell counts per mm² in atrial tissues, demonstrating a significant decrease in the S100A9 CKO groups (**P < 0.01, ****P < 0.0001). **(C–E)** Relative mRNA expression levels of pro-inflammatory cytokines (TNF-α, IL-1β, and IL-6) in atrial tissues, showing significant upregulation in the AF model group and suppression in the S100A9 CKO groups (*P < 0.05, **P < 0.01, ****P < 0.0001).

We further determined cell number and proportions of three monocyte subsets in four groups. The number of infiltrated CD11b/Ly6C+ cells was increased in AF model group and significantly attenuated by S100A9 CKO (**Figures 7A, B**). Moreover, the mRNA levels of inflammation cytokines including TNF-α, IL-1β, IL-6 were significantly increased in AF model group compared with the control group, while S100A9 CKO obviously inhibit the mRNA level of the cytokines after AF (**Figures 7C–E**).

### 3.7 S100A9 CKO inhibits TLR4/NF-κB pathway

**Figure 8:**
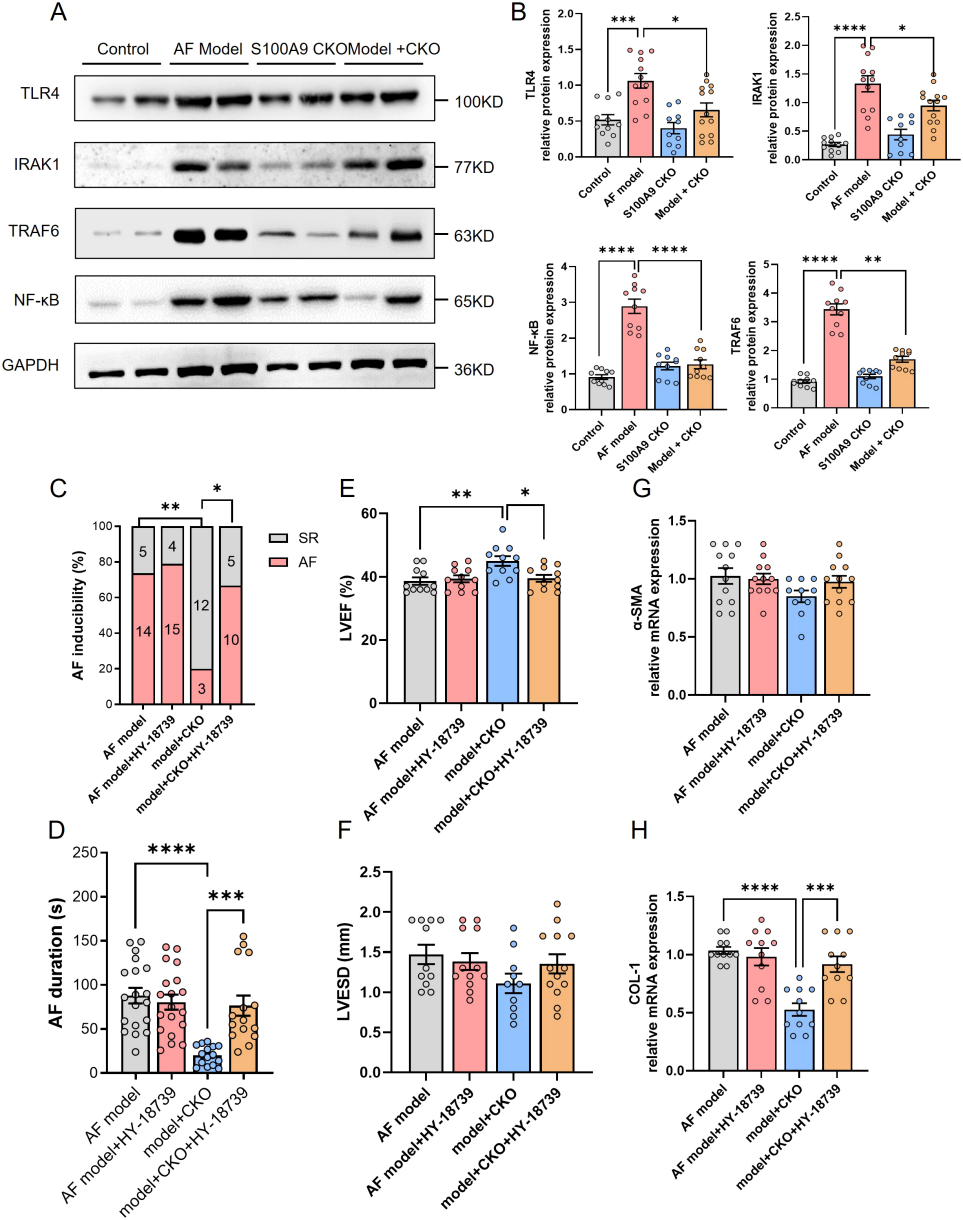
S100A9 CKO mitigates AF via suppression of the TLR4/NF-κB signaling pathway. **(A)** Western blot analysis of key proteins in the TLR4/NF-κB signaling pathway, including TLR4, IRAK1, TRAF6, and NF-κB, across the four groups (control, AF model, S100A9 CKO, and AF model + S100A9 CKO). GAPDH was used as the loading control. **(B)** Quantification of TLR4, IRAK1, TRAF6, and NF-κB protein expression, showing significant upregulation in the AF model group and attenuation in the S100A9 CKO groups (*P < 0.05, ***P < 0.001, ****P < 0.0001). **(C)** Percentage of AF inducibility in different groups, demonstrating the reversal of S100A9 CKO’s protective effects by the NF-κB activator HY-18739 (**P < 0.01). **(D)** Duration of AF episodes following burst pacing, showing that HY-18739 reversed the reduction in AF duration observed in the S100A9 CKO group (**P < 0.01). **(E, F)** Echocardiographic assessment of left ventricular ejection fraction (LVEF, %) and left ventricular end-systolic diameter (LVESD, mm), highlighting the protective effects of S100A9 CKO on cardiac function and ventricular remodeling, which were diminished by HY-18739 (*P < 0.05). **(G, H)** Relative mRNA expression levels of fibrosis-related markers, alpha-smooth muscle actin (α-SMA) and collagen-1 (COL-1), showing attenuation in the S100A9 CKO groups and partial reversal by HY-18739 (*P < 0.05, **P < 0.01).

To explore the precise underlying mechanisms, we investigated the Toll-like Receptor 4 (TLR4)/NF-κB pathways, which are known to be crucial regulators of immune cell infiltration and inflammation. Western blot analysis revealed a significant activation of TLR4/IRAK1/TRAF6/NF-κB in the atria of AF model mice compared to controls. However, this effect was markedly reduced byS100A9 CKO (**Figures 8A, B**). Further, NF-κB activator HY-18739 reverse the inhibition of S100A9 CKO on the AF inducibility and duration (**Figures 8C, D**). Echocardiography demonstrated that the cardiac protective effect of S100A9 CKO was canceled by HY-18739 (**Figures 8E, F**). Further, HY-18739 treatment also weaken the S100A9 CKO-induced suppression of collagen-1 and α-SMA mRNA (**Figures 8G, H**). These findings collectively suggest that S100A9 CKO leads to a reduction in AF, atrial structural remodeling, and cardiac fibrosis, at least partially through inhibiting the TLR4/NF-κB signaling pathways (See graphical abstract).

## 4 Discussion

**Figure 9:**
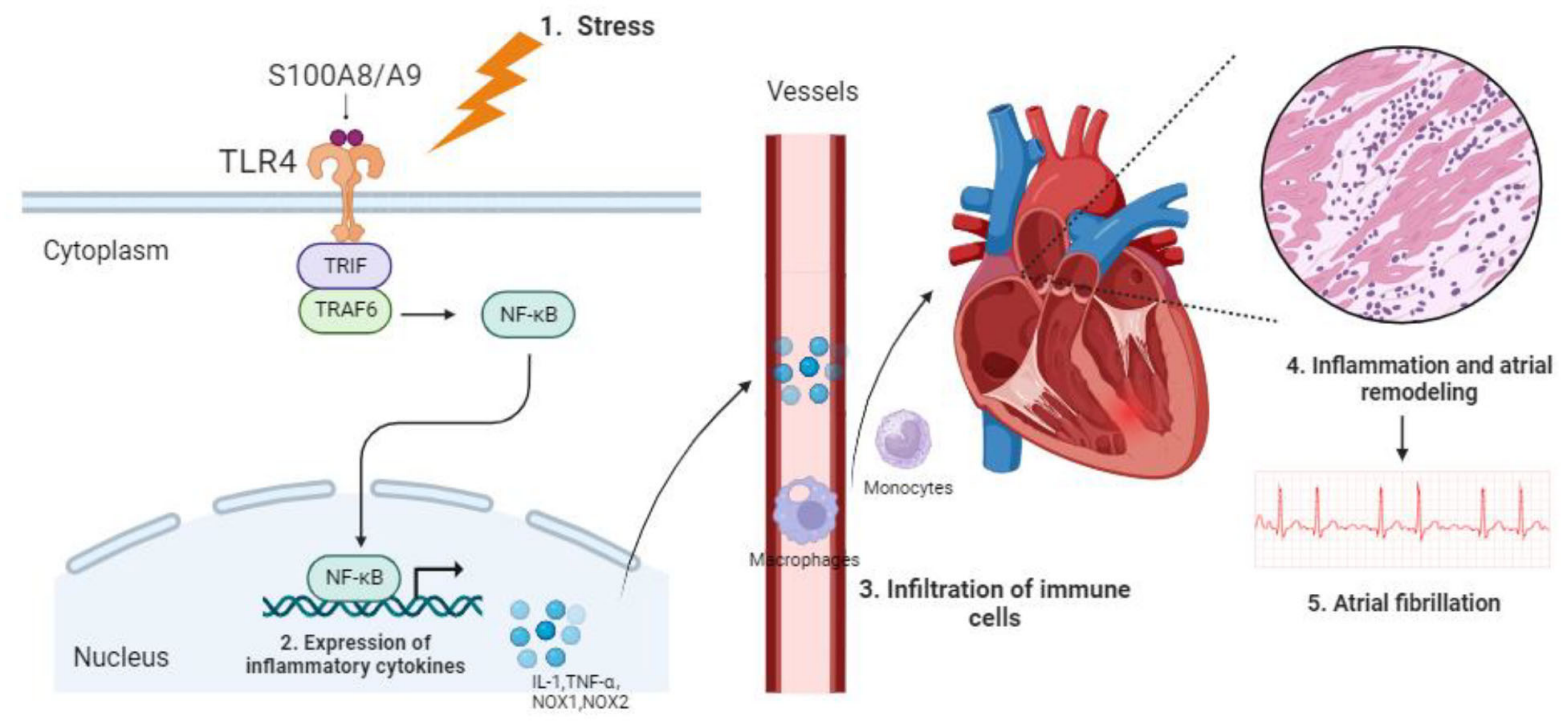
Proposed mechanism of S100A8/A9-mediated AF pathogenesis via the TLR4/NF-κB signaling pathway.

In this study, we leveraged publicly available data from the GEO database to identify potential key DEGs. Using Rank Aggregation of RRA analysis and PPI networks, nine DEGs were identified. Subsequent enrichment analysis singled out S100A8/A9 for in-depth examination due to its established association with inflammation and its crucial role in myocardial infarction. Notably, monocytes and macrophages, the predominant immune cells, exhibited strong associations with these DEGs. S100A8/A9 expression was markedly elevated in both AF patients and mouse models. Silencing of S100A9 notably suppressed the monocytes and inflammation, reversed structural remodeling, and conferred cardio-protection against AF via the TLR4/NF-κB pathway (**Figure 9**). Our findings suggest that S100A8/A9 might be a promising therapeutic target for AF.

Several studies leveraging GEO datasets have highlighted the heterogeneity of AF-associated hub genes, underscoring the disease’s complex molecular underpinnings. Zhang, J. et al. identified 27 DEGs across five datasets, prioritizing IGFBP2 and FHL2 as key regulators of AF pathology (33). Notably, despite methodological variations​​ (e.g., our exclusion of GSE31821 due to miRNA focus and GSE14975 for limited DEG yield), ​​both prior work and our analysis converge on inflammatory pathway activation as a central theme in AF.​​ Zhang, Y. F. et al. similarly reported NF-κB-driven inflammation via upregulated mediators like CXCR4 and SNAI2 (34), ​​aligning with our GO findings of neutrophil/leukocyte migration enrichment and KEGG-identified NF-κB signaling​​. This inflammatory consensus extends to immune cell dynamics​​—where prior studies implicated CYBB and CXCR2 in immune cell infiltration (35), ​​our study identifies S100A8/A9 as critical monocyte-derived mediators​​, ​​reinforcing the paradigm that myeloid inflammation fundamentally orchestrates AF progression​​.

S100A8/A9 heterodimers (MRP8/14) serve as critical calcium-dependent amplifiers of sterile inflammation, constitutively expressed in myeloid cells where they regulate cytoskeletal dynamics and eicosanoid metabolism (8). ​​In cardiovascular pathologies, these damage-associated molecular patterns (DAMPs) exhibit context-dependent roles:​​ Marinković et al. demonstrated that S100A9 inhibition improved early myocardial infarction recovery but paradoxically worsened late remodeling (36, 37), underscoring the temporal precision required for therapeutic targeting. This complexity extends to ischemic injury,​​ where Li et al. identified S100A8/A9 as early mediators of mitochondrial dysfunction via TLR4/Erk signaling – effects reversible by neutralizing antibodies (11) ​​Critically, elevated serum S100A8/A9 predicts adverse outcomes in acute coronary patients (11), establishing their translatable diagnostic value.​​ ​​Our study extends this paradigm to atrial fibrillation,​​ positioning S100A8/A9 not merely as biomarkers but as ​​orchestrators of monocyte-driven TLR4/NF-κB activation that perpetuates electromechanical remodeling​​ – a mechanism distinct from their acute ischemic roles yet converging on TLR4-dependent inflammation.

In light of our findings and those of Hulsmans et al (23), the involvement of immune cells—particularly monocyte/macrophage (MP) and dendritic cell (DC) populations—in the pathogenesis of AF is further clarified. We observed marked upregulation of S100A8/A9 in non-cardiomyocyte atrial cells from AF patients, with particularly high expression in MP/DC clusters, underscoring a key immune-mediated mechanism underlying atrial remodeling. This parallels Hulsmans et al.’s identification of expanded inflammatory monocytes and SPP1(+) macrophages in AF, suggesting a convergent inflammatory pathway driving both atrial inflammation and fibrosis. Importantly, the suppression of AF burden and structural remodeling in our S100A9 knockout mouse model aligns closely with their findings, where inhibition of monocyte trafficking also reduced AF susceptibility. Together, these results emphasize the centrality of immune cell infiltration and pro-inflammatory signaling—particularly via TLR4/NF-κB activation driven by S100A8/A9 and SPP1—as promising mechanistic targets for therapeutic intervention in AF.

Although no prior studies have directly explored the role of S100A8/A9 in AF, our integrative analysis and experimental validation suggest that this complex may serve as a key mediator in AF pathogenesis (38). Bioinformatic data revealed elevated S100A8/A9 expression in AF, correlating with increased monocyte abundance—prompting the hypothesis that S100A8/A9 promotes AF by enhancing monocyte recruitment and pro-inflammatory cytokine release. This was substantiated by both human and murine data, where S100A8/A9 expression was significantly upregulated in AF patients and Ach-CaCl_2_–induced AF mice compared to sinus rhythm controls. Genetic ablation of S100A9 not only reduced the levels of both S100A8 and S100A9—consistent with the known interdependence of these proteins for complex stability (39). —but also markedly suppressed AF susceptibility and duration. S100A8 knockout was avoided due to its association with embryonic lethality. Notably, S100A9-deficient mice exhibited resistance to Ach-CaCl_2_–induced atrial fibrosis and dilation. This aligns with the established link between AF and atrial fibrosis, a pathological remodeling process driven by excessive extracellular matrix deposition that disrupts electrical conduction and promotes arrhythmia persistence (40). The observed protective effects support the notion that atrial fibrosis in AF is closely tied to immune cell infiltration and cytokine-mediated inflammation (41), processes in which S100A8/A9 plays a central role.

Immunohistochemical analysis confirmed significant monocyte infiltration in atrial tissue of the AF model, supporting the pro-inflammatory role of S100A8/A9. Correspondingly, deletion of S100A9 markedly reduced expression of key inflammatory mediators, including TNF-α, IL-1β, and IL-6. KEGG pathway enrichment implicated the NF-κB signaling pathway as a central node in AF-associated inflammation. Given the established interaction between S100A8/A9 and TLR4, we explored the TLR4/NF-κB axis as a potential downstream effector. TLR4, a pattern recognition receptor integral to innate immunity (42), activates NF-κB through adaptor molecules such as IRAK1 and TRAF6, thereby amplifying pro-inflammatory gene expression (43). In our study, S100A9 knockout significantly attenuated activation of this pathway in response to Ach-CaCl_2_ stimulation. Moreover, pharmacologic reactivation of NF-κB using HY-18739 reversed the protective effects conferred by S100A9 deletion, restoring AF inducibility, impairing cardiac function, and increasing fibrosis markers such as collagen-1 and α-SMA. These findings suggest that the cardioprotective effects of S100A9 knockout are mediated, at least in part, through suppression of TLR4/NF-κB signaling, reinforcing this pathway’s central role in AF pathophysiology.

This study has several limitations. First, while we used a LysM-Cre system to delete S100A9 in myeloid cells, this approach does not distinguish between monocytes, macrophages, and neutrophils, limiting our ability to pinpoint the precise cellular contributors to AF pathology. Second, the Ach-CaCl_2_–induced AF model primarily reflects vagally mediated acute AF episodes and may not capture the complexity of chronic or comorbidity-driven AF, such as that associated with hypertension or metabolic syndrome. Third, we only examined male mice at a single time point, without assessing sex differences or the temporal dynamics of S100A8/A9 expression and its downstream effects. Fourth, our use of the NF-κB activator HY-18739 as a pharmacological rescue tool does not fully replicate endogenous TLR4-mediated activation and may have off-target effects. Finally, while our data strongly support the involvement of S100A8/A9 in immune cell–driven atrial remodeling, we did not explore the role of other immune cell types, such as dendritic cells or lymphocytes, nor did we assess the therapeutic efficacy of pharmacological S100A8/A9 blockade—areas that warrant further investigation.

To summarize, this investigation reveals a pronounced up-regulation of S100A8/A9 in AF patients as well as in an Ach-CaCl_2_-induced mouse model. This elevation is linked to heightened monocyte infiltration, increased atrial inflammation, and fibrosis. The utilization of S100A9 KO notably mitigates atrial structural alterations and susceptibility to AF, primarily through the suppression of the pro-inflammatory TLR4/NF-κB signaling pathway. Our findings posit that targeting S100A8/A9 might serve as a promising therapeutic approach for AF. Nonetheless, a more comprehensive exploration through functional studies is essential to elucidate the underlying mechanisms in depth.

## Data Availability

The original contributions presented in the study are included in the article/**Supplementary Material**. Further inquiries can be directed to the corresponding authors.

## Supplementary material

Supplementary Figure 1 Flow chart of the bioinformatic analysis and experimental validation.

Supplementary Figure 2 Visualization of DEGs between AF and SR patients. **(A–C)** Volcano plot of DEGs in GSE41177, GSE79768, GSE115574; **(D–F)** Clustered heatmap of DEGs in GSE41177, GSE79768, GSE115574; G-H Venn diagram of DEGs in GSE41177, GSE79768, GSE115574.

Supplementary Figure 3 UMAP plots showing S100A8 and S100A9 expression in non-cardiomyocyte cell types from control and AF patients.

Supplementary Figure 4 Immune cell infiltration analysis and correlation with hub gene expression in AF. **(A)** Heatmap showing the abundance of various immune cell types in atrial tissues from AF and SR patients. Red indicates higher immune cell infiltration, while blue indicates lower infiltration. **(B)** Boxplot of tumor microenvironment (TME) immune cell content, illustrating the relative proportions of immune cell types, including monocytes, macrophages, neutrophils, and T cells, between AF and SR samples. Monocytes and macrophages are more abundant in AF samples. **(C)** Correlation matrix heatmap depicting the relationship between hub gene expression (e.g., S100A8, S100A9, TYROBP) and immune cell infiltration scores. Positive correlations are shown in orange, and negative correlations are shown in blue, with significance indicated by asterisks (*P < 0.05, **P < 0.01, ***P < 0.001). S100A8 and S100A9 exhibit significant positive correlations with monocyte and macrophage infiltration.

Supplementary Figure 5 The zoomed out photos of the atrium and whole heart stained by Masson’s trichrome staining. Scale bar 200 μm and 100 μm.

## References

[1] Brundel B Ai X Hills MT Kuipers MF Lip GYH de Groot NMS. Atrial fibrillation. Nat Rev Dis Primers. (2022) 8:21. doi: 10.1038/s41572-022-00347-9, PMID: 35393446

[2] Kornej J Börschel CS Benjamin EJ Schnabel RB. Epidemiology of atrial fibrillation in the 21st century: novel methods and new insights. Circ Res. (2020) 127:4–20. doi: 10.1161/CIRCRESAHA.120.316340, PMID: 32716709 PMC7577553

[3] Vinciguerra M Dobrev D Nattel S. Atrial fibrillation: pathophysiology, genetic and epigenetic mechanisms. Lancet Reg Health Eur. (2024) 37:100785. doi: 10.1016/j.lanepe.2023.100785, PMID: 38362554 PMC10866930

[4] Dobrev D Heijman J Hiram R Li N Nattel S. Inflammatory signalling in atrial cardiomyocytes: a novel unifying principle in atrial fibrillation pathophysiology. Nat Rev Cardiol. (2023) 20:145–67. doi: 10.1038/s41569-022-00759-w, PMID: 36109633 PMC9477170

[5] Ajoolabady A Nattel S Lip GYH Ren J. Inflammasome signaling in atrial fibrillation: JACC state-of-the-art review. J Am Coll Cardiol. (2022) 79:2349–66. doi: 10.1016/j.jacc.2022.03.379, PMID: 35680186 PMC8972346

[6] Yao Y Yang M Liu D Zhao Q. Immune remodeling and atrial fibrillation. Front Physiol. (2022) 13:927221. doi: 10.3389/fphys.2022.927221, PMID: 35936905 PMC9355726

[7] Wong KL Tai JJ Wong WC Han H Sem X Yeap W. Gene expression profiling reveals the defining features of the classical, intermediate, and nonclassical human monocyte subsets. Blood. (2011) 118:e16–31. doi: 10.1182/blood-2010-12-326355, PMID: 21653326

[8] Pruenster M Vogl T Roth J Sperandio M. S100A8/A9: From basic science to clinical application. Pharmacol Ther. (2016) 167:120–31. doi: 10.1016/j.pharmthera.2016.07.015, PMID: 27492899

[9] Wang S Song R Wang Z Jing Z Wang S Ma J. S100A8/A9 in inflammation. Front Immunol. (2018) 9:1298. doi: 10.3389/fimmu.2018.01298, PMID: 29942307 PMC6004386

[10] Sprenkeler EGG Zandstra J van Kleef ND Goetschalckx I Verstegen B Aarts CEM. S100A8/A9 is a marker for the release of neutrophil extracellular traps and induces neutrophil activation. Cells. (2022) 11. doi: 10.3390/cells11020236, PMID: 35053354 PMC8773660

[11] Li Y Chen B Yang X Zhang C Jiao Y Li P. S100a8/a9 signaling causes mitochondrial dysfunction and cardiomyocyte death in response to ischemic/reperfusion injury. Circulation. (2019) 140:751–64. doi: 10.1161/CIRCULATIONAHA.118.039262, PMID: 31220942

[12] Cai Z Xie Q Hu T Yao Q Zhao J Wu Q. S100A8/A9 in myocardial infarction: A promising biomarker and therapeutic target. Front Cell Dev Biol. (2020) 8:603902. doi: 10.3389/fcell.2020.603902, PMID: 33282877 PMC7688918

[13] Yeh YH Kuo CT Lee YS Lin YM Nattel S Tsai FC. Region-specific gene expression profiles in the left atria of patients with valvular atrial fibrillation. Heart Rhythm. (2013) 10:383–91. doi: 10.1016/j.hrthm.2012.11.013, PMID: 23183193

[14] Tsai FC Lin YC Chang SH Chang GJ Hsu YJ Lin YM. Differential left-to-right atria gene expression ratio in human sinus rhythm and atrial fibrillation: Implications for arrhythmogenesis and thrombogenesis. Int J Cardiol. (2016) 222:104–12. doi: 10.1016/j.ijcard.2016.07.103, PMID: 27494721

[15] Çubukçuoğlu Deniz G Durdu S Doğan Y Erdemli E Özdağ H Akar AR. Molecular signatures of human chronic atrial fibrillation in primary mitral regurgitation. Cardiovasc Ther. (2021) 2021:5516185. doi: 10.1155/2021/5516185, PMID: 34737791 PMC8538404

[16] Gustavsson EK Zhang D Reynolds RH Garcia-Ruiz S Ryten M. ggtranscript: an R package for the visualization and interpretation of transcript isoforms using ggplot2. Bioinformatics. (2022) 38:3844–6. doi: 10.1093/bioinformatics/btac409, PMID: 35751589 PMC9344834

[17] Gu Z Eils R Schlesner M. Complex heatmaps reveal patterns and correlations in multidimensional genomic data. Bioinformatics. (2016) 32:2847–9. doi: 10.1093/bioinformatics/btw313, PMID: 27207943

[18] Peng C Zhang Y Lang X Zhang Y. Role of mitochondrial metabolic disorder and immune infiltration in diabetic cardiomyopathy: new insights from bioinformatics analysis. J Transl Med. (2023) 21:66. doi: 10.1186/s12967-023-03928-8, PMID: 36726122 PMC9893675

[19] Yu G Wang LG Han Y He QY. clusterProfiler: an R package for comparing biological themes among gene clusters. Omics. (2012) 16:284–7. doi: 10.1089/omi.2011.0118, PMID: 22455463 PMC3339379

[20] Szklarczyk D Gable AL Nastou KC Lyon D Kirsch R Pyysalo S. The STRING database in 2021: customizable protein-protein networks, and functional characterization of user-uploaded gene/measurement sets. Nucleic Acids Res. (2021) 49:D605–d12. doi: 10.1093/nar/gkaa1074, PMID: 33237311 PMC7779004

[21] Doncheva NT Morris JH Gorodkin J Jensen LJ. Cytoscape stringApp: network analysis and visualization of proteomics data. J Proteome Res. (2019) 18:623–32. doi: 10.1021/acs.jproteome.8b00702, PMID: 30450911 PMC6800166

[22] Miao YR Zhang Q Lei Q Luo M Xie GY Wang H. ImmuCellAI: A unique method for comprehensive T-cell subsets abundance prediction and its application in cancer immunotherapy. Adv Sci (Weinh). (2020) 7:1902880. doi: 10.1002/advs.201902880, PMID: 32274301 PMC7141005

[23] Hulsmans M Schloss MJ Lee IH Bapat A Iwamoto Y Vinegoni C. Recruited macrophages elicit atrial fibrillation. Science. (2023) 381:231–9. doi: 10.1126/science.abq3061, PMID: 37440641 PMC10448807

[24] Liu X Wang J Jin J Hu Q Zhao T Wang J. S100A9 deletion in microglia/macrophages ameliorates brain injury through the STAT6/PPARγ pathway in ischemic stroke. CNS Neurosci Ther. (2024) 30:e14881. doi: 10.1111/cns.14881, PMID: 39107960 PMC11303267

[25] Skarnes WC Rosen B West AP Koutsourakis M Bushell W Iyer V. A conditional knockout resource for the genome-wide study of mouse gene function. Nature. (2011) 474:337–42. doi: 10.1038/nature10163, PMID: 21677750 PMC3572410

[26] Liu P Sun H Zhou X Wang Q Gao F Fu Y. CXCL12/CXCR4 axis as a key mediator in atrial fibrillation via bioinformatics analysis and functional identification. Cell Death Dis. (2021) 12:813. doi: 10.1038/s41419-021-04109-5, PMID: 34453039 PMC8397768

[27] Schrickel JW Bielik H Yang A Schimpf R Shlevkov N Burkhardt D. Induction of atrial fibrillation in mice by rapid transesophageal atrial pacing. Basic Res Cardiol. (2002) 97:452–60. doi: 10.1007/s003950200052, PMID: 12395207

[28] Wang Q Yuan J Shen H Zhu Q Chen B Wang J. Calpain inhibition protects against atrial fibrillation by mitigating diabetes-associated atrial fibrosis and calcium handling dysfunction in type 2 diabetes mice. Heart Rhythm. (2024) 21:1143–51. doi: 10.1016/j.hrthm.2024.02.036, PMID: 38395244

[29] Wen HC Huo YN Chou CM Lee WS. PMA inhibits endothelial cell migration through activating the PKC-δ/Syk/NF-κB-mediated up-regulation of Thy-1. Sci Rep. (2018) 8:16247. doi: 10.1038/s41598-018-34548-8, PMID: 30389973 PMC6214930

[30] Kim G Lee SY Oh S Jang JW Lee J Kim HS. Anti-inflammatory effects of extracellular vesicles from ecklonia cava on 12-O-tetradecanoylphorbol-13-acetate-induced skin inflammation in mice. Int J Mol Sci. (2024) 25. doi: 10.3390/ijms252312522, PMID: 39684233 PMC11641720

[31] Cao W Huang L Yu H Qian Y Liu L Xu M. Calycosin extracted from Astragali Radix reduces NETs formation to improve renal fibrosis via TLR4/NF-κB pathway. J Ethnopharmacol. (2025) 342:119391. doi: 10.1016/j.jep.2025.119391, PMID: 39855434

[32] Pan L Yu L Wang L He J Sun J Wang X. Inflammatory stimuli promote oxidative stress in pancreatic acinar cells via Toll-like receptor 4/nuclear factor-κB pathway. Int J Mol Med. (2018) 42:3582–90. doi: 10.3892/ijmm.2018.3906, PMID: 30272284

[33] Zhang J Huang X Wang X Gao Y Liu L Li Z. Identification of potential crucial genes in atrial fibrillation: a bioinformatic analysis. BMC Med Genomics. (2020) 13:104. doi: 10.1186/s12920-020-00754-5, PMID: 32682418 PMC7368672

[34] Zhang YF Meng LB Hao ML Li XY Zou T. CXCR4 and TYROBP mediate the development of atrial fibrillation via inflammation. J Cell Mol Med. (2022) 26:3557–67. doi: 10.1111/jcmm.17405, PMID: 35607269 PMC9189330

[35] Wu LD Li F Chen JY Zhang J Qian LL Wang RX. Analysis of potential genetic biomarkers using machine learning methods and immune infiltration regulatory mechanisms underlying atrial fibrillation. BMC Med Genomics. (2022) 15:64. doi: 10.1186/s12920-022-01212-0, PMID: 35305619 PMC8934464

[36] Marinković G Koenis DS de Camp L Jablonowski R Graber N de Waard V. S100A9 links inflammation and repair in myocardial infarction. Circ Res. (2020) 127:664–76. doi: 10.1161/CIRCRESAHA.120.315865, PMID: 32434457

[37] Marinković G Grauen Larsen H Yndigegn T Szabo IA Mares RG de Camp L. Inhibition of pro-inflammatory myeloid cell responses by short-term S100A9 blockade improves cardiac function after myocardial infarction. Eur Heart J. (2019) 40:2713–23. doi: 10.1093/eurheartj/ehz461, PMID: 31292614

[38] Suzuki A Fukuzawa K Yamashita T Yoshida A Sasaki N Emoto T. Circulating intermediate CD14++CD16+monocytes are increased in patients with atrial fibrillation and reflect the functional remodelling of the left atrium. Europace. (2017) 19:40–7. doi: 10.1093/europace/euv422, PMID: 26826137

[39] Passey RJ Williams E Lichanska AM Wells C Hu S Geczy CL. A null mutation in the inflammation-associated S100 protein S100A8 causes early resorption of the mouse embryo. J Immunol. (1999) 163:2209–16. doi: 10.4049/jimmunol.163.4.2209, PMID: 10438963

[40] Sagris M Vardas EP Theofilis P Antonopoulos AS Oikonomou E Tousoulis D. Atrial fibrillation: pathogenesis, predisposing factors, and genetics. Int J Mol Sci. (2021) 23. doi: 10.3390/ijms23010006, PMID: 35008432 PMC8744894

[41] Jalife J Kaur K. Atrial remodeling, fibrosis, and atrial fibrillation. Trends Cardiovasc Med. (2015) 25:475–84. doi: 10.1016/j.tcm.2014.12.015, PMID: 25661032 PMC5658790

[42] Fitzgerald KA Kagan JC. Toll-like receptors and the control of immunity. Cell. (2020) 180:1044–66. doi: 10.1016/j.cell.2020.02.041, PMID: 32164908 PMC9358771

[43] Mitchell S Vargas J Hoffmann A. Signaling via the NFκB system. Wiley Interdiscip Rev Syst Biol Med. (2016) 8:227–41. doi: 10.1002/wsbm.1331, PMID: 26990581 PMC8363188

