# Supplementary figures and images for: Myeloid-specific S100A8/A9 deficiency attenuates atrial fibrillation through prevention of TLR4/NF-kB-mediated immune cell recruitment and inflammation
Source: Front Immunol. 2025 Sep 4;16:1623486. doi: 10.3389/fimmu.2025.1623486 (PMC12443547; doi:10.3389/fimmu.2025.1623486)

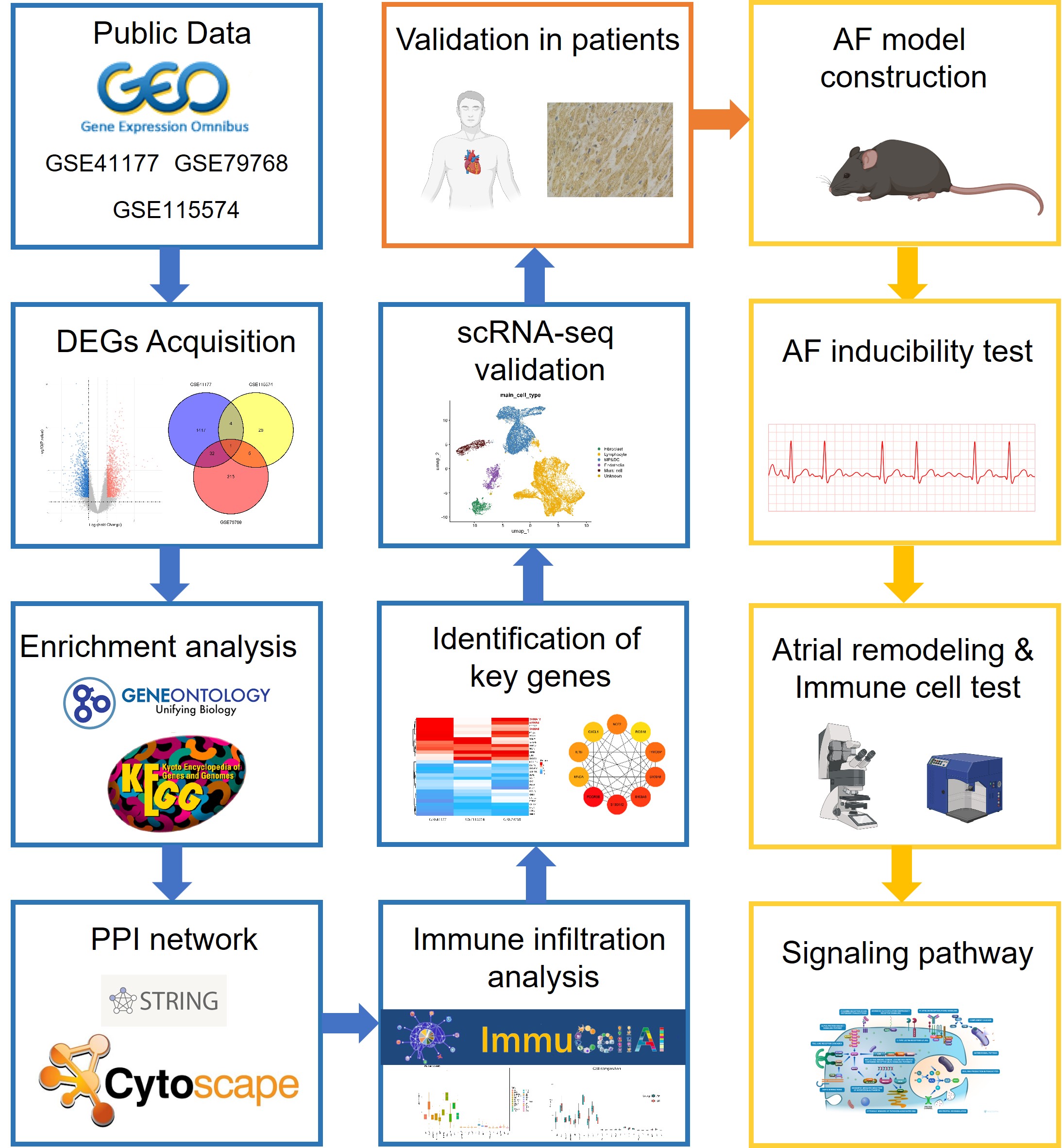

Supplement: Supplementary Figure 1 — Flow chart of the bioinformatic analysis and experimental validation. [file Image1.jpeg]

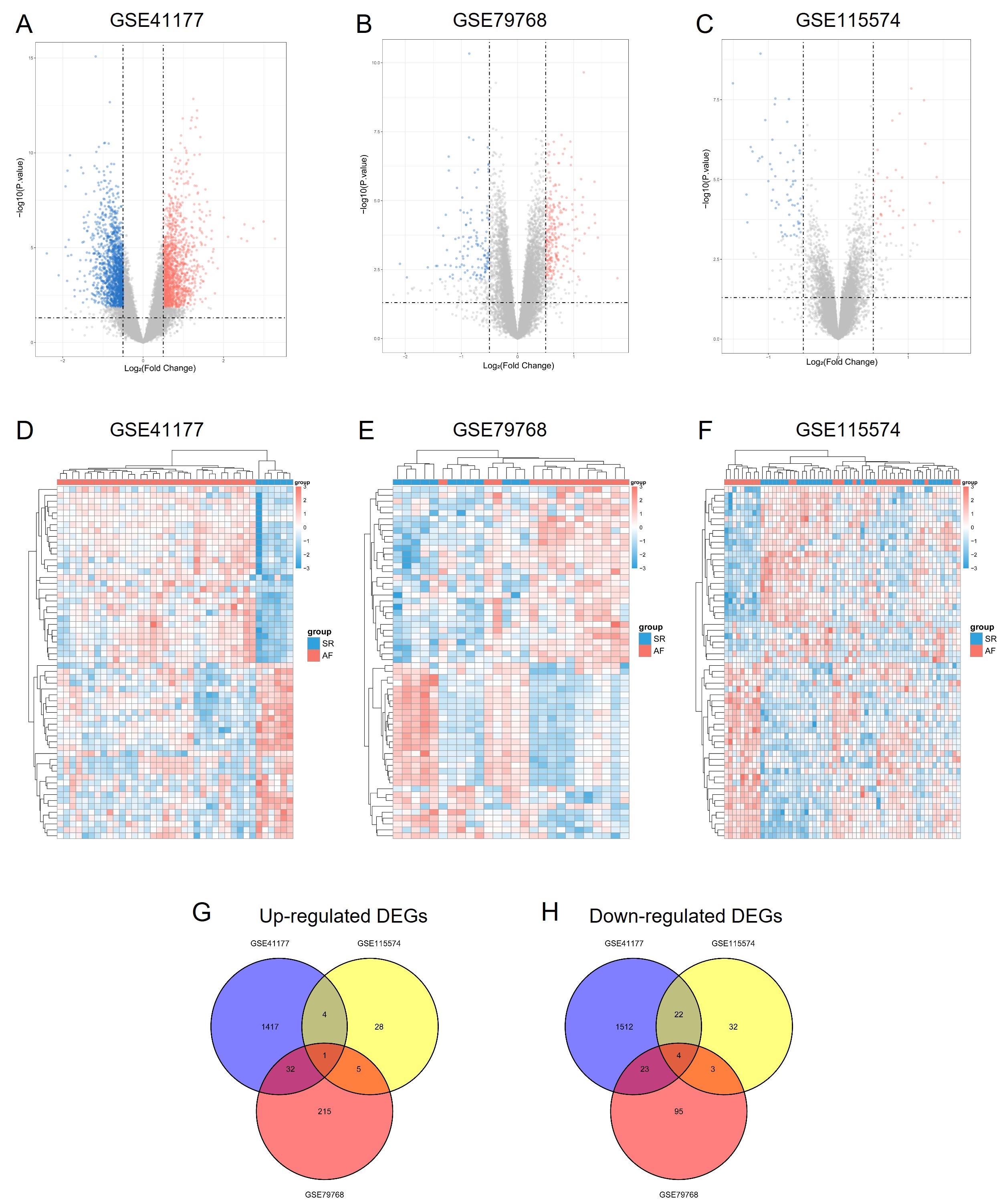

Supplement: Supplementary Figure 2 — Visualization of DEGs between AF and SR patients. (A–C) Volcano plot of DEGs in GSE41177, GSE79768, GSE115574; (D–F) Clustered heatmap of DEGs in GSE41177, GSE79768, GSE115574; G-H Venn diagram of DEGs in GSE41177, GSE79768, GSE115574. [file Image2.jpeg]

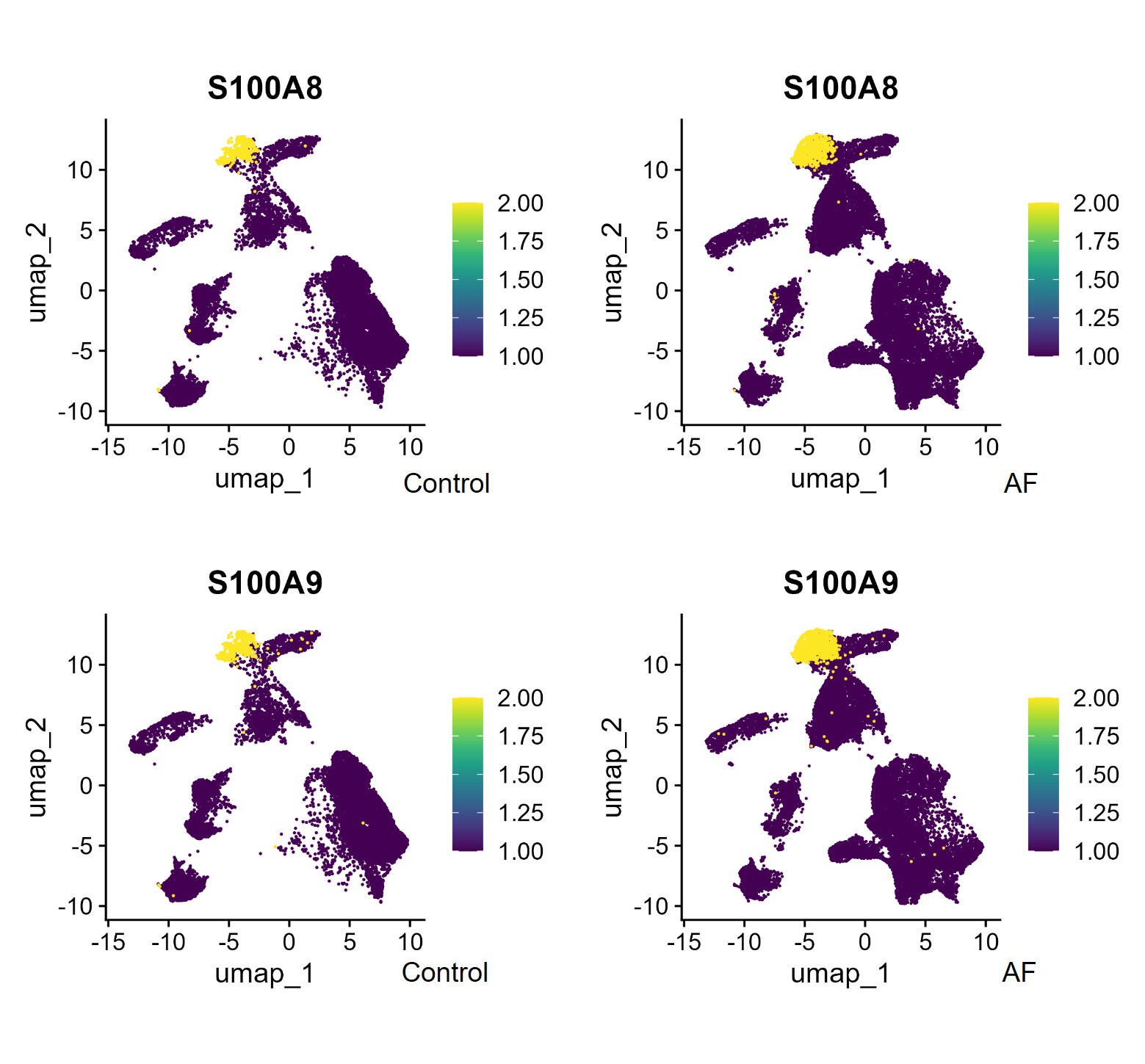

Supplement: Supplementary Figure 3 — UMAP plots showing S100A8 and S100A9 expression in non-cardiomyocyte cell types from control and AF patients. [file Image3.jpeg]

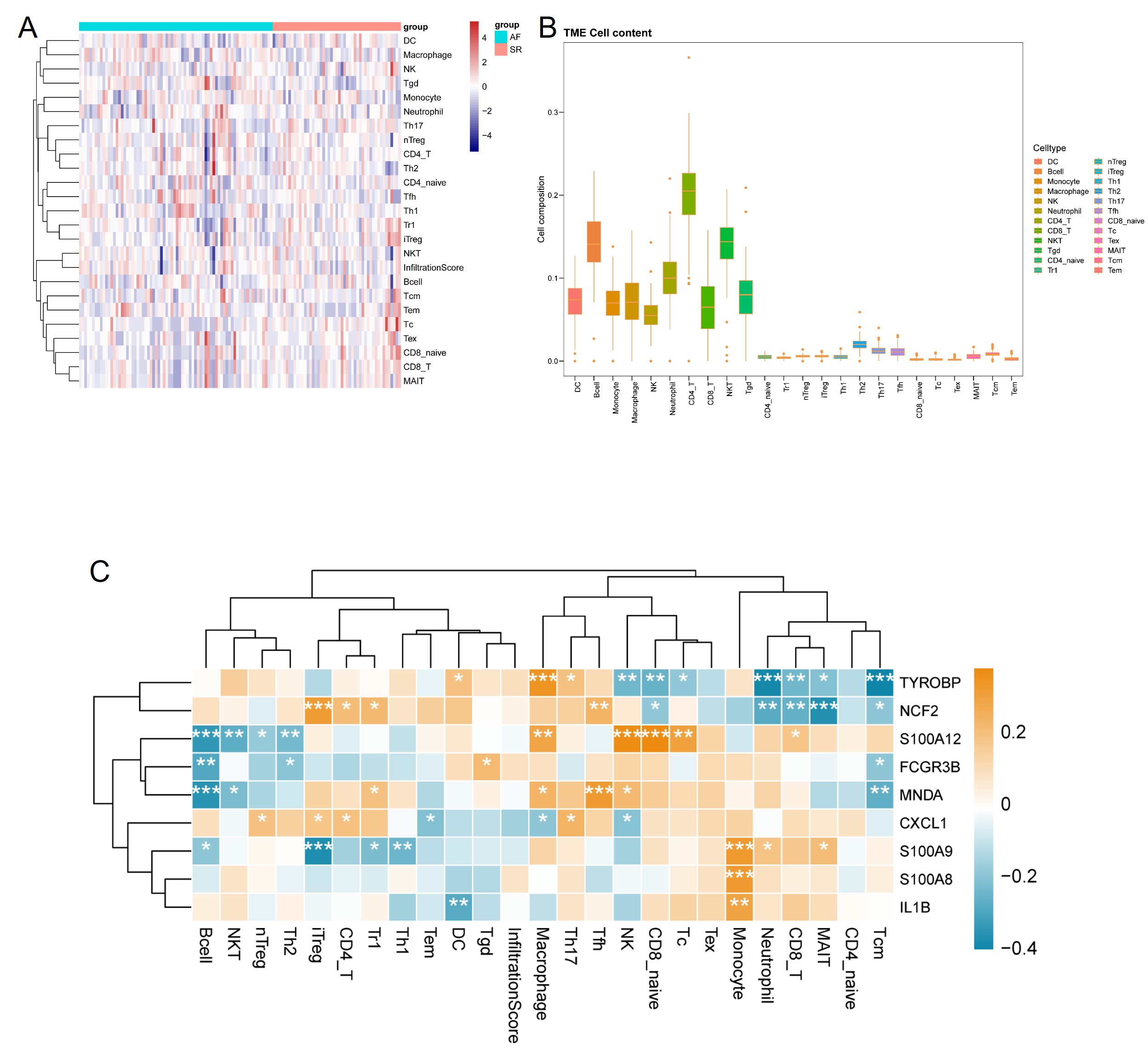

Supplement: Supplementary Figure 4 — Immune cell infiltration analysis and correlation with hub gene expression in AF. (A) Heatmap showing the abundance of various immune cell types in atrial tissues from AF and SR patients. Red indicates higher immune cell infiltration, while blue indicates lower infiltration. (B) Boxplot of tumor microenvironment (TME) immune cell content, illustrating the relative proportions of immune cell types, including monocytes, macrophages, neutrophils, and T cells, between AF and SR samples. Monocytes and macrophages are more abundant in AF samples. (C) Correlation matrix heatmap depicting the relationship between hub gene expression (e.g., S100A8, S100A9, TYROBP) and immune cell infiltration scores. Positive correlations are shown in orange, and negative correlations are shown in blue, with significance indicated by asterisks (*P < 0.05, **P < 0.01, ***P < 0.001). S100A8 and S100A9 exhibit significant positive correlations with monocyte and macrophage infiltration. [file Image4.jpeg]

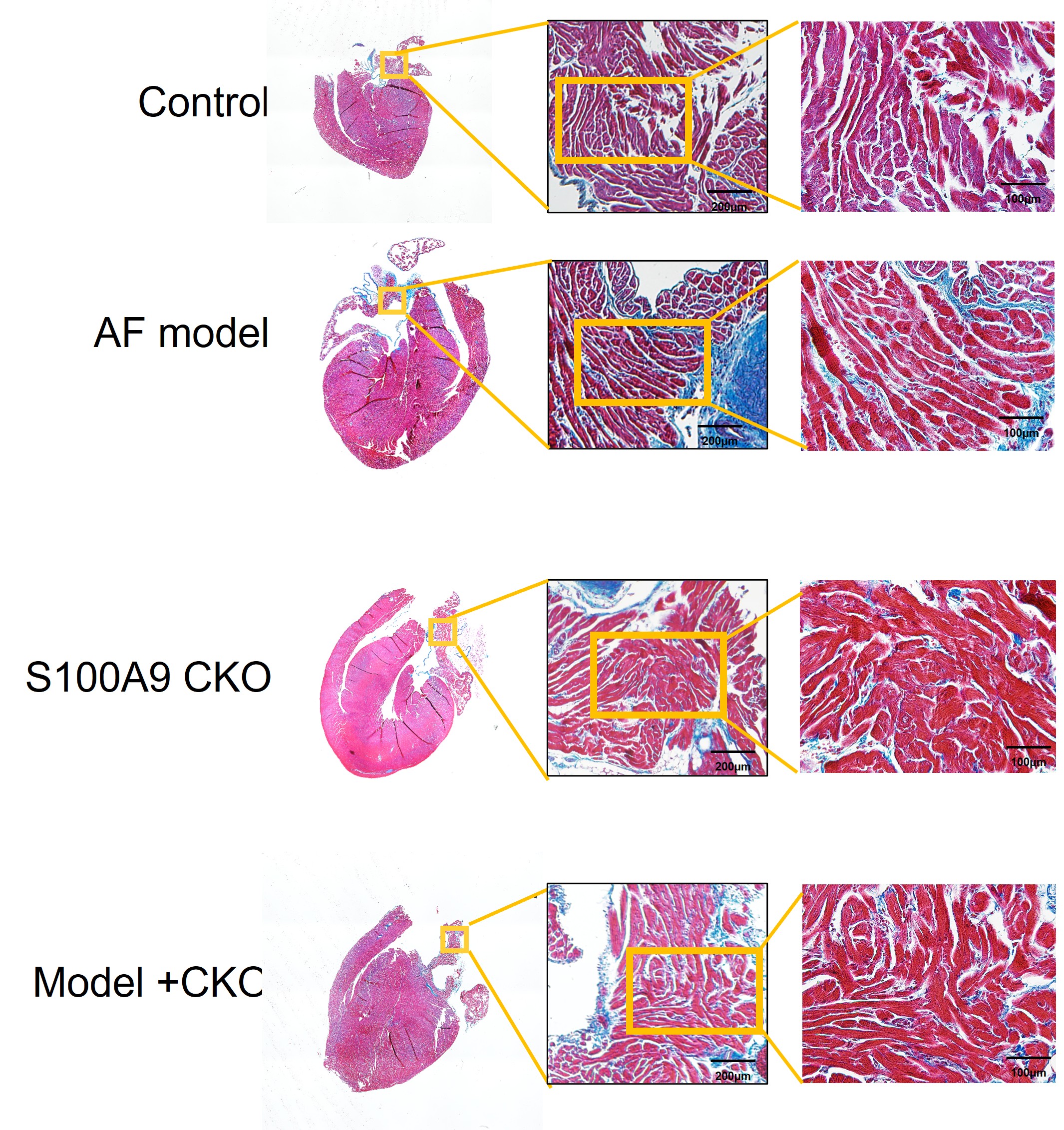

Supplement: Supplementary Figure 5 — The zoomed out photos of the atrium and whole heart stained by Masson’s trichrome staining. Scale bar 200 μm and 100 μm. [file Image5.jpeg]
