# Supplementary material for: Myeloid-specific S100A8/A9 deficiency attenuates atrial fibrillation through prevention of TLR4/NF-kB-mediated immune cell recruitment and inflammation
Source: Front Immunol. 2025 Sep 4;16:1623486. doi: 10.3389/fimmu.2025.1623486 (PMC12443547; doi:10.3389/fimmu.2025.1623486)
Supplement: Supplementary file 6 [file DataSheet1.pdf]

Supplementary file 1: The basic information of three microarray gene expression datasets and one scRNA seq dataset in the GEO

Dataset ID	Year and Country	Contributor	Number of samples	Age (years, mean ± SEM)	Male (%)	Type	GPL	Sample source
GSE41177	2013 China	Yeh et al.	19 patients underwent valvular surgery (SR 3, AF 16)	SR 53 ± 10 vs. AF 54 ± 13	10 (52.6)	Microarray	GPL570 ([HG-U133_Plus_2] Affymetrix Human Genome U133 Plus 2.0 Array)	Atrial appendage
GSE79768	2016 China	Tsai et al.	13 patients receiving surgery for mitral valve or coronary artery disease (SR 6, AF 7)	SR 64 ± 15 vs. AF 48 ±11	5 (38.5)	Microarray	GPL570 ([HG-U133_Plus_2] Affymetrix Human Genome U133 Plus 2.0 Array)	Atrial appendage

GSE115574	2019 Turkey	Deniz et al.	30 patients receiving surgery for mitral valve (SR 15, AF 15)	/	/	Microarray	GPL570 ([HG-U133_Plus_2] Affymetrix Human Genome U133 Plus 2.0 Array)	Atrial appendage
GSE224959	2023 USA	Hulsmans et al.	7 mitral regurgitation & persistent AF and 5 Controls (SR 5, AF 7)	SR 61 ± 6 vs. AF 71 ±5	10 (83.3)	sc-RNA seq	GPL18573 Illumina NextSeq 500 (Homo sapiens)	Atrial appendages

Atrial fibrillation, AF; Sinus rhythm, SR; GSE, Gene Expression Omnibus Series; GPL, Gene Expression Omnibus Platform; GEO, Gene Expression Omnibus; SD, standard deviation
