# Supplementary material for: Myeloid-specific S100A8/A9 deficiency attenuates atrial fibrillation through prevention of TLR4/NF-kB-mediated immune cell recruitment and inflammation
Source: Front Immunol. 2025 Sep 4;16:1623486. doi: 10.3389/fimmu.2025.1623486 (PMC12443547; doi:10.3389/fimmu.2025.1623486)
Supplement: Supplementary file 7 [file DataSheet2.pdf]

**Supplementary file 2:** The demographic and clinical data of 14 patients

| No. | SR or AF | Age (year) | Gender | Surgery | BMI (kg/$m^2$) | Smoking | HBP | DM |
|---|---|---|---|---|---|---|---|---|
| 1 | SR | 48 | Male | CABG | 22.4 | Yes | Yes | Yes |
| 2 | SR | 40 | Female | CABG | 24.9 | No | No | No |
| 3 | SR | 47 | Male | Valve | 25.1 | No | No | No |
| 4 | SR | 41 | Male | Valve | 25.4 | No | No | No |
| 5 | SR | 42 | Male | Valve | 22.4 | No | No | Yes |
| 6 | SR | 59 | Male | CABG | 20.3 | No | No | No |
| 7 | SR | 35 | Female | Valve | 27.3 | Yes | Yes | No |
| 8 | AF | 44 | Male | Valve | 22.4 | No | No | No |
| 9 | AF | 60 | Female | CABG | 19.4 | No | No | No |
| 10 | AF | 49 | Female | Valve | 25.9 | No | No | Yes |
| 11 | AF | 44 | Male | Valve | 29.1 | No | Yes | No |
| 12 | AF | 32 | Male | CABG | 25 | No | No | Yes |
| 13 | AF | 40 | Female | CABG | 27 | Yes | Yes | No |
| 14 | AF | 55 | Male | CABG | 26.5 | No | No | Yes |

AF, Atrial fibrillation; SR, Sinus rhythm; F, Female; M, Male; BMI, Body mass index; HBP, High blood pressure; DM, Diabetes mellitus; CABG, Coronary artery bypass surgery.
