# Supplementary material for: Myeloid-specific S100A8/A9 deficiency attenuates atrial fibrillation through prevention of TLR4/NF-kB-mediated immune cell recruitment and inflammation
Source: Front Immunol. 2025 Sep 4;16:1623486. doi: 10.3389/fimmu.2025.1623486 (PMC12443547; doi:10.3389/fimmu.2025.1623486)
Supplement: Supplementary file 8 [file DataSheet3.pdf]

Supplementary file 3: Primer sequence for RT-PCR

mRNA	Forwards	Rewards
S100A8	GGAGTTCCTTGCGATGGTGA	GGCCAGAAGCTCTGCTACTC
S100A9	ATACTCTAGGAAGGAAGGACACC	TCCATGATGTCATTTATGAGGGC
IL-1β	TGGTGTGTGACGTTCCCATT	TCGTTGCTTGGTTCTCCTTG
IL-6	CTTCCATCCAGTTGCCTTCTTG	AATTAAGCCTCCGACTTGTGAAG
TNF-α	AGGGCTGTGGGACCTAAATGT	ATGGGATGAGTATGGGGCAGC
NOX1	CCCATCCAGTCTCCAAACATGAC	ACCAAAGCTACAGTGGCAATCAC
NOX2	GGGAACTGGGCTGTGAATGA	CAATTGTGTGGATGGCGGTG
GAPDH	CATCACTGCCACCCAGAAGACTG	ATGCCAGTGAGCTTCCCGTTCAG
