# Supplementary material for: Myeloid-specific S100A8/A9 deficiency attenuates atrial fibrillation through prevention of TLR4/NF-kB-mediated immune cell recruitment and inflammation
Source: Front Immunol. 2025 Sep 4;16:1623486. doi: 10.3389/fimmu.2025.1623486 (PMC12443547; doi:10.3389/fimmu.2025.1623486)
Supplement: Supplementary file 9 [file DataSheet4.pdf]

| row.names | logFC | AveExpr | t | P.Value | adj.P.Val | B | change |
|---|---|---|---|---|---|---|---|
| 213 | 1.73456088 | 12.023631 | 3.72808685 | 0.00043146 | 0.04210976 | -0.0810861 | up |
| 51 | 1.50442726 | 4.08177721 | 4.76434831 | 1.2549E-05 | 0.00500296 | 3.10591001 | up |
| 42 | 1.40718473 | 5.88811843 | 4.87604595 | 8.3914E-06 | 0.0041625 | 3.47059219 | up |
| 141 | 1.35719277 | 8.79879283 | 3.96791952 | 0.00019724 | 0.02881514 | 0.61999489 | up |
| 89 | 1.30925235 | 4.31715517 | 4.34672515 | 5.4675E-05 | 0.01273285 | 1.77500507 | up |
| 16 | 1.24290823 | 7.83769218 | 5.52806809 | 7.5374E-07 | 0.00098492 | 5.65746681 | up |
| 6 | 1.22576882 | 7.41834463 | 6.34588292 | 3.2993E-08 | 0.00011086 | 8.49649934 | up |
| 157 | 1.09408428 | 5.88951093 | 3.87913655 | 0.00026428 | 0.03450336 | 0.35754503 | up |
| 3 | 1.04497275 | 7.52875786 | 6.56515234 | 1.4078E-08 | 8.2784E-05 | 9.2679723 | up |
| 108 | 0.92980115 | 7.01912441 | 4.19911657 | 9.0715E-05 | 0.01748948 | 1.31847268 | up |
| 177 | 0.9257671 | 5.66868134 | 3.81594337 | 0.00032482 | 0.03801074 | 0.17280494 | up |
| 43 | 0.91366826 | 8.3224873 | 4.86926814 | 8.5997E-06 | 0.0041625 | 3.44836846 | up |
| 9 | 0.87750522 | 7.37213112 | 6.09823106 | 8.5871E-08 | 0.00020198 | 7.62915767 | up |
| 127 | 0.86573735 | 5.07252913 | 4.08408146 | 0.00013386 | 0.02154083 | 0.96830176 | up |
| 35 | 0.82342354 | 6.9055944 | 5.01654331 | 5.0342E-06 | 0.00303612 | 3.93389158 | up |
| 111 | 0.78486008 | 4.8330257 | 4.17576919 | 9.8208E-05 | 0.01833294 | 1.24699374 | up |
| 11 | 0.77295959 | 9.52160207 | 5.96784335 | 1.417E-07 | 0.00027774 | 7.1747409 | up |
| 64 | 0.75908408 | 6.44756788 | 4.59636688 | 2.2833E-05 | 0.00716074 | 2.56407323 | up |
| 232 | 0.74378913 | 7.74091711 | 3.67707624 | 0.00050799 | 0.04577916 | -0.2268794 | up |
| 82 | 0.74094802 | 3.87236763 | 4.39717147 | 4.5907E-05 | 0.01139179 | 1.93279422 | up |
| 99 | 0.73587773 | 6.79060249 | 4.2827422 | 6.8159E-05 | 0.01418723 | 1.57615143 | up |
| 44 | 0.69922545 | 7.57162244 | 4.86696888 | 8.6715E-06 | 0.0041625 | 3.44083215 | up |
| 72 | 0.66908775 | 7.09356674 | 4.47297496 | 3.5246E-05 | 0.00995013 | 2.17151312 | up |
| 85 | 0.66681861 | 6.75995565 | 4.3815464 | 4.8466E-05 | 0.01175215 | 1.88382786 | up |
| 252 | 0.66430536 | 6.74031166 | 3.62468177 | 0.0006 | 0.04994295 | -0.3753573 | up |
| 128 | 0.61908297 | 3.51537867 | 4.08283099 | 0.00013442 | 0.02154083 | 0.96452347 | up |
| 76 | 0.61709055 | 5.19605405 | 4.46060867 | 3.6804E-05 | 0.00995013 | 2.13243952 | up |
| 118 | 0.60839273 | 6.24398305 | 4.11453634 | 0.00012081 | 0.02085357 | 1.06051099 | up |
| 122 | 0.60752494 | 5.22783298 | 4.10432502 | 0.00012504 | 0.02085357 | 1.02955342 | up |
| 153 | 0.60604166 | 4.21728346 | 3.89416768 | 0.00025157 | 0.03360165 | 0.4017427 | up |
| 215 | 0.56858563 | 4.31531998 | 3.7206177 | 0.00044193 | 0.04260541 | -0.102509 | up |
| 38 | 0.56809104 | 6.5665494 | 4.9489247 | 6.4417E-06 | 0.00352363 | 3.71030014 | up |
| 136 | 0.56427946 | 7.85587542 | 4.0187588 | 0.00016657 | 0.02544077 | 0.7717618 | up |
| 19 | 0.56392281 | 4.32181344 | 5.40877994 | 1.1794E-06 | 0.0012602 | 5.25096999 | up |
| 150 | 0.54162843 | 4.74822461 | 3.91764542 | 0.00023288 | 0.03184567 | 0.47097038 | up |
| 50 | 0.51328808 | 8.1387355 | 4.79282925 | 1.1329E-05 | 0.00473616 | 3.19857672 | up |
| 67 | 0.50751697 | 4.33295408 | 4.52524318 | 2.9343E-05 | 0.00873925 | 2.33721159 | up |
| 125 | 0.50560109 | 5.80931371 | 4.09890338 | 0.00012735 | 0.02085357 | 1.01313324 | up |

| SYMBOL | ENTREZID |
|---|---|
| NPPB | 4879 |
| ATP1B4 | 23439 |
| COLQ | 8292 |
| IGFBP2 | 3485 |
| COMP | 1311 |
| SLC6A6 | 6533 |
| CHGB | 1114 |
| LINC00702 | 100652988 |
| ANGPTL2 | 23452 |
| RPL3L | 6123 |
| DPYSL4 | 10570 |
| DHRS9 | 10170 |
| RCAN1 | 1827 |
| UNC5B-AS1 | 728978 |
| KCNK3 | 3777 |
| MTCL1 | 23255 |
| DNAJA4 | 55466 |
| RNF216 | 54476 |
| PDE8B | 8622 |
| DGKI | 9162 |
| LBH | 81606 |
| EXT1 | 2131 |
| RASL11B | 65997 |
| EHD3 | 30845 |
| ETV5 | 2119 |
| SRPX2 | 27286 |
| LINC00339 | 29092 |
| VASH1 | 22846 |
| KDELR3 | 11015 |
| CYP26B1 | 56603 |
| SOX9-AS1 | 400618 |
| VPS8 | 23355 |
| KIFAP3 | 22920 |
| CTNND2 | 1501 |
| P2RX5 | 5026 |
| HEXB | 3074 |
| GALNT10 | 55568 |
| XYLT1 | 64131 |
