# Supplementary material for: Myeloid-specific S100A8/A9 deficiency attenuates atrial fibrillation through prevention of TLR4/NF-kB-mediated immune cell recruitment and inflammation
Source: Front Immunol. 2025 Sep 4;16:1623486. doi: 10.3389/fimmu.2025.1623486 (PMC12443547; doi:10.3389/fimmu.2025.1623486)
Supplement: Supplementary file 10 [file DataSheet5.pdf]

row.names	SYMBOL	Score	Freq
TRDN	TRDN	9.7894E-06	3
C1orf105	C1orf105	2.1507E-05	3
S100A12	S100A12	2.8478E-05	2
CHGB	CHGB	0.00011378	2
CXCR2	CXCR2	0.00020211	2
COMP	COMP	0.00031555	2
DNER	DNER	0.00038166	2
MNDA	MNDA	0.00053264	2
DHRS9	DHRS9	0.00070857	2
AQP4	AQP4	0.00085686	2
S100A8	S100A8	0.00101912	2
GPR22	GPR22	0.00107631	2
OTOGL	OTOGL	0.00113505	2
CLC	CLC	0.00180745	2
SLC26A9	SLC26A9	0.00187078	3
LBH	LBH	0.00196042	2
PSPH	PSPH	0.00266904	1
UPK1B	UPK1B	0.00266904	1
NPPB	NPPB	0.00266904	1
MYO5B	MYO5B	0.00291148	2
CHL1	CHL1	0.00371926	2
RGS18	RGS18	0.00371926	2
BEX2	BEX2	0.00382707	2
MSLN	MSLN	0.00533649	1
MCOLN3	MCOLN3	0.00533649	1
CAPN6	CAPN6	0.00562601	2
RCAN1	RCAN1	0.00622659	3
KCNJ3	KCNJ3	0.00701351	2
IGHM	IGHM	0.00800237	1
ATP1B4	ATP1B4	0.00800237	1
BCHE	BCHE	0.00839008	2
FCGR3B	FCGR3B	0.01066666	1
PRR9	PRR9	0.01066666	1
COLQ	COLQ	0.01066666	2
SLC6A6	SLC6A6	0.01077046	2
SOSTDC1	SOSTDC1	0.01226047	2
S100A9	S100A9	0.01332936	1
IGFBP2	IGFBP2	0.01332936	2
CXCL1	CXCL1	0.01332936	1
EVI2B	EVI2B	0.01364119	2
RORB	RORB	0.01425483	2
CPLX3	CPLX3	0.01599049	1
NCF2	NCF2	0.01821101	2
KLK11	KLK11	0.01865004	1
BCL2A1	BCL2A1	0.021308	1
FAM110C	FAM110C	0.021308	2
TNNI1	TNNI1	0.021308	2
FAM216B	FAM216B	0.02396438	1
AKAP3	AKAP3	0.02396438	1

TYROBP	TYROBP	0.02661919	1
AADAC	AADAC	0.02661919	1
SLC39A8	SLC39A8	0.02924611	2
PEX1	PEX1	0.02927241	1
LINC00844	LINC00844	0.02927241	2
RNF128	RNF128	0.03192406	1
B3GALT2	B3GALT2	0.03192406	1
NMU	NMU	0.03457413	1
LINC01133	LINC01133	0.03457413	1
LRRN3	LRRN3	0.03457413	1
FAM83B	FAM83B	0.03722262	1
BNC1	BNC1	0.03722262	1
GPR34	GPR34	0.03965229	2
NEB	NEB	0.03986953	1
TCEAL2	TCEAL2	0.03986953	2
DPYSL4	DPYSL4	0.04033121	2
CFD	CFD	0.04251486	1
LINC00702	LINC00702	0.04251486	1
MPEG1	MPEG1	0.04310151	2
SOD2	SOD2	0.04515862	1
SLC7A11	SLC7A11	0.04515862	2
ACTA1	ACTA1	0.04515862	1
ITGB8	ITGB8	0.04705264	2
SLC35G3	SLC35G3	0.04780081	1
ANGPTL2	ANGPTL2	0.04780081	1
KLHL41	KLHL41	0.04927668	2
CPNE4	CPNE4	0.05044141	1
GALNT16	GALNT16	0.05044141	3
MEDAG	MEDAG	0.05044141	1
PDPN	PDPN	0.05308044	1
LGR6	LGR6	0.05308044	1
SUSD4	SUSD4	0.05465383	2
CNTN3	CNTN3	0.0557179	1
PROK2	PROK2	0.05835379	2
LRRC49	LRRC49	0.06070433	2
NRAP	NRAP	0.0609881	1
PPP1R1A	PPP1R1A	0.0609881	1
HLA-DPB1	HLA-DPB1	0.066252	1
PKIB	PKIB	0.066252	1
SFRP5	SFRP5	0.066252	1
FAM81B	FAM81B	0.06888159	1
RPL3L	RPL3L	0.06888159	1
ZDHHC20	ZDHHC20	0.07150961	1
LRRN4	LRRN4	0.07150961	1
VNN2	VNN2	0.07413606	1
IL1B	IL1B	0.07413606	1
TSC22D3	TSC22D3	0.07676093	1
CEP83	CEP83	0.07676093	1
FAM181B	FAM181B	0.07676093	1
PPIB	PPIB	0.07938424	1

COG5	COG5	0.07938424	2
CLGN	CLGN	0.08200598	1
TLL2	TLL2	0.08200598	2
C21orf62	C21orf62	0.08200598	1
SYT13	SYT13	0.08443303	2
SERPINA1	SERPINA1	0.08462615	1
ASTN2	ASTN2	0.08462615	2
PHACTR3	PHACTR3	0.08462615	1
CMTM2	CMTM2	0.08588385	2
S100A4	S100A4	0.08724475	1
PPP1R1C	PPP1R1C	0.08724475	1
CD37	CD37	0.08980824	2
EFNB3	EFNB3	0.08986178	1
GNB2	GNB2	0.09247724	1
KRT18	KRT18	0.09247724	1
UNC5B-AS1	UNC5B-AS1	0.09247724	1
C1QA	C1QA	0.09509114	1
CEL	CEL	0.09509114	2
GPRC5A	GPRC5A	0.09770346	1
ID1	ID1	0.09944995	2
RGS13	RGS13	0.10031422	1
RPL36	RPL36	0.10292342	1
FRAS1	FRAS1	0.10292342	1
KCNK3	KCNK3	0.10292342	1
MGP	MGP	0.10553105	1
FBN2	FBN2	0.10553105	1
GADD45G	GADD45G	0.10553105	1
P2RY13	P2RY13	0.10684074	2
LYVE1	LYVE1	0.10813711	1
CNN3	CNN3	0.10813711	1
CACNB2	CACNB2	0.10813711	1
C1QB	C1QB	0.11074161	1
C3	C3	0.11074161	1
ANK3	ANK3	0.11334455	1
PLLP	PLLP	0.11334455	1
DNAJA4	DNAJA4	0.11334455	1
ATP8B4	ATP8B4	0.1133669	2
C1QC	C1QC	0.11594592	1
MYBPC1	MYBPC1	0.11594592	1
RPS11	RPS11	0.11781488	2
RNF216	RNF216	0.11854573	1
RASD1	RASD1	0.12114397	1
ZNF208	ZNF208	0.12114397	1
PDE8B	PDE8B	0.12114397	1
SELL	SELL	0.12374066	1
MAB21L2	MAB21L2	0.12374066	1
DGKI	DGKI	0.12374066	1
MAL2	MAL2	0.12633578	1
PPM1L	PPM1L	0.12633578	2
SLC3A1	SLC3A1	0.12892934	1

WT1	WT1	0.12892934	1
RPS21	RPS21	0.13152134	1
SLC27A6	SLC27A6	0.13411177	1
TRA2A	TRA2A	0.13411177	1
ANKRD23	ANKRD23	0.13411177	1
STXBP6	STXBP6	0.13670065	2
FGF7	FGF7	0.13670065	1
COX16	COX16	0.13757418	2
LRRC7	LRRC7	0.13928797	1
RSAD2	RSAD2	0.13928797	1
TMEM178A	TMEM178A	0.13928797	1
FXYD1	FXYD1	0.14187373	1
SNX22	SNX22	0.14187373	2
ASB11	ASB11	0.14187373	1
SHB	SHB	0.14425931	2
ACTA2	ACTA2	0.14445793	1
GFPT2	GFPT2	0.14445793	1
RASGRP2	RASGRP2	0.14445793	1
ETNPPL	ETNPPL	0.14704057	1
GPR160	GPR160	0.14704057	1
MPP3	MPP3	0.14704057	1
C5AR1	C5AR1	0.14962166	1
EXT1	EXT1	0.14962166	1
CST3	CST3	0.15220119	1
EPHB6	EPHB6	0.15220119	1
SLC1A3	SLC1A3	0.15220119	1
LRIF1	LRIF1	0.15477916	1
ULK4	ULK4	0.15477916	1
IGFBP6	IGFBP6	0.15735557	1
DLL1	DLL1	0.15735557	1
RASL11B	RASL11B	0.15993043	1
MRPS6	MRPS6	0.16250373	1
EHD3	EHD3	0.16250373	1
BMPR1B	BMPR1B	0.16385285	2
MRPL54	MRPL54	0.16507548	1
EPHA3	EPHA3	0.16507548	1
ETV5	ETV5	0.16507548	1
DPYSL2	DPYSL2	0.16764568	1
LRRC39	LRRC39	0.16764568	1
NTRK2	NTRK2	0.16764568	1
CD300LG	CD300LG	0.17021432	1
CACNA1G	CACNA1G	0.17021432	1
CYBA	CYBA	0.1727814	1
IL18	IL18	0.1727814	1
CYBB	CYBB	0.17534694	1
CBLN2	CBLN2	0.17534694	1
BMP7	BMP7	0.17534694	1
BRINP3	BRINP3	0.17791092	1
GNAO1	GNAO1	0.17791092	2
IER3	IER3	0.17791092	1

GAPT	GAPT	0.18047335	2
USP32P2	USP32P2	0.18303423	1
RPA2	RPA2	0.18559355	1
AOX1	AOX1	0.18559355	1
KLF10	KLF10	0.18559355	1
NAP1L2	NAP1L2	0.18815133	1
SRPX2	SRPX2	0.18815133	2
UTS2	UTS2	0.18815133	1
SOD1	SOD1	0.19070756	1
PPBP	PPBP	0.19070756	1
LINC00339	LINC00339	0.19070756	1
LY96	LY96	0.19326223	1
WWC1	WWC1	0.19326223	1
RGS6	RGS6	0.19326223	1
NPC2	NPC2	0.19581536	1
PRR15	PRR15	0.19581536	1
FCN1	FCN1	0.19836694	1
PKHD1L1	PKHD1L1	0.19836694	1
VASH1	VASH1	0.19836694	1
KLF2	KLF2	0.20091697	1
PPP1R3A	PPP1R3A	0.20091697	1
KDELR3	KDELR3	0.20091697	1
RERGL	RERGL	0.20346545	1
CYP26B1	CYP26B1	0.20346545	1
TSPAN15	TSPAN15	0.20601238	1
HOTTIP	HOTTIP	0.20601238	1
FAT3	FAT3	0.20601238	1
ECHS1	ECHS1	0.20855777	1
MDM1	MDM1	0.20855777	2
SLC16A1	SLC16A1	0.20855777	1
VWDE	VWDE	0.21110161	1
PRMT1	PRMT1	0.21364391	1
LINC00520	LINC00520	0.21364391	1
TESC	TESC	0.21364391	1
LIN7A	LIN7A	0.21618466	1
C16orf54	C16orf54	0.21618466	1
SOX9-AS1	SOX9-AS1	0.21618466	1
ALOX5AP	ALOX5AP	0.21872386	1
TOMM22	TOMM22	0.21872386	2
VPS8	VPS8	0.21872386	1
CARNS1	CARNS1	0.22126152	1
KIFAP3	KIFAP3	0.22126152	1
NUDT12	NUDT12	0.22379764	1
TFRC	TFRC	0.22379764	1
CTNND2	CTNND2	0.22379764	1
PDE3A	PDE3A	0.22633221	1
F2RL2	F2RL2	0.22633221	1
HLF	HLF	0.22633221	1
XK	XK	0.22886524	1
CRISPLD1	CRISPLD1	0.22886524	1

NMRK1	NMRK1	0.22886524	1
NET1	NET1	0.23139673	1
BCL6	BCL6	0.23139673	1
ISOC2	ISOC2	0.23392668	1
CST6	CST6	0.23392668	1
ERBB4	ERBB4	0.23645508	1
SH3KBP1	SH3KBP1	0.23645508	1
P2RX5	P2RX5	0.23898194	1
SLA	SLA	0.24150726	1
RALYL	RALYL	0.24150726	2
PTPRG	PTPRG	0.24150726	1
CLPTM1L	CLPTM1L	0.24403104	1
ALDH1A1	ALDH1A1	0.24403104	1
VIT	VIT	0.24403104	1
SNORA72	SNORA72	0.24655328	1
NSG1	NSG1	0.24655328	1
CSF1R	CSF1R	0.24907399	1
EIF4E	EIF4E	0.24907399	1
HEXB	HEXB	0.24907399	1
CXCL17	CXCL17	0.25159315	1
COL21A1	COL21A1	0.25159315	1
RALGPS2	RALGPS2	0.25159315	1
TUFT1	TUFT1	0.25285232	2
CRIP1	CRIP1	0.25411077	1
BDKRB1	BDKRB1	0.25411077	1
GALNT10	GALNT10	0.25411077	1
PAQR5	PAQR5	0.25520673	2
RGS1	RGS1	0.25662686	1
UBE2M	UBE2M	0.25662686	2
XYLT1	XYLT1	0.25662686	1
FTCDNL1	FTCDNL1	0.25914141	1
NRP1	NRP1	0.25914141	1
ARF5	ARF5	0.26165442	1
ZBTB6	ZBTB6	0.26165442	1
HOOK1	HOOK1	0.2641659	1
P4HA1	P4HA1	0.2641659	2
EIF3G	EIF3G	0.26667584	1
P2RY14	P2RY14	0.26667584	1
CSF2RB	CSF2RB	0.26872027	2
ITGB2	ITGB2	0.26918424	1
GPR171	GPR171	0.27169111	1
MCTP2	MCTP2	0.27169111	1
DDIT4	DDIT4	0.27419644	1
IDH3A	IDH3A	0.27419644	1
ZNF124	ZNF124	0.27670024	1
INPP4B	INPP4B	0.27670024	1
LINC01018	LINC01018	0.27920251	1
SLCO4C1	SLCO4C1	0.28170324	1
CXADR	CXADR	0.28170324	1
TLR8	TLR8	0.28252265	2

NT5DC4	NT5DC4	0.28420244	1
PKP2	PKP2	0.28420244	1
IGFBP3	IGFBP3	0.28670011	1
GPM6A	GPM6A	0.28670011	1
ZNF682	ZNF682	0.28919625	1
SLC16A9	SLC16A9	0.28919625	1
MGC70870	MGC70870	0.29169085	1
FGFR2	FGFR2	0.29169085	1
EPHX1	EPHX1	0.29418392	1
FGFR1OP2	FGFR1OP2	0.29418392	1
EEF1A2	EEF1A2	0.29667547	1
FRZB	FRZB	0.29667547	1
FIS1	FIS1	0.29916548	1
CENPQ	CENPQ	0.29916548	1
RAC2	RAC2	0.30165396	1
MYC	MYC	0.30414092	1
DRAP1	DRAP1	0.30662634	1
FPGT	FPGT	0.30662634	1
IFI6	IFI6	0.30911024	1
FLI1	FLI1	0.30911024	1
MIF	MIF	0.31159261	1
OTUD6B	OTUD6B	0.31407346	1
DMKN	DMKN	0.31407346	2
MGAM	MGAM	0.31655277	1
PROCR	PROCR	0.31655277	1
RILP	RILP	0.31903056	1
ARRDC3	ARRDC3	0.31903056	1
RPL37A	RPL37A	0.32150683	1
NPY1R	NPY1R	0.32150683	1
SHROOM2	SHROOM2	0.32398157	1
WWC3	WWC3	0.32645478	1
TNN	TNN	0.32645478	1
LINC00622	LINC00622	0.32892647	1
SMTNL2	SMTNL2	0.32892647	2
NAIP	NAIP	0.33139664	1
TMA16	TMA16	0.33139664	1
FBLN5	FBLN5	0.33386528	1
DHCR24	DHCR24	0.33386528	1
PLTP	PLTP	0.3363324	1
COL15A1	COL15A1	0.3363324	1
FCGR2A	FCGR2A	0.338798	1
CCL23	CCL23	0.34126208	1
RABAC1	RABAC1	0.34372463	1
PDE4DIP	PDE4DIP	0.34372463	1
NCKAP5	NCKAP5	0.34618566	1
WNT2B	WNT2B	0.34618566	1
TRPM7	TRPM7	0.34864518	1
FCER1G	FCER1G	0.35110317	1
RBBP4	RBBP4	0.35110317	1
BANF1	BANF1	0.35355964	1

CALCRL	CALCRL	0.35355964	1
PGAM5	PGAM5	0.3560146	1
CD200	CD200	0.3560146	1
MIR302B	MIR302B	0.35846803	1
CBLN1	CBLN1	0.35846803	1
RPS3	RPS3	0.36091995	1
HCG11	HCG11	0.36091995	1
SHISA3	SHISA3	0.36337035	1
BNC2	BNC2	0.36337035	2
SLC16A7	SLC16A7	0.36581923	1
BCR	BCR	0.36581923	1
CADM2	CADM2	0.36826659	1
CPA3	CPA3	0.37071244	1
TMEM176A	TMEM176A	0.37071244	1
GRIN2A	GRIN2A	0.37315677	1
NELL2	NELL2	0.37315677	1
DNM3OS	DNM3OS	0.37559959	1
CD52	CD52	0.37804089	1
TM6SF1	TM6SF1	0.37804089	1
HIGD2A	HIGD2A	0.38048068	1
CMA1	CMA1	0.38048068	1
NKAIN1	NKAIN1	0.38291896	1
PHGDH	PHGDH	0.38291896	1
CHIC1	CHIC1	0.38535572	1
ANO1	ANO1	0.38535572	1
CD81	CD81	0.38779097	1
PRKD3	PRKD3	0.38779097	1
CHCHD2	CHCHD2	0.3902247	1
CTSZ	CTSZ	0.3902247	1
SPI1	SPI1	0.39508763	1
IGDCC4	IGDCC4	0.39508763	1
PARD6B	PARD6B	0.39751683	1
RNF130	RNF130	0.39994452	1
DOCK10	DOCK10	0.39994452	1
EPCAM	EPCAM	0.4023707	1
CDKN2AIPNL	CDKN2AIPNL	0.4023707	1
ARSJ	ARSJ	0.40479537	1
NCEH1	NCEH1	0.40479537	1
NFKBIA	NFKBIA	0.40721853	1
LPAR6	LPAR6	0.40721853	1
CEBPD	CEBPD	0.40964018	1
ZNF506	ZNF506	0.40964018	1
ND6	ND6	0.41206033	1
DNAJB4	DNAJB4	0.41206033	1
ABCD2	ABCD2	0.41447896	1
ACSL4	ACSL4	0.41689609	1
HSPA4L	HSPA4L	0.41689609	1
VWC2	VWC2	0.41931171	1
SLC14A1	SLC14A1	0.41931171	1
CD74	CD74	0.42172582	1

NRN1	NRN1	0.42172582	1
OR2F2	OR2F2	0.42413843	1
ZCCHC7	ZCCHC7	0.42413843	1
NNT-AS1	NNT-AS1	0.42654954	1
PPFIBP1	PPFIBP1	0.42654954	1
ABCB11	ABCB11	0.42895913	1
HLA-DRA	HLA-DRA	0.42895913	2
LINC00326	LINC00326	0.43136723	1
CNTN4	CNTN4	0.43136723	1
FOLR2	FOLR2	0.43377381	1
CCDC121	CCDC121	0.43377381	1
DAPL1	DAPL1	0.4361789	1
LRRK2	LRRK2	0.4361789	1
KANK1	KANK1	0.43858248	1
PID1	PID1	0.44098456	1
CCDC141	CCDC141	0.44098456	1
ZDHHC13	ZDHHC13	0.44338514	1
GLRB	GLRB	0.44578421	1
SLC41A2	SLC41A2	0.44578421	1
VAMP8	VAMP8	0.44818179	1
RHOU	RHOU	0.44818179	1
TMEM171	TMEM171	0.45536551	1
PIBF1	PIBF1	0.45536551	1
ANXA6	ANXA6	0.45775708	1
CDON	CDON	0.45775708	2
PDIA3	PDIA3	0.46014716	1
CLDN15	CLDN15	0.46014716	1
ATP1B3	ATP1B3	0.46253573	1
LSM12	LSM12	0.46253573	1
COL4A6	COL4A6	0.46492281	1
SNAI2	SNAI2	0.46492281	1
GUK1	GUK1	0.46730839	1
CRTAM	CRTAM	0.46969247	1
EIF4E3	EIF4E3	0.46969247	1
KIF21A	KIF21A	0.47207506	1
SCN7A	SCN7A	0.47207506	1
IGSF5	IGSF5	0.47445615	1
LMOD3	LMOD3	0.47445615	1
CCNB3	CCNB3	0.47683575	1
OAS2	OAS2	0.47683575	1
PCDHB9	PCDHB9	0.47921385	1
IRF2	IRF2	0.47921385	1
ARHGAP5	ARHGAP5	0.48159045	1
DNAAF2	DNAAF2	0.48159045	1
ZNF92	ZNF92	0.48396556	1
CLEC4A	CLEC4A	0.48396556	2
LINC01094	LINC01094	0.48633918	1
SPX	SPX	0.48633918	1
DPYSL3	DPYSL3	0.4887113	1
SLC22A4	SLC22A4	0.4887113	1

PDK4	PDK4	0.49108194	1
MIR100HG	MIR100HG	0.49108194	1
CRLF1	CRLF1	0.49345107	1
NECAP1	NECAP1	0.49581872	1
USP28	USP28	0.49581872	1
WIF1	WIF1	0.49818488	1
RGCC	RGCC	0.50054954	1
LMBR1	LMBR1	0.50054954	1
NXPE4	NXPE4	0.50291272	1
ADAMTS15	ADAMTS15	0.50291272	1
MSRB2	MSRB2	0.5052744	1
CSDC2	CSDC2	0.5052744	1
SAMD12	SAMD12	0.50763459	1
APOBEC2	APOBEC2	0.50763459	1
YWHAQ	YWHAQ	0.5099933	1
C15orf40	C15orf40	0.5099933	1
SOX9	SOX9	0.51235052	1
CENPH	CENPH	0.51235052	1
C18orf54	C18orf54	0.51470624	1
TOPORS	TOPORS	0.51470624	1
FAM169A	FAM169A	0.51706048	1
SWAP70	SWAP70	0.51706048	1
OOSP2	OOSP2	0.51941324	1
PAPSS2	PAPSS2	0.51941324	2
METTL21A	METTL21A	0.52176451	1
TFEC	TFEC	0.52176451	1
NDUFV1	NDUFV1	0.52411429	1
DACH1	DACH1	0.52411429	1
ATP8A1	ATP8A1	0.52646258	1
ATP6V1C1	ATP6V1C1	0.52646258	1
DNAJC16	DNAJC16	0.52880939	1
NAPSB	NAPSB	0.52880939	1
EPB41L2	EPB41L2	0.53115472	1
CYSLTR1	CYSLTR1	0.53115472	1
SPHKAP	SPHKAP	0.53349856	1
KCTD3	KCTD3	0.53349856	1
LAPTM5	LAPTM5	0.53584091	1
CCDC102B	CCDC102B	0.53584091	1
FKBP11	FKBP11	0.53818178	1
SOX18	SOX18	0.53818178	1
HPGDS	HPGDS	0.53887578	2
CCPG1	CCPG1	0.54052117	1
KCNAB1	KCNAB1	0.54052117	1
HCN4	HCN4	0.54285908	1
GABPA	GABPA	0.54285908	1
MEIG1	MEIG1	0.54519551	1
RB1CC1	RB1CC1	0.54519551	1
MGAT4C	MGAT4C	0.54753045	1
UST	UST	0.54753045	1
RBM3	RBM3	0.54986391	1

PYCARD	PYCARD	0.5521959	1
PTGR2	PTGR2	0.5521959	1
DPY19L4	DPY19L4	0.5545264	1
PPP1R3C	PPP1R3C	0.5545264	2
NDUFA3	NDUFA3	0.55685542	1
YWHAH	YWHAH	0.55685542	2
LINC00354	LINC00354	0.55918296	1
POPDC3	POPDC3	0.55918296	1
MYSM1	MYSM1	0.56150903	1
STAM2	STAM2	0.56150903	1
DPY19L1P1	DPY19L1P1	0.56383361	1
LIF	LIF	0.56383361	1
COL6A6	COL6A6	0.56615672	1
PRKAG2	PRKAG2	0.56615672	1
FUNDC2	FUNDC2	0.56847835	1
CFI	CFI	0.56847835	1
BAG2	BAG2	0.57079851	1
CDH13	CDH13	0.57311719	1
PDE5A	PDE5A	0.57311719	2
NIPSNAP3A	NIPSNAP3A	0.57543439	1
CHCHD10	CHCHD10	0.57775011	1
WIPF3	WIPF3	0.57775011	1
NPHP1	NPHP1	0.58006436	1
ST8SIA4	ST8SIA4	0.58006436	1
TGFB1	TGFB1	0.58468844	1
PRPF39	PRPF39	0.58468844	1
SPTSSB	SPTSSB	0.58699827	1
HLA-DRB6	HLA-DRB6	0.58930663	1
MIS18BP1	MIS18BP1	0.58930663	1
RNASE1	RNASE1	0.59161351	1
TTC33	TTC33	0.59161351	1
KDM4B	KDM4B	0.59391892	1
TPSB2	TPSB2	0.59622286	1
SULT1C4	SULT1C4	0.59622286	1
TAS2R13	TAS2R13	0.59852533	1
RAB8B	RAB8B	0.59852533	1
NFATC2IP	NFATC2IP	0.60082633	1
LDLRAD3	LDLRAD3	0.60082633	1
CCL5	CCL5	0.60312586	1
FAM13C	FAM13C	0.60312586	1
NSF	NSF	0.60542391	1
COL9A1	COL9A1	0.60542391	1
SMARCE1	SMARCE1	0.6077205	1
LINC00888	LINC00888	0.6077205	1
RWDD3	RWDD3	0.61001562	1
RLN1	RLN1	0.61230927	1
USP3-AS1	USP3-AS1	0.61230927	1
CDK9	CDK9	0.61460146	1
MRPL44	MRPL44	0.61460146	1
GP5	GP5	0.61689217	1

FAM133A	FAM133A	0.61689217	2
PIK3CA	PIK3CA	0.61918142	1
MMP24	MMP24	0.6214692	1
CTDSPL2	CTDSPL2	0.6214692	1
PSMC3	PSMC3	0.62375552	1
GRAMD1C	GRAMD1C	0.62375552	2
SMIM17	SMIM17	0.62604037	1
ETS1	ETS1	0.62604037	1
HTR2B	HTR2B	0.62832375	1
DUSP10	DUSP10	0.62832375	1
TMEFF2	TMEFF2	0.63060567	1
SLC51A	SLC51A	0.63060567	2
GABRB3	GABRB3	0.63288613	1
ZNF697	ZNF697	0.63516512	1
CASD1	CASD1	0.63516512	1
MAST1	MAST1	0.63744265	1
CASP8	CASP8	0.63744265	1
BOD1	BOD1	0.63971872	1
SP3	SP3	0.63971872	1
PGAM1	PGAM1	0.64199332	1
MEGF6	MEGF6	0.64199332	1
LST1	LST1	0.64426647	1
MED30	MED30	0.64426647	1
CD163	CD163	0.64653815	1
REC8	REC8	0.64653815	1
HSPE1	HSPE1	0.64880837	1
RCBTB1	RCBTB1	0.64880837	1
CAPS2	CAPS2	0.65107713	1
SAR1B	SAR1B	0.65107713	1
VIM	VIM	0.65334443	1
MYCT1	MYCT1	0.65334443	1
RASEF	RASEF	0.65561027	1
RUFY2	RUFY2	0.65561027	1
SCN2B	SCN2B	0.65787465	1
SNX16	SNX16	0.66013757	1
MRPL19	MRPL19	0.66013757	1
LRP4	LRP4	0.66239904	1
TSPAN12	TSPAN12	0.66239904	1
NRXN1	NRXN1	0.66691759	1
NSUN6	NSUN6	0.66691759	1
CORO1A	CORO1A	0.66917469	1
COQ7	COQ7	0.66917469	1
TUBB6	TUBB6	0.67143032	1
IL33	IL33	0.67143032	1
TTN	TTN	0.67368451	1
FRMD3	FRMD3	0.67368451	1
TMEM63C	TMEM63C	0.67593723	1
PDIK1L	PDIK1L	0.67593723	1
DNAJC9-AS1	DNAJC9-AS1	0.6781885	1
IFIT5	IFIT5	0.6781885	1

NKAIN2	NKAIN2	0.68043832	1
RRAD	RRAD	0.68268668	1
ZNF608	ZNF608	0.68268668	1
VSIG4	VSIG4	0.68493359	1
S100P	S100P	0.68717905	1
FOXA3	FOXA3	0.68942306	1
MTURN	MTURN	0.68942306	1
BLZF1	BLZF1	0.69166561	1
TAF12	TAF12	0.69166561	1
CYGB	CYGB	0.69390671	1
PDIA4	PDIA4	0.69390671	1
IDH2	IDH2	0.69614636	1
PRKCI	PRKCI	0.69614636	1
GATA5	GATA5	0.69838456	1
CD48	CD48	0.70062131	1
MARCKS	MARCKS	0.70062131	1
KIRREL3	KIRREL3	0.70285661	1
ATP8B1	ATP8B1	0.70285661	1
LARP4B	LARP4B	0.70509046	1
RPL37	RPL37	0.70732286	1
TMEM255A	TMEM255A	0.70732286	1
ACKR1	ACKR1	0.70955382	1
CASP8AP2	CASP8AP2	0.70955382	1
KYNU	KYNU	0.71178332	1
SYNPO2L	SYNPO2L	0.71178332	1
PLEKHF2	PLEKHF2	0.71401138	1
UNC45B	UNC45B	0.71623799	1
APPL1	APPL1	0.71623799	1
NRSN1	NRSN1	0.71846316	1
FCHO2	FCHO2	0.71846316	1
HCG4	HCG4	0.72068688	1
PABPC5	PABPC5	0.72068688	1
MCOLN1	MCOLN1	0.72290915	1
HSPA9	HSPA9	0.72290915	1
MTPN	MTPN	0.72512998	1
M6PR	M6PR	0.72512998	1
YIPF5	YIPF5	0.72734937	1
E2F8	E2F8	0.72956731	1
FMO2	FMO2	0.72956731	1
MYL6	MYL6	0.73178381	1
PROKR2	PROKR2	0.73399886	1
FAM107B	FAM107B	0.73399886	1
SUCO	SUCO	0.73621247	1
SRRM2	SRRM2	0.73621247	1
LINC00869	LINC00869	0.73842464	1
DCAF17	DCAF17	0.73842464	1
LOC10050597	LOC10050597	0.74063537	1
CHIC2	CHIC2	0.74063537	2
FAM76A	FAM76A	0.74284466	1
ARHGAP44	ARHGAP44	0.74284466	2

GNAI2	GNAI2	0.74505251	1
PDGFA	PDGFA	0.74505251	1
CIAPIN1	CIAPIN1	0.74725891	1
PPM1A	PPM1A	0.74946388	1
MITD1	MITD1	0.74946388	1
UBR3	UBR3	0.75166741	1
CLDN11	CLDN11	0.75166741	1
SMAP2	SMAP2	0.7538695	1
RPL27A	RPL27A	0.7538695	1
CLEC10A	CLEC10A	0.75607015	1
MTHFD2L	MTHFD2L	0.75607015	1
NDFIP2	NDFIP2	0.75826936	1
PTGS1	PTGS1	0.75826936	1
PPARGC1B	PPARGC1B	0.76046713	1
ZNF780A	ZNF780A	0.76046713	1
ZNF837	ZNF837	0.76266347	1
COTL1	COTL1	0.76485837	1
PILRA	PILRA	0.76705184	1
TMEM64	TMEM64	0.76705184	1
ATE1	ATE1	0.76924387	1
FSTL3	FSTL3	0.76924387	1
ID2	ID2	0.77143446	1
KRTAP4-12	KRTAP4-12	0.77143446	1
VAX2	VAX2	0.77362362	1
NNT	NNT	0.77362362	1
PRDX4	PRDX4	0.77581135	1
ST3GAL5	ST3GAL5	0.77581135	1
ARFGEF2	ARFGEF2	0.77799764	1
STK31	STK31	0.77799764	1
LINC00963	LINC00963	0.7801825	1
RYR2	RYR2	0.78236593	1
DYRK2	DYRK2	0.78454792	1
SUDS3	SUDS3	0.78454792	1
HOXC10	HOXC10	0.78672849	1
DPY19L2	DPY19L2	0.78672849	1
CPEB3	CPEB3	0.78890762	1
DCK	DCK	0.78890762	1
HEG1	HEG1	0.79108532	1
TRAPPC13	TRAPPC13	0.79108532	1
CSRP1	CSRP1	0.79326159	1
RPS9	RPS9	0.79543643	1
CAPZA2	CAPZA2	0.79543643	1
LRRCC1	LRRCC1	0.79760984	1
FGF9	FGF9	0.79760984	1
PXDNL	PXDNL	0.79978182	1
DENND1A	DENND1A	0.80195238	1
CYP3A5	CYP3A5	0.80195238	1
CYFIP1	CYFIP1	0.8041215	1
ACTR6	ACTR6	0.8041215	1
ITK	ITK	0.8062892	1

C3orf38	C3orf38	0.8062892	1
SRP72	SRP72	0.80845547	1
RPN1	RPN1	0.80845547	2
PEX13	PEX13	0.81062032	1
ASMTL-AS1	ASMTL-AS1	0.81062032	1
FCGR2C	FCGR2C	0.81278374	1
NEURL1B	NEURL1B	0.81278374	1
ZNF667-AS1	ZNF667-AS1	0.81494573	2
TSC22D1	TSC22D1	0.8171063	1
ARHGEF5	ARHGEF5	0.8171063	1
BAALC	BAALC	0.81926544	1
PPP1R2	PPP1R2	0.81926544	1
LILRA6	LILRA6	0.82357945	1
ADAM32	ADAM32	0.82573433	1
HEY2	HEY2	0.82573433	1
GPI	GPI	0.82788777	1
CGN	CGN	0.8300398	1
FAM210A	FAM210A	0.8300398	1
NR3C2	NR3C2	0.83219041	1
ADAMTS5	ADAMTS5	0.83219041	1
MGMT	MGMT	0.83433959	1
STT3A	STT3A	0.83433959	1
CYP4F3	CYP4F3	0.83648735	1
C1QTNF2	C1QTNF2	0.83648735	1
RNASE6	RNASE6	0.83863369	1
EFEMP1	EFEMP1	0.84077861	1
AMMECR1	AMMECR1	0.84077861	1
PIP5K1B	PIP5K1B	0.84292212	1
ARG1	ARG1	0.8450642	1
VWF	VWF	0.84720486	1
NCBP1	NCBP1	0.84720486	1
ANP32B	ANP32B	0.84934411	1
LRCH1	LRCH1	0.84934411	1
GPR85	GPR85	0.85148194	1
FBXO8	FBXO8	0.85148194	1
ZNF708	ZNF708	0.85361835	1
NCOA6	NCOA6	0.85361835	1
ESRP1	ESRP1	0.85575335	1
EBF3	EBF3	0.85575335	1
SNX3	SNX3	0.85788692	1
MOB1B	MOB1B	0.85788692	1
IL1R2	IL1R2	0.86001909	1
SLFN5	SLFN5	0.86001909	1
CC2D2B	CC2D2B	0.86214983	1
TRIM2	TRIM2	0.86427917	1
PEX5L	PEX5L	0.86427917	1
KRT5	KRT5	0.86640709	1
PGLS	PGLS	0.86853359	1
ASB4	ASB4	0.87065868	1
IQGAP2	IQGAP2	0.87065868	1

MED23	MED23	0.87278236	1
PTN	PTN	0.87278236	1
STMN2	STMN2	0.87490462	1
RBM48	RBM48	0.87490462	1
ZNF207	ZNF207	0.87702548	1
ZNF441	ZNF441	0.87702548	1
CLIC5	CLIC5	0.87914492	1
ABCD3	ABCD3	0.88126295	1
TRIQK	TRIQK	0.88126295	1
KALRN	KALRN	0.88337957	1
CCDC152	CCDC152	0.88337957	1
SIRPB2	SIRPB2	0.88549478	1
ENPEP	ENPEP	0.88549478	1
ZNF549	ZNF549	0.88760858	1
GPX8	GPX8	0.88760858	1
RPPH1	RPPH1	0.88972097	1
KATNBL1	KATNBL1	0.88972097	1
GZMA	GZMA	0.89183196	1
NR5A2	NR5A2	0.89183196	1
NDUFB11	NDUFB11	0.89394153	1
PHLPP2	PHLPP2	0.89394153	2
LINC01355	LINC01355	0.8960497	1
PLIN2	PLIN2	0.8960497	1
NDUFAF3	NDUFAF3	0.89815646	1
TRIM16	TRIM16	0.89815646	1
RPS12	RPS12	0.90026181	1
PARM1	PARM1	0.90026181	1
PPIL1	PPIL1	0.90236575	1
PTER	PTER	0.90446829	1
USP16	USP16	0.90656943	1
MS4A4A	MS4A4A	0.91076748	1
PRELP	PRELP	0.9128644	1
RAC1	RAC1	0.91495992	1
LOC285422	LOC285422	0.91705404	1
KRTAP17-1	KRTAP17-1	0.91914675	1
THSD4	THSD4	0.92123806	1
EPHA7	EPHA7	0.92332797	1
ACADSB	ACADSB	0.92541647	1
TNFSF8	TNFSF8	0.92750358	1
ZBTB41	ZBTB41	0.92958928	1
ANKDD1A	ANKDD1A	0.93167359	1
KPNB1	KPNB1	0.935838	1
CPEB4	CPEB4	0.93791811	1
GAS2	GAS2	0.93999683	1
CD99	CD99	0.94207414	1
RCN3	RCN3	0.94622458	1
TBXAS1	TBXAS1	0.9482977	1
SPOP	SPOP	0.9545087	1
ARPC3	ARPC3	0.95657625	1
IER2	IER2	0.9586424	1

LOC10019098	LOC10019098	0.96070716	1
CFLAR-AS1	CFLAR-AS1	0.96277052	1
RINT1	RINT1	0.9648325	1
RBM11	RBM11	0.96689308	1
CELA2B	CELA2B	0.96895227	1
RPS14	RPS14	0.97101007	1
ZYX	ZYX	0.97306647	1
DUSP6	DUSP6	0.97512149	1
ARPP19	ARPP19	0.97717512	1
MED25	MED25	0.97922736	1
PTCD2	PTCD2	0.98127821	1
BLVRB	BLVRB	0.98332768	1
RGS2	RGS2	0.98537575	1
ARPC1B	ARPC1B	0.98742244	1
ARAP3	ARAP3	0.98946774	1
STT3B	STT3B	0.99151166	1
RPL6	RPL6	0.99355419	1
RPA3	RPA3	0.99559534	1
MRPL12	MRPL12	0.9976351	1
COX5B	COX5B	0.99967347	1
RASIP1	RASIP1	1	1
REST	REST	1	1
TUBGCP5	TUBGCP5	1	1
MT2A	MT2A	1	1
C1GALT1	C1GALT1	1	1
PLEKHH1	PLEKHH1	1	1
ZMIZ2	ZMIZ2	1	1
ECT2	ECT2	1	1
UQCRC1	UQCRC1	1	1
ETFB	ETFB	1	1
STRIP2	STRIP2	1	1
GMDS	GMDS	1	1
PIGT	PIGT	1	1
ITGAM	ITGAM	1	1
HBB	HBB	1	1
AQP3	AQP3	1	1
DCTN1	DCTN1	1	1
TMEM33	TMEM33	1	1
ACSS1	ACSS1	1	1
TAF1A	TAF1A	1	1
MICAL2	MICAL2	1	1
ACADM	ACADM	1	1
MKRN1	MKRN1	1	1
BCL2L13	BCL2L13	1	1
RAB8A	RAB8A	1	1
PHLDA3	PHLDA3	1	1
IGLL1	IGLL1	1	1
TMEM207	TMEM207	1	1
EPHA2	EPHA2	1	1
SCUBE2	SCUBE2	1	1

NDUFB10	NDUFB10	1	1
TMEM214	TMEM214	1	1
EDN3	EDN3	1	1
PAQR3	PAQR3	1	1
EIF5	EIF5	1	1
ZIC4	ZIC4	1	1
DNM1P46	DNM1P46	1	1
IGFBPL1	IGFBPL1	1	1
RNASET2	RNASET2	1	1
ATF7IP2	ATF7IP2	1	1
NBEA	NBEA	1	1
EGF	EGF	1	1
PCP4	PCP4	1	1
RNF183	RNF183	1	1
ANXA5	ANXA5	1	1
SLC25A6	SLC25A6	1	1
TDRP	TDRP	1	1
PPP1CB	PPP1CB	1	1
ARFGAP3	ARFGAP3	1	1
STK38L	STK38L	1	1
LINC00593	LINC00593	1	1
SLITRK6	SLITRK6	1	1
NCKIPSD	NCKIPSD	1	1
ZNF302	ZNF302	1	1
KIF1B	KIF1B	1	1
OST4	OST4	1	1
PQBP1	PQBP1	1	1
SLCO5A1	SLCO5A1	1	1
LILRB4	LILRB4	1	1
KLHL10	KLHL10	1	1
CNGA1	CNGA1	1	1
RNF212B	RNF212B	1	1
CCDC85A	CCDC85A	1	1
CDKN1A	CDKN1A	1	1
TPSAB1	TPSAB1	1	1
CLOCK	CLOCK	1	1
EXOC6B	EXOC6B	1	1
PDE4B	PDE4B	1	1
TREM1	TREM1	1	1
TRAPPC5	TRAPPC5	1	1
GZMH	GZMH	1	1
SNX20	SNX20	1	1
ENOSF1	ENOSF1	1	1
ZNF623	ZNF623	1	1
CSF3R	CSF3R	1	1
ALG13	ALG13	1	1
PPP3R2	PPP3R2	1	1
TMTC3	TMTC3	1	1
EDNRA	EDNRA	1	1
EIF4E2	EIF4E2	1	1

PPP1R9A	PPP1R9A	1	1
HLA-B	HLA-B	1	1
PRR12	PRR12	1	1
ROMO1	ROMO1	1	1
RPLP1	RPLP1	1	1
NUDCD2	NUDCD2	1	1
ALX3	ALX3	1	1
NDRG1	NDRG1	1	1
HCK	HCK	1	1
TOMM20	TOMM20	1	1
SFRP1	SFRP1	1	1
ZNF593	ZNF593	1	1
KBTBD8	KBTBD8	1	1
CSTB	CSTB	1	1
AGL	AGL	1	1
PRKG2	PRKG2	1	1
TPP1	TPP1	1	1
FAU	FAU	1	1
AKAP12	AKAP12	1	1
LINC01300	LINC01300	1	1
PLAC8	PLAC8	1	1
MFGE8	MFGE8	1	1
ATP6V0D2	ATP6V0D2	1	1
EIF4G3	EIF4G3	1	1
RPS27	RPS27	1	1
ADAMTS10	ADAMTS10	1	1
BEX1	BEX1	1	1
IGSF6	IGSF6	1	1
RASGRP3	RASGRP3	1	1
MAGOHB	MAGOHB	1	1
GRIK3	GRIK3	1	1
ARRDC2	ARRDC2	1	1
NLRP3	NLRP3	1	1
EWSR1	EWSR1	1	1
TAT	TAT	1	1
RAD51AP1	RAD51AP1	1	1
TCEAL7	TCEAL7	1	1
CCDC170	CCDC170	1	1
NLGN4Y	NLGN4Y	1	1
LOXL1	LOXL1	1	1
MPC2	MPC2	1	1
CA12	CA12	1	1
LINC00920	LINC00920	1	1
FOXC2	FOXC2	1	1
MRPS22	MRPS22	1	1
PTPRZ1	PTPRZ1	1	1
MAD2L2	MAD2L2	1	1
NQO2	NQO2	1	1
CASC15	CASC15	1	1
CXCL12	CXCL12	1	1

TMEM256	TMEM256	1	1
CD3D	CD3D	1	1
IBTK	IBTK	1	1
ACAD8	ACAD8	1	1
NDUFA11	NDUFA11	1	1
DMD	DMD	1	1
FBXL5	FBXL5	1	1
PFDN1	PFDN1	1	1
CLIC1	CLIC1	1	1
ALG1	ALG1	1	1
TMEM98	TMEM98	1	1
TCF21	TCF21	1	1
FBXW12	FBXW12	1	1
RGS10	RGS10	1	1
ATMIN	ATMIN	1	1
LOC284788	LOC284788	1	1
HSD17B12	HSD17B12	1	1
EGLN3	EGLN3	1	1
SYNGR2	SYNGR2	1	1
GLIPR2	GLIPR2	1	1
C1orf162	C1orf162	1	1
MRPL9	MRPL9	1	1
ZDHHC17	ZDHHC17	1	1
SIX3	SIX3	1	1
S100A6	S100A6	1	1
SIVA1	SIVA1	1	1
PGAM2	PGAM2	1	1
LRCH2	LRCH2	1	1
AEBP1	AEBP1	1	1
SACS	SACS	1	1
THAP5	THAP5	1	1
ABCA5	ABCA5	1	1
NFIL3	NFIL3	1	1
ZNF205-AS1	ZNF205-AS1	1	1
LGALS1	LGALS1	1	1
HSPA12A	HSPA12A	1	1
CDC42EP5	CDC42EP5	1	1
MZB1	MZB1	1	1
POU5F1P3	POU5F1P3	1	1
PTAR1	PTAR1	1	1
NEBL	NEBL	1	1
DNA2	DNA2	1	1
LINC00355	LINC00355	1	1
ZNF17	ZNF17	1	1
CNIH1	CNIH1	1	1
CD14	CD14	1	1
MAD2L1	MAD2L1	1	1
PPP1R14B	PPP1R14B	1	1
CITED2	CITED2	1	1
ANAPC16	ANAPC16	1	1

NSUN7	NSUN7	1	1
GSDMD	GSDMD	1	1
PTPRCAP	PTPRCAP	1	1
METTL15	METTL15	1	1
HRAS	HRAS	1	1
AVPR1A	AVPR1A	1	1
ZNF620	ZNF620	1	1
RPL24	RPL24	1	1
ALYREF	ALYREF	1	1
TAAR2	TAAR2	1	1
MROH8	MROH8	1	1
PRNP	PRNP	1	1
DIAPH3-AS1	DIAPH3-AS1	1	1
KLHL24	KLHL24	1	1
DUSP19	DUSP19	1	1
CARD6	CARD6	1	1
CD2	CD2	1	1
C19orf12	C19orf12	1	1
HAUS6	HAUS6	1	1
TLR1	TLR1	1	1
SENP7	SENP7	1	1
CBR4	CBR4	1	1
GNAQ	GNAQ	1	1
DOCK4	DOCK4	1	1
C12orf57	C12orf57	1	1
COMMD6	COMMD6	1	1
B4GALT6	B4GALT6	1	1
FPR1	FPR1	1	1
LRIG2	LRIG2	1	1
PLAC9	PLAC9	1	1
HSPA13	HSPA13	1	1
EFHD2	EFHD2	1	1
PRKACA	PRKACA	1	1
SERP1	SERP1	1	1
ADAMTSL4	ADAMTSL4	1	1
SLC6A8	SLC6A8	1	1
AP3S1	AP3S1	1	1
GPSM2	GPSM2	1	1
LOC1027237(	LOC1027237(	1	1
SPAST	SPAST	1	1
CCL4	CCL4	1	1
DPY19L2P2	DPY19L2P2	1	1
AGBL4	AGBL4	1	1
ZNF112	ZNF112	1	1
C1orf54	C1orf54	1	1
CPNE8	CPNE8	1	1
RNF2	RNF2	1	1
CAP2	CAP2	1	1
KLHL6	KLHL6	1	1
PDE4D	PDE4D	1	1

PRPS1	PRPS1	1	1
PLK2	PLK2	1	1
PKN2	PKN2	1	1
ABCB10	ABCB10	1	1
NKX3-1	NKX3-1	1	1
ILDR2	ILDR2	1	1
CRIM1	CRIM1	1	1
MRPL41	MRPL41	1	1
RANBP6	RANBP6	1	1
ERV3-2	ERV3-2	1	1
KLRC3	KLRC3	1	1
TNP1	TNP1	1	1
LOC1002872[illegible]	LOC1002872[illegible]	1	1
PSME1	PSME1	1	1
MAEL	MAEL	1	1
STAT6	STAT6	1	1
MLH3	MLH3	1	1
DNAH14	DNAH14	1	1
FMO3	FMO3	1	1
FLVCR1	FLVCR1	1	1
CTBP1	CTBP1	1	1
SNX14	SNX14	1	1
NMNAT1	NMNAT1	1	1
MAGEB3	MAGEB3	1	1
ALKBH3-AS1	ALKBH3-AS1	1	1
DSG2	DSG2	1	1
SCFD1	SCFD1	1	1
STEAP2	STEAP2	1	1
ROPN1B	ROPN1B	1	1
KDR	KDR	1	1
WDR17	WDR17	1	1
SMC3	SMC3	1	1
ORC4	ORC4	1	1
EAPP	EAPP	1	1
TOB1	TOB1	1	1
PHF10	PHF10	1	1
PXT1	PXT1	1	1
ERCC6L	ERCC6L	1	1
PLCE1	PLCE1	1	1
KRTCAP2	KRTCAP2	1	1
BMP2K	BMP2K	1	1
ZNF223	ZNF223	1	1
CCND3	CCND3	1	1
CASC2	CASC2	1	1
MOS	MOS	1	1
MS4A2	MS4A2	1	1
ARL6IP6	ARL6IP6	1	1
ZNHIT1	ZNHIT1	1	1
EIF3I	EIF3I	1	1
HPR	HPR	1	1

TNNC2	TNNC2	1	1
ANKRD18A	ANKRD18A	1	1
NENF	NENF	1	1
CXXC1	CXXC1	1	1
GRHL1	GRHL1	1	1
PLA2G7	PLA2G7	1	1
PODN	PODN	1	1
PGD	PGD	1	1
PDAP1	PDAP1	1	1
COX8A	COX8A	1	1
BCRP3	BCRP3	1	1
GXYLT2	GXYLT2	1	1
INTS6	INTS6	1	1
LPP-AS2	LPP-AS2	1	1
STK17B	STK17B	1	1
SMAP1	SMAP1	1	1
C1orf94	C1orf94	1	1
STXBP4	STXBP4	1	1
SBSN	SBSN	1	1
LOC10192844	LOC10192844	1	1
HMOX2	HMOX2	1	1
PAPPA	PAPPA	1	1
TRIM22	TRIM22	1	1
RASGEF1C	RASGEF1C	1	1
RBM41	RBM41	1	1
SLC12A2	SLC12A2	1	1
GABARAPL2	GABARAPL2	1	1
PMP2	PMP2	1	1
IGSF11	IGSF11	1	1
LRRC69	LRRC69	1	1
GLIS2	GLIS2	1	1
CTDSP1	CTDSP1	1	1
SLC37A2	SLC37A2	1	1
VWA5B1	VWA5B1	1	1
CABP4	CABP4	1	1
HMGB2	HMGB2	1	1
ANKRA2	ANKRA2	1	1
GPR39	GPR39	1	1
NLRP8	NLRP8	1	1
PRSS36	PRSS36	1	1
NEK2	NEK2	1	1
LCP1	LCP1	1	1
UBA3	UBA3	1	1
MGARP	MGARP	1	1
ARHGEF10	ARHGEF10	1	1
RPS6	RPS6	1	1
CR1	CR1	1	1
TMEM204	TMEM204	1	1
RHOQ	RHOQ	1	1
LINC00673	LINC00673	1	1

TRMT6	TRMT6	1	1
NDUFC1	NDUFC1	1	1
LOC10192915	LOC10192915	1	1
RAB31	RAB31	1	1
KCNK13	KCNK13	1	1
RNF19A	RNF19A	1	1
NHSL1	NHSL1	1	1
CHD6	CHD6	1	1
ITGB1BP1	ITGB1BP1	1	1
LOC10012903	LOC10012903	1	1
BHLHE22	BHLHE22	1	1
SMS	SMS	1	1
MYO6	MYO6	1	1
CD72	CD72	1	1
AFMID	AFMID	1	1
KTN1	KTN1	1	1
MED13L	MED13L	1	1
DNAJC10	DNAJC10	1	1
MEFV	MEFV	1	1
GMFG	GMFG	1	1
PITPNA	PITPNA	1	1
FCGR2B	FCGR2B	1	1
EGR1	EGR1	1	1
HECTD2	HECTD2	1	1
TFAP2C	TFAP2C	1	1
UEVLD	UEVLD	1	1
KIF5C	KIF5C	1	1
KDM7A	KDM7A	1	1
HIBCH	HIBCH	1	1
NFE2	NFE2	1	1
ARMC12	ARMC12	1	1
G6PD	G6PD	1	1
KIF5B	KIF5B	1	1
C1orf116	C1orf116	1	1
RRAGD	RRAGD	1	1
LGMN	LGMN	1	1
FAM162B	FAM162B	1	1
PSMA7	PSMA7	1	1
MTCH1	MTCH1	1	1
C12orf75	C12orf75	1	1
ABCA4	ABCA4	1	1
ROR2	ROR2	1	1
KCNC2	KCNC2	1	1
ETAA1	ETAA1	1	1
FRMD5	FRMD5	1	1
MYH4	MYH4	1	1
RAB32	RAB32	1	1
LINC00839	LINC00839	1	1
CPS1-IT1	CPS1-IT1	1	1
TSPAN13	TSPAN13	1	1

SRRT	SRRT	1	1
MACROD2	MACROD2	1	1
PSG3	PSG3	1	1
SLIRP	SLIRP	1	1
ODF3B	ODF3B	1	1
COL1A2	COL1A2	1	1
ARNT2	ARNT2	1	1
LYPD4	LYPD4	1	1
BROX	BROX	1	1
DYNC1I1	DYNC1I1	1	1
NKTR	NKTR	1	1
MCC	MCC	1	1
NT5C3B	NT5C3B	1	1
CARD8	CARD8	1	1
NOSIP	NOSIP	1	1
CRYAB	CRYAB	1	1
PRDX3	PRDX3	1	1
VPREB1	VPREB1	1	1
LAMTOR4	LAMTOR4	1	1
TXNL4A	TXNL4A	1	1
COLGALT2	COLGALT2	1	1
THOC2	THOC2	1	1
MPG	MPG	1	1
FBLN1	FBLN1	1	1
CDK5	CDK5	1	1
SLC4A4	SLC4A4	1	1
OMA1	OMA1	1	1
FBXO32	FBXO32	1	1
IRS1	IRS1	1	1
SPIDR	SPIDR	1	1
MIR3682	MIR3682	1	1
APRT	APRT	1	1
LINC00938	LINC00938	1	1
CSNK2B	CSNK2B	1	1
SAMD13	SAMD13	1	1
C3AR1	C3AR1	1	1
NDUFS7	NDUFS7	1	1
CHMP2A	CHMP2A	1	1
ARID1B	ARID1B	1	1
CDC26	CDC26	1	1
PIFO	PIFO	1	1
SSR1	SSR1	1	1
GON4L	GON4L	1	1
RIT2	RIT2	1	1
APOOL	APOOL	1	1
CLEC5A	CLEC5A	1	1
UBE2L6	UBE2L6	1	1
SEMA6D	SEMA6D	1	1
ZNF425	ZNF425	1	1
MS4A6A	MS4A6A	1	1

SH3BP5	SH3BP5	1	1
RNH1	RNH1	1	1
SLC11A2	SLC11A2	1	1
MT1E	MT1E	1	1
COL24A1	COL24A1	1	1
CDCA2	CDCA2	1	1
CAPNS1	CAPNS1	1	1
NFS1	NFS1	1	1
ZNF765	ZNF765	1	1
ARPC2	ARPC2	1	1
GATA4	GATA4	1	1
ZNF711	ZNF711	1	1
BCL11B	BCL11B	1	1
ABCA9	ABCA9	1	1
CWH43	CWH43	1	1
COA4	COA4	1	1
CFP	CFP	1	1
KLHL13	KLHL13	1	1
PIK3R3	PIK3R3	1	1
KAT7	KAT7	1	1
ERRFI1	ERRFI1	1	1
PIM3	PIM3	1	1
ZNF596	ZNF596	1	1
ZNF614	ZNF614	1	1
ATP6V0A4	ATP6V0A4	1	1
MEG9	MEG9	1	1
WNT2	WNT2	1	1
PEX2	PEX2	1	1
DDC-AS1	DDC-AS1	1	1
WRN	WRN	1	1
TFDP3	TFDP3	1	1
RMI1	RMI1	1	1
PON1	PON1	1	1
SIDT1	SIDT1	1	1
LOC10192884	LOC10192884	1	1
POLR3GL	POLR3GL	1	1
OLIG3	OLIG3	1	1
LOC10012945	LOC10012945	1	1
C21orf91-OT1	C21orf91-OT1	1	1
TRAF3IP2	TRAF3IP2	1	1
MAN2A1	MAN2A1	1	1
TOM1L1	TOM1L1	1	1
CTDSP2	CTDSP2	1	1
SEC62	SEC62	1	1
ADCY1	ADCY1	1	1
CYB561	CYB561	1	1
DLGAP1	DLGAP1	1	1
XG	XG	1	1
SNRPD1	SNRPD1	1	1
STK40	STK40	1	1

CDK2AP1	CDK2AP1	1	1
DGCR11	DGCR11	1	1
AOAH	AOAH	1	1
TRADD	TRADD	1	1
CCDC65	CCDC65	1	1
ZNF738	ZNF738	1	1
UBE2L3	UBE2L3	1	1
SLC35A2	SLC35A2	1	1
TTC22	TTC22	1	1
TGFBI	TGFBI	1	1
IL17A	IL17A	1	1
CLDN4	CLDN4	1	1
GZMB	GZMB	1	1
IARS2	IARS2	1	1
LACC1	LACC1	1	1
BRWD3	BRWD3	1	1
ADAMTS8	ADAMTS8	1	1
TMEM182	TMEM182	1	1
MPHOSPH8	MPHOSPH8	1	1
SH3PXD2A-AS	SH3PXD2A-AS	1	1
FOXQ1	FOXQ1	1	1
MGC12916	MGC12916	1	1
LINC00261	LINC00261	1	1
USP32	USP32	1	1
FAM90A1	FAM90A1	1	1
SOCS1	SOCS1	1	1
MYBL1	MYBL1	1	1
LGR4	LGR4	1	1
STARD4	STARD4	1	1
HAAO	HAAO	1	1
GPX4	GPX4	1	1
EEF1E1	EEF1E1	1	1
PHC3	PHC3	1	1
TXN	TXN	1	1
ZNF823	ZNF823	1	1
ZCCHC13	ZCCHC13	1	1
PROSER2-AS1	PROSER2-AS1	1	1
LY86	LY86	1	1
NINJ1	NINJ1	1	1
SYNGR1	SYNGR1	1	1
PDZRN3	PDZRN3	1	1
IPO9-AS1	IPO9-AS1	1	1
RASSF8	RASSF8	1	1
PSMG3	PSMG3	1	1
SH2B2	SH2B2	1	1
TUBB2A	TUBB2A	1	1
MYO18B	MYO18B	1	1
SEMA3E	SEMA3E	1	1
RLN2	RLN2	1	1
LOC10099675	LOC10099675	1	1

BTN3A2	BTN3A2	1	1
LCN12	LCN12	1	1
OR51B6	OR51B6	1	1
MYO1B	MYO1B	1	1
APOE	APOE	1	1
CAPZB	CAPZB	1	1
RRAS2	RRAS2	1	1
LINC00951	LINC00951	1	1
BID	BID	1	1
ATXN7L3B	ATXN7L3B	1	1
OR2W1	OR2W1	1	1
RNF103	RNF103	1	1
TCAIM	TCAIM	1	1
ALPK3	ALPK3	1	1
KIAA1217	KIAA1217	1	1
CPNE1	CPNE1	1	1
SLC25A5	SLC25A5	1	1
ASB15	ASB15	1	1
LNPEP	LNPEP	1	1
MBIP	MBIP	1	1
FRMD8P1	FRMD8P1	1	1
AFG3L2	AFG3L2	1	1
ATP13A3	ATP13A3	1	1
ELK3	ELK3	1	1
IGFLR1	IGFLR1	1	1
EPC2	EPC2	1	1
ADRB1	ADRB1	1	1
SH3BP5L	SH3BP5L	1	1
CKLF	CKLF	1	1
FAM135A	FAM135A	1	1
PCDHGC3	PCDHGC3	1	1
CCSER1	CCSER1	1	1
LANCL3	LANCL3	1	1
USP24	USP24	1	1
C4orf48	C4orf48	1	1
CIAO1	CIAO1	1	1
GLG1	GLG1	1	1
CCND2	CCND2	1	1
CCNYL2	CCNYL2	1	1
COPRS	COPRS	1	1
STAM	STAM	1	1
ARGLU1	ARGLU1	1	1
RETNLB	RETNLB	1	1
SBNO1	SBNO1	1	1
BMP10	BMP10	1	1
SIKE1	SIKE1	1	1
PKDCC	PKDCC	1	1
DEGS1	DEGS1	1	1
LINC00319	LINC00319	1	1
EIF2AK3	EIF2AK3	1	1

FNIP1	FNIP1	1	1
BDP1	BDP1	1	1
PDE1C	PDE1C	1	1
ACSM1	ACSM1	1	1
ANG	ANG	1	1
KLHL15	KLHL15	1	1
VPS28	VPS28	1	1
RPL19	RPL19	1	1
MAN2B1	MAN2B1	1	1
ANXA3	ANXA3	1	1
HSD17B11	HSD17B11	1	1
IL17F	IL17F	1	1
RRBP1	RRBP1	1	1
LAMC1	LAMC1	1	1
SMIM6	SMIM6	1	1
ZNF529	ZNF529	1	1
NPHP3	NPHP3	1	1
HLA-DMB	HLA-DMB	1	1
NUP160	NUP160	1	1
EIF3D	EIF3D	1	1
WNK4	WNK4	1	1
NRIP1	NRIP1	1	1
FLJ38576	FLJ38576	1	1
NEIL2	NEIL2	1	1
SMAD1	SMAD1	1	1
SPRY3	SPRY3	1	1
CLIP1	CLIP1	1	1
LINC00708	LINC00708	1	1
CSNK2A2	CSNK2A2	1	1
STOX2	STOX2	1	1
DNM1P35	DNM1P35	1	1
FAT4	FAT4	1	1
LYRM7	LYRM7	1	1
LINC00636	LINC00636	1	1
OPA1	OPA1	1	1
GPSM3	GPSM3	1	1
NPTX1	NPTX1	1	1
NEUROD1	NEUROD1	1	1
EYA1	EYA1	1	1
CD109	CD109	1	1
SELPLG	SELPLG	1	1
LINC00665	LINC00665	1	1
HLA-DMA	HLA-DMA	1	1
EIF4EBP1	EIF4EBP1	1	1
MST1L	MST1L	1	1
ERBB3	ERBB3	1	1
MRPL27	MRPL27	1	1
HSPB7	HSPB7	1	1
ASS1	ASS1	1	1
BAX	BAX	1	1

CHTOP	CHTOP	1	1
TRIM52-AS1	TRIM52-AS1	1	1
LRRC32	LRRC32	1	1
DIS3	DIS3	1	1
MYO1F	MYO1F	1	1
PGM5	PGM5	1	1
ALAD	ALAD	1	1
DND1	DND1	1	1
UTRN	UTRN	1	1
TRIM10	TRIM10	1	1
CUEDC2	CUEDC2	1	1
KLK7	KLK7	1	1
DOCK2	DOCK2	1	1
SULT4A1	SULT4A1	1	1
ITGA6	ITGA6	1	1
LINC00472	LINC00472	1	1
LTN1	LTN1	1	1
HMOX1	HMOX1	1	1
STARD13-AS	STARD13-AS	1	1
GYPC	GYPC	1	1
TAS1R1	TAS1R1	1	1
GAL	GAL	1	1
MACF1	MACF1	1	1
ALDH16A1	ALDH16A1	1	1
ICA1L	ICA1L	1	1
FBXW11	FBXW11	1	1
SMARCA2	SMARCA2	1	1
RSF1	RSF1	1	1
RAB5C	RAB5C	1	1
FARSA	FARSA	1	1
LOC1001309€	LOC1001309€	1	1
HCLS1	HCLS1	1	1
AQR	AQR	1	1
HADHB	HADHB	1	1
BBS4	BBS4	1	1
PLXDC2	PLXDC2	1	1
MYD88	MYD88	1	1
TCF25	TCF25	1	1
IFRD1	IFRD1	1	1
FBF1	FBF1	1	1
AFAP1	AFAP1	1	1
TMEM258	TMEM258	1	1
GPALPP1	GPALPP1	1	1
CTSK	CTSK	1	1
USP51	USP51	1	1
RMND5A	RMND5A	1	1
PLEKHM3	PLEKHM3	1	1
TMED5	TMED5	1	1
ARHGAP30	ARHGAP30	1	1
ZNF662	ZNF662	1	1

DPP4	DPP4	1	1
SYNM	SYNM	1	1
IL18RAP	IL18RAP	1	1
PARG	PARG	1	1
GDI2	GDI2	1	1
OXTR	OXTR	1	1
TRA2B	TRA2B	1	1
DUSP14	DUSP14	1	1
TLR2	TLR2	1	1
TPTE2P5	TPTE2P5	1	1
BTG2	BTG2	1	1
RAB11FIP2	RAB11FIP2	1	1
DUSP23	DUSP23	1	1
XIAP	XIAP	1	1
ZFAND6	ZFAND6	1	1
XKRX	XKRX	1	1
FAM98A	FAM98A	1	1
NAF1	NAF1	1	1
FBXO10	FBXO10	1	1
PDCD6	PDCD6	1	1
LILRB5	LILRB5	1	1
PLAG1	PLAG1	1	1
SPATS2	SPATS2	1	1
UBC	UBC	1	1
C12orf56	C12orf56	1	1
RAVER2	RAVER2	1	1
FIGN	FIGN	1	1
SPG7	SPG7	1	1
DMXL2	DMXL2	1	1
PDE7A	PDE7A	1	1
POLQ	POLQ	1	1
FIBP	FIBP	1	1
CPNE2	CPNE2	1	1
BLOC1S1	BLOC1S1	1	1
ELANE	ELANE	1	1
CAT	CAT	1	1
SYK	SYK	1	1
TMEM68	TMEM68	1	1
GFM1	GFM1	1	1
LAIR1	LAIR1	1	1
ETS2	ETS2	1	1
BRI3	BRI3	1	1
PRKY	PRKY	1	1
ZNF514	ZNF514	1	1
FAM201A	FAM201A	1	1
PRM2	PRM2	1	1
SET	SET	1	1
GTF2I	GTF2I	1	1
ARL5B	ARL5B	1	1
POU3F4	POU3F4	1	1

CPA5	CPA5	1	1
UBE4A	UBE4A	1	1
CEP70	CEP70	1	1
RBM27	RBM27	1	1
ZNF44	ZNF44	1	1
HCG18	HCG18	1	1
VAV1	VAV1	1	1
HSF2	HSF2	1	1
CHRNA1	CHRNA1	1	1
THEM4	THEM4	1	1
POLB	POLB	1	1
LHX4	LHX4	1	1
SOX7	SOX7	1	1
DNAJC7	DNAJC7	1	1
LONRF2	LONRF2	1	1
HAPLN1	HAPLN1	1	1
AGBL2	AGBL2	1	1
WDR47	WDR47	1	1
ALDH4A1	ALDH4A1	1	1
LOC339975	LOC339975	1	1
YOD1	YOD1	1	1
TICAM1	TICAM1	1	1
PRMT3	PRMT3	1	1
ZNF33B	ZNF33B	1	1
PRRG1	PRRG1	1	1
PPT1	PPT1	1	1
FZD1	FZD1	1	1
OLAH	OLAH	1	1
AKAP9	AKAP9	1	1
PWWP2A	PWWP2A	1	1
RAB13	RAB13	1	1
PRAME	PRAME	1	1
LOC1001289[illegible]	LOC1001289[illegible]	1	1
OXSR1	OXSR1	1	1
USP11	USP11	1	1
BEX5	BEX5	1	1
CDK4	CDK4	1	1
TMEM212	TMEM212	1	1
ILKAP	ILKAP	1	1
EPB41L4A-AS[illegible]	EPB41L4A-AS[illegible]	1	1
ELF2	ELF2	1	1
FOXP2	FOXP2	1	1
AGR2	AGR2	1	1
NDUFAF1	NDUFAF1	1	1
CDC42BPA	CDC42BPA	1	1
LOC1002407[illegible]	LOC1002407[illegible]	1	1
GBAP1	GBAP1	1	1
SLAMF8	SLAMF8	1	1
SNAPC4	SNAPC4	1	1
PLEK2	PLEK2	1	1

RBM12	RBM12	1	1
HCCAT5	HCCAT5	1	1
RPS27A	RPS27A	1	1
RAP1B	RAP1B	1	1
MRPL20	MRPL20	1	1
LITAF	LITAF	1	1
VCP	VCP	1	1
ZBTB2	ZBTB2	1	1
SRGAP2	SRGAP2	1	1
METRNL	METRNL	1	1
MIPOL1	MIPOL1	1	1
KCNV1	KCNV1	1	1
CCDC134	CCDC134	1	1
ATXN7	ATXN7	1	1
ST18	ST18	1	1
PSMD5	PSMD5	1	1
PIAS1	PIAS1	1	1
CLIC6	CLIC6	1	1
ERP44	ERP44	1	1
EXOSC6	EXOSC6	1	1
KCTD1	KCTD1	1	1
ARIH1	ARIH1	1	1
CD86	CD86	1	1
RPA1	RPA1	1	1
RBL1	RBL1	1	1
RASSF3	RASSF3	1	1
LOC10192724	LOC10192724	1	1
HCST	HCST	1	1
ARCN1	ARCN1	1	1
RNF166	RNF166	1	1
CLYBL-AS2	CLYBL-AS2	1	1
LOC728392	LOC728392	1	1
STX10	STX10	1	1
SETD3	SETD3	1	1
MKNK2	MKNK2	1	1
TMEM43	TMEM43	1	1
AKT3	AKT3	1	1
ZFP1	ZFP1	1	1
PITRM1-AS1	PITRM1-AS1	1	1
LINC00628	LINC00628	1	1
ENTPD1	ENTPD1	1	1
EVA1A	EVA1A	1	1
MRPL43	MRPL43	1	1
FAM221A	FAM221A	1	1
PRSS12	PRSS12	1	1
RAD23B	RAD23B	1	1
CABP7	CABP7	1	1
ODF2L	ODF2L	1	1
GSX2	GSX2	1	1
KDM2A	KDM2A	1	1

ADAM9	ADAM9	1	1
DCTN4	DCTN4	1	1
ABCC5	ABCC5	1	1
NUAK1	NUAK1	1	1
SIAE	SIAE	1	1
MECOM	MECOM	1	1
RPS6KA6	RPS6KA6	1	1
FTO	FTO	1	1
CBS	CBS	1	1
CACNA2D1	CACNA2D1	1	1
WWP1	WWP1	1	1
CXXC5	CXXC5	1	1
ATRX	ATRX	1	1
TRIM55	TRIM55	1	1
TSPO	TSPO	1	1
IL23R	IL23R	1	1
RSAD1	RSAD1	1	1
ZFP2	ZFP2	1	1
PHKG2	PHKG2	1	1
KCNA5	KCNA5	1	1
VOPP1	VOPP1	1	1
FOXA1	FOXA1	1	1
DMC1	DMC1	1	1
LINC01352	LINC01352	1	1
MTX3	MTX3	1	1
KRT40	KRT40	1	1
APBB1IP	APBB1IP	1	1
EFHC2	EFHC2	1	1
AKAP14	AKAP14	1	1
QPCT	QPCT	1	1
UBE2G1	UBE2G1	1	1
CHRM1	CHRM1	1	1
ADO	ADO	1	1
DPP10-AS3	DPP10-AS3	1	1
MYL6B	MYL6B	1	1
CYP1A2	CYP1A2	1	1
DCUN1D1	DCUN1D1	1	1
URB1	URB1	1	1
APOA2	APOA2	1	1
LOC10050607	LOC10050607	1	1
CRIP2	CRIP2	1	1
KCTD9	KCTD9	1	1
CCHCR1	CCHCR1	1	1
C9orf72	C9orf72	1	1
EXTL2	EXTL2	1	1
PDHB	PDHB	1	1
ERC2	ERC2	1	1
TPO	TPO	1	1
LRBA	LRBA	1	1
RBM17	RBM17	1	1

TUSC3	TUSC3	1	1
FAM186A	FAM186A	1	1
CD96	CD96	1	1
ZNF813	ZNF813	1	1
HAVCR2	HAVCR2	1	1
CTSC	CTSC	1	1
LMO3	LMO3	1	1
GUSB	GUSB	1	1
TPGS1	TPGS1	1	1
PPP3R1	PPP3R1	1	1
ZNF233	ZNF233	1	1
VPS13A	VPS13A	1	1
CD47	CD47	1	1
ITGAV	ITGAV	1	1
GHSR	GHSR	1	1
RPRD1A	RPRD1A	1	1
SLC39A14	SLC39A14	1	1
PTPRD	PTPRD	1	1
SIRT1	SIRT1	1	1
UBE2S	UBE2S	1	1
GNA15	GNA15	1	1
ARHGAP6	ARHGAP6	1	1
ATAD2B	ATAD2B	1	1
CTDP1	CTDP1	1	1
TOLLIP	TOLLIP	1	1
FBP1	FBP1	1	1
STXBP5L	STXBP5L	1	1
FTH1	FTH1	1	1
IL13RA1	IL13RA1	1	1
PELP1	PELP1	1	1
PTP4A2	PTP4A2	1	1
FAM172A	FAM172A	1	1
KIF11	KIF11	1	1
BSG	BSG	1	1
SP6	SP6	1	1
MCF2	MCF2	1	1
UNKL	UNKL	1	1
KIF3A	KIF3A	1	1
RNASE2	RNASE2	1	1
SLC1A5	SLC1A5	1	1
SPAG4	SPAG4	1	1
PRKCA	PRKCA	1	1
PPP1CA	PPP1CA	1	1
FBXO30	FBXO30	1	1
LINC01364	LINC01364	1	1
KLF3	KLF3	1	1
LMAN2	LMAN2	1	1
MRPL2	MRPL2	1	1
GANAB	GANAB	1	1
GHRH	GHRH	1	1

NCF4	NCF4	1	1
FRMD6-AS1	FRMD6-AS1	1	1
ZNF215	ZNF215	1	1
POMGNT2	POMGNT2	1	1
TREH	TREH	1	1
FHL2	FHL2	1	1
PRMT2	PRMT2	1	1
MTFMT	MTFMT	1	1
OR8G1	OR8G1	1	1
CENPN	CENPN	1	1
RPL36AL	RPL36AL	1	1
PRIMA1	PRIMA1	1	1
PPARGC1A	PPARGC1A	1	1
ZWINT	ZWINT	1	1
AJUBA	AJUBA	1	1
DCSTAMP	DCSTAMP	1	1
PSMB3	PSMB3	1	1
ZFP37	ZFP37	1	1
CRCT1	CRCT1	1	1
SF3B1	SF3B1	1	1
CCDC85B	CCDC85B	1	1
POLR3B	POLR3B	1	1
RAMP2-AS1	RAMP2-AS1	1	1
VAMP3	VAMP3	1	1
NPM3	NPM3	1	1
TFAP2D	TFAP2D	1	1
NSMF	NSMF	1	1
TMEM87A	TMEM87A	1	1
AHSG	AHSG	1	1
SULT1B1	SULT1B1	1	1
LIMD1	LIMD1	1	1
LCT	LCT	1	1
ARMCX2	ARMCX2	1	1
ENTPD4	ENTPD4	1	1
LINC00029	LINC00029	1	1
DOK2	DOK2	1	1
NUDT13	NUDT13	1	1
TLR10	TLR10	1	1
WNT5A	WNT5A	1	1
BBX	BBX	1	1
ACSBG2	ACSBG2	1	1
HLTF	HLTF	1	1
LSM14A	LSM14A	1	1
LINC00452	LINC00452	1	1
IREB2	IREB2	1	1
YWHAE	YWHAE	1	1
CASS4	CASS4	1	1
VENTX	VENTX	1	1
CCDC171	CCDC171	1	1
GOPC	GOPC	1	1

LAMA5	LAMA5	1	1
KLHDC3	KLHDC3	1	1
ZNF304	ZNF304	1	1
ELF1	ELF1	1	1
SCML4	SCML4	1	1
VSNL1	VSNL1	1	1
PRCP	PRCP	1	1
PCDHAC1	PCDHAC1	1	1
SMARCAD1	SMARCAD1	1	1
ADAM20	ADAM20	1	1
FRRS1L	FRRS1L	1	1
TAF1L	TAF1L	1	1
ZC3HAV1L	ZC3HAV1L	1	1
JPH2	JPH2	1	1
SLC16A14	SLC16A14	1	1
CCT7	CCT7	1	1
CDH10	CDH10	1	1
IP6K2	IP6K2	1	1
MADCAM1	MADCAM1	1	1
LRRC58	LRRC58	1	1
LINC01428	LINC01428	1	1
LIPA	LIPA	1	1
ACVR2A	ACVR2A	1	1
ASCC3	ASCC3	1	1
CSK	CSK	1	1
PREX1	PREX1	1	1
XBP1	XBP1	1	1
EFR3B	EFR3B	1	1
TIFA	TIFA	1	1
RPS18	RPS18	1	1
EXOC8	EXOC8	1	1
PRC1	PRC1	1	1
RASAL2	RASAL2	1	1
KLF6	KLF6	1	1
DPPA2	DPPA2	1	1
CYP2C9	CYP2C9	1	1
LMTK3	LMTK3	1	1
ARL3	ARL3	1	1
GABRA4	GABRA4	1	1
GPR35	GPR35	1	1
PHLDB3	PHLDB3	1	1
TBC1D19	TBC1D19	1	1
SOX4	SOX4	1	1
CLRN1	CLRN1	1	1
KLK2	KLK2	1	1
TRIM31	TRIM31	1	1
ATP8B3	ATP8B3	1	1
METAP1	METAP1	1	1
CCM2	CCM2	1	1
COL17A1	COL17A1	1	1

CHSY3	CHSY3	1	1
IPO11	IPO11	1	1
LOC10192962	LOC10192962	1	1
EMX1	EMX1	1	1
MPHOSPH9	MPHOSPH9	1	1
RFC3	RFC3	1	1
DPEP2	DPEP2	1	1
KLHDC7A	KLHDC7A	1	1
DSG1	DSG1	1	1
CMIP	CMIP	1	1
HMGN3-AS1	HMGN3-AS1	1	1
KPNA3	KPNA3	1	1
PAK1IP1	PAK1IP1	1	1
GCC2	GCC2	1	1
PPP1R3F	PPP1R3F	1	1
C21orf91	C21orf91	1	1
UBE2D1	UBE2D1	1	1
MECP2	MECP2	1	1
RPL18	RPL18	1	1
ING3	ING3	1	1
ZCCHC2	ZCCHC2	1	1
C15orf32	C15orf32	1	1
AEBP2	AEBP2	1	1
AGPAT5	AGPAT5	1	1
GPR155	GPR155	1	1
SDHD	SDHD	1	1
DISP1	DISP1	1	1
LIMD2	LIMD2	1	1
PEBP1	PEBP1	1	1
ZNF286A	ZNF286A	1	1
AZI2	AZI2	1	1
UBE2K	UBE2K	1	1
DCLRE1C	DCLRE1C	1	1
CARHSP1	CARHSP1	1	1
MED14OS	MED14OS	1	1
HOOK3	HOOK3	1	1
CRYGA	CRYGA	1	1
COMMD5	COMMD5	1	1
SOCS5	SOCS5	1	1
CKS1B	CKS1B	1	1
SF3A3	SF3A3	1	1
TSSK1B	TSSK1B	1	1
TSLP	TSLP	1	1
ZNF224	ZNF224	1	1
METTL21EP	METTL21EP	1	1
LINC00880	LINC00880	1	1
MYL9	MYL9	1	1
STXBP2	STXBP2	1	1
MIAT	MIAT	1	1
TNNT3	TNNT3	1	1

LYSMD4	LYSMD4	1	1
COLCA1	COLCA1	1	1
TMEM179	TMEM179	1	1
ERGIC3	ERGIC3	1	1
VMO1	VMO1	1	1
MARVELD3	MARVELD3	1	1
ACVR2B	ACVR2B	1	1
UBL7	UBL7	1	1
ZKSCAN7	ZKSCAN7	1	1
SRP54	SRP54	1	1
NRG3	NRG3	1	1
FAM184A	FAM184A	1	1
HYMAI	HYMAI	1	1
DNAJC21	DNAJC21	1	1
TNFAIP2	TNFAIP2	1	1
APH1A	APH1A	1	1
GOLT1B	GOLT1B	1	1
LOC1019269(	LOC1019269(	1	1
NT5DC1	NT5DC1	1	1
RFX7	RFX7	1	1
HTRA4	HTRA4	1	1
ATXN1	ATXN1	1	1
LINC00572	LINC00572	1	1
METTL2B	METTL2B	1	1
ATP6AP1L	ATP6AP1L	1	1
KCNIP2-AS1	KCNIP2-AS1	1	1
ADAM11	ADAM11	1	1
RPL8	RPL8	1	1
C3orf52	C3orf52	1	1
LTC4S	LTC4S	1	1
MRPS12	MRPS12	1	1
SEMA4B	SEMA4B	1	1
IKZF5	IKZF5	1	1
SSH2	SSH2	1	1
HGSNAT	HGSNAT	1	1
FANCL	FANCL	1	1
UTP14A	UTP14A	1	1
EPC1	EPC1	1	1
C5orf24	C5orf24	1	1
CDC27	CDC27	1	1
TMEM150B	TMEM150B	1	1
PRDX5	PRDX5	1	1
ESYT3	ESYT3	1	1
ARHGDIB	ARHGDIB	1	1
SIGLEC16	SIGLEC16	1	1
RAET1E	RAET1E	1	1
MSL3	MSL3	1	1
ADORA1	ADORA1	1	1
GABRQ	GABRQ	1	1
KCP	KCP	1	1

RLIM	RLIM	1	1
SIM2	SIM2	1	1
RAB1B	RAB1B	1	1
PRICKLE2	PRICKLE2	1	1
LINC01140	LINC01140	1	1
LINC00208	LINC00208	1	1
TTC39C	TTC39C	1	1
GPR82	GPR82	1	1
PPARA	PPARA	1	1
ERCC6L2	ERCC6L2	1	1
IFNW1	IFNW1	1	1
C10orf71	C10orf71	1	1
ACAD9	ACAD9	1	1
EN1	EN1	1	1
BCAP29	BCAP29	1	1
CPOX	CPOX	1	1
CLDN12	CLDN12	1	1
TM4SF4	TM4SF4	1	1
RALGAPB	RALGAPB	1	1
HHEX	HHEX	1	1
DDX23	DDX23	1	1
CASQ2	CASQ2	1	1
IL12RB2	IL12RB2	1	1
KLHL31	KLHL31	1	1
UGGT2	UGGT2	1	1
TCEAL1	TCEAL1	1	1
STARD8	STARD8	1	1
UBQLN1	UBQLN1	1	1
C7	C7	1	1
ABCB7	ABCB7	1	1
PRKAR2A	PRKAR2A	1	1
RYBP	RYBP	1	1
VPS36	VPS36	1	1
GSR	GSR	1	1
STK16	STK16	1	1
ALOX5	ALOX5	1	1
ATXN10	ATXN10	1	1
TERF1	TERF1	1	1
ATP6V0D1	ATP6V0D1	1	1
MMP11	MMP11	1	1
PPP2R5E	PPP2R5E	1	1
COPZ2	COPZ2	1	1
KAT2A	KAT2A	1	1
EREG	EREG	1	1
CTDNEP1	CTDNEP1	1	1
VAMP5	VAMP5	1	1
MRC1	MRC1	1	1
NOX4	NOX4	1	1
CACNG4	CACNG4	1	1
RNF115	RNF115	1	1

BAIAP2L2	BAIAP2L2	1	1
TNRC6C	TNRC6C	1	1
SUPT4H1	SUPT4H1	1	1
PICK1	PICK1	1	1
SERINC3	SERINC3	1	1
TUFM	TUFM	1	1
SACM1L	SACM1L	1	1
IL20RA	IL20RA	1	1
NXPE3	NXPE3	1	1
RRM2B	RRM2B	1	1
PSMC5	PSMC5	1	1
UBP1	UBP1	1	1
HINFP	HINFP	1	1
IFNL1	IFNL1	1	1
ZBTB37	ZBTB37	1	1
PLEKHA5	PLEKHA5	1	1
SLC13A4	SLC13A4	1	1
SORBS3	SORBS3	1	1
MRPL10	MRPL10	1	1
ABHD17C	ABHD17C	1	1
OR1D5	OR1D5	1	1
ZZZ3	ZZZ3	1	1
GTPBP6	GTPBP6	1	1
GPD1L	GPD1L	1	1
SLCO1B3	SLCO1B3	1	1
SNX4	SNX4	1	1
KCND3	KCND3	1	1
CSF2RA	CSF2RA	1	1
IL32	IL32	1	1
LEPROT	LEPROT	1	1
LRRC57	LRRC57	1	1
TMTC1	TMTC1	1	1
ERICH1	ERICH1	1	1
HAO1	HAO1	1	1
ACVR1	ACVR1	1	1
GCNT2	GCNT2	1	1
MAU2	MAU2	1	1
JMJD8	JMJD8	1	1
ZNF391	ZNF391	1	1
PPDPF	PPDPF	1	1
PBXIP1	PBXIP1	1	1
E2F5	E2F5	1	1
SPOCK2	SPOCK2	1	1
NBPF3	NBPF3	1	1
ATRN	ATRN	1	1
SNRNP40	SNRNP40	1	1
KRTAP3-1	KRTAP3-1	1	1
LINC00462	LINC00462	1	1
CLEC4E	CLEC4E	1	1
ERF	ERF	1	1

GPX1	GPX1	1	1
C20orf173	C20orf173	1	1
EDDM3A	EDDM3A	1	1
DDX41	DDX41	1	1
MLYCD	MLYCD	1	1
OTUD1	OTUD1	1	1
VEZT	VEZT	1	1
ERVMER34-1	ERVMER34-1	1	1
ROPN1	ROPN1	1	1
ZNF865	ZNF865	1	1
UROC1	UROC1	1	1
ANAPC15	ANAPC15	1	1
FER1L6-AS1	FER1L6-AS1	1	1
MRPL13	MRPL13	1	1
NANOS1	NANOS1	1	1
GALNT7	GALNT7	1	1
PRDM4	PRDM4	1	1
YES1	YES1	1	1
DCBLD2	DCBLD2	1	1
RALGDS	RALGDS	1	1
INHA	INHA	1	1
CCDC38	CCDC38	1	1
RALGAPA1	RALGAPA1	1	1
SHC1	SHC1	1	1
CNNM1	CNNM1	1	1
KL	KL	1	1
PANK1	PANK1	1	1
STBD1	STBD1	1	1
SLC23A3	SLC23A3	1	1
SCAF8	SCAF8	1	1
ENO1	ENO1	1	1
KAT8	KAT8	1	1
FBXO24	FBXO24	1	1
LRRC20	LRRC20	1	1
SNRNP27	SNRNP27	1	1
DLG5-AS1	DLG5-AS1	1	1
PROS1	PROS1	1	1
RIMS3	RIMS3	1	1
SLC15A3	SLC15A3	1	1
CREM	CREM	1	1
LOC10272383	LOC10272383	1	1
MAF	MAF	1	1
SLC26A2	SLC26A2	1	1
ZBTB18	ZBTB18	1	1
LAS1L	LAS1L	1	1
HTR4	HTR4	1	1
RANBP3	RANBP3	1	1
LOC10013404	LOC10013404	1	1
IGBP1	IGBP1	1	1
ADAP1	ADAP1	1	1

RAB35	RAB35	1	1
KRT35	KRT35	1	1
PYHIN1	PYHIN1	1	1
STX4	STX4	1	1
PRRX2-AS1	PRRX2-AS1	1	1
COMMD7	COMMD7	1	1
HLA-E	HLA-E	1	1
CCR3	CCR3	1	1
MAPK8	MAPK8	1	1
RBAK	RBAK	1	1
GABRR2	GABRR2	1	1
LYN	LYN	1	1
NPAS3	NPAS3	1	1
ARPC1A	ARPC1A	1	1
RFK	RFK	1	1
PPFIA4	PPFIA4	1	1
MTIF2	MTIF2	1	1
MSL2	MSL2	1	1
ABCB5	ABCB5	1	1
SQSTM1	SQSTM1	1	1
ZNF423	ZNF423	1	1
TSHR	TSHR	1	1
RILPL2	RILPL2	1	1
FADS2	FADS2	1	1
TENM1	TENM1	1	1
ARHGAP29	ARHGAP29	1	1
PPP1R8	PPP1R8	1	1
TSPYL1	TSPYL1	1	1
STK10	STK10	1	1
KLF11	KLF11	1	1
IDH3B	IDH3B	1	1
FNIP2	FNIP2	1	1
VTA1	VTA1	1	1
NUDT5	NUDT5	1	1
C2orf49	C2orf49	1	1
GTF2F2	GTF2F2	1	1
PLEKHF1	PLEKHF1	1	1
PSMC4	PSMC4	1	1
PSORS1C2	PSORS1C2	1	1
SLCO2B1	SLCO2B1	1	1
TMEM139	TMEM139	1	1
DOK5	DOK5	1	1
SNAI3	SNAI3	1	1
CD53	CD53	1	1
RAB12	RAB12	1	1
MYO1H	MYO1H	1	1
THRA	THRA	1	1
LRR1	LRR1	1	1
LIMA1	LIMA1	1	1
APOF	APOF	1	1

PLVAP	PLVAP	1	1
GSTM5	GSTM5	1	1
FBXO7	FBXO7	1	1
RBX1	RBX1	1	1
ARHGAP9	ARHGAP9	1	1
CHURC1	CHURC1	1	1
VILL	VILL	1	1
TMEM165	TMEM165	1	1
KIR3DS1	KIR3DS1	1	1
MOGAT2	MOGAT2	1	1
PRELID1	PRELID1	1	1
CD9	CD9	1	1
SBF2	SBF2	1	1
ZNF180	ZNF180	1	1
DUSP16	DUSP16	1	1
CHUK	CHUK	1	1
ZNF280B	ZNF280B	1	1
FIBIN	FIBIN	1	1
FOXR1	FOXR1	1	1
FLCN	FLCN	1	1
OXSM	OXSM	1	1
RCAN3	RCAN3	1	1
TBC1D10A	TBC1D10A	1	1
INTS2	INTS2	1	1
KANSL2	KANSL2	1	1
KDELR1	KDELR1	1	1
SLC35F5	SLC35F5	1	1
DAPK2	DAPK2	1	1
LOC10192881	LOC10192881	1	1
FRK	FRK	1	1
NUF2	NUF2	1	1
THAP8	THAP8	1	1
SMCR8	SMCR8	1	1
TLR5	TLR5	1	1
ORC2	ORC2	1	1
LIG1	LIG1	1	1
RMI2	RMI2	1	1
DPF3	DPF3	1	1
DIP2C	DIP2C	1	1
DENND5B	DENND5B	1	1
MDN1	MDN1	1	1
DIAPH3	DIAPH3	1	1
COL22A1	COL22A1	1	1
OSTF1	OSTF1	1	1
TPI1	TPI1	1	1
FANCM	FANCM	1	1
PMCH	PMCH	1	1
NAP1L3	NAP1L3	1	1
KLHL7	KLHL7	1	1
CDKN2D	CDKN2D	1	1

LOC646762	LOC646762	1	1
FBXW5	FBXW5	1	1
ANKFY1	ANKFY1	1	1
RYK	RYK	1	1
PROSER3	PROSER3	1	1
C12orf40	C12orf40	1	1
MRO	MRO	1	1
TRMT2B	TRMT2B	1	1
BZW2	BZW2	1	1
ZNF512B	ZNF512B	1	1
TANC1	TANC1	1	1
NUDT19	NUDT19	1	1
ZNF793	ZNF793	1	1
MBTPS1	MBTPS1	1	1
PMS2P4	PMS2P4	1	1
SGCA	SGCA	1	1
NDRG4	NDRG4	1	1
RAF1	RAF1	1	1
PIK3R5	PIK3R5	1	1
SCAND1	SCAND1	1	1
MYO5C	MYO5C	1	1
LOC1027249€	LOC1027249€	1	1
RAB3GAP1	RAB3GAP1	1	1
VIPR1	VIPR1	1	1
ZNF461	ZNF461	1	1
SYP	SYP	1	1
BIVM	BIVM	1	1
TCIRG1	TCIRG1	1	1
BFSP2-AS1	BFSP2-AS1	1	1
TMEM160	TMEM160	1	1
PHF12	PHF12	1	1
TMEM223	TMEM223	1	1
SUPT6H	SUPT6H	1	1
EEF1D	EEF1D	1	1
CHMP4C	CHMP4C	1	1
SAMHD1	SAMHD1	1	1
TECTA	TECTA	1	1
FLYWCH2	FLYWCH2	1	1
PICALM	PICALM	1	1
ENAM	ENAM	1	1
NLGN1	NLGN1	1	1
GJA4	GJA4	1	1
C1RL-AS1	C1RL-AS1	1	1
IMP3	IMP3	1	1
LTV1	LTV1	1	1
YTHDF3	YTHDF3	1	1
KIF13A	KIF13A	1	1
ZNF14	ZNF14	1	1
KRT72	KRT72	1	1
HMGCR	HMGCR	1	1

TDRG1	TDRG1	1	1
AHCY	AHCY	1	1
FAM181A-AS:	FAM181A-AS:	1	1
MXRA8	MXRA8	1	1
ILF2	ILF2	1	1
MAGI2	MAGI2	1	1
LOC93622	LOC93622	1	1
RGMB	RGMB	1	1
MOV10	MOV10	1	1
RPS6KB1	RPS6KB1	1	1
DIS3L2	DIS3L2	1	1
SYT3	SYT3	1	1
FNTA	FNTA	1	1
NETO2	NETO2	1	1
SNAP25	SNAP25	1	1
MEF2A	MEF2A	1	1
LPXN	LPXN	1	1
INSR	INSR	1	1
E2F7	E2F7	1	1
LASP1	LASP1	1	1
SRL	SRL	1	1
HABP2	HABP2	1	1
S100A3	S100A3	1	1
FBXO31	FBXO31	1	1
RRAGB	RRAGB	1	1
ZNF600	ZNF600	1	1
PPP1R14C	PPP1R14C	1	1
TMEM114	TMEM114	1	1
SIK2	SIK2	1	1
PRKAG1	PRKAG1	1	1
SLC12A6	SLC12A6	1	1
ST13	ST13	1	1
ABHD17A	ABHD17A	1	1
MVB12A	MVB12A	1	1
EGLN2	EGLN2	1	1
SSNA1	SSNA1	1	1
COL6A1	COL6A1	1	1
CADPS2	CADPS2	1	1
DHRS3	DHRS3	1	1
LOC10192709	LOC10192709	1	1
MORN5	MORN5	1	1
KRT80	KRT80	1	1
FBXO34	FBXO34	1	1
TCN2	TCN2	1	1
PREB	PREB	1	1
MYH2	MYH2	1	1
KLHL8	KLHL8	1	1
COPS6	COPS6	1	1
TTYH3	TTYH3	1	1
GNB1	GNB1	1	1

PSMB10	PSMB10	1	1
LOC10192825	LOC10192825	1	1
MET	MET	1	1
ILK	ILK	1	1
ARRB2	ARRB2	1	1
FBXO6	FBXO6	1	1
ZNF587B	ZNF587B	1	1
RCN2	RCN2	1	1
FGF1	FGF1	1	1
ANGEL2	ANGEL2	1	1
ARID5A	ARID5A	1	1
DHRS1	DHRS1	1	1
CKM	CKM	1	1
AP3D1	AP3D1	1	1
ASXL2	ASXL2	1	1
RAC3	RAC3	1	1
P2RY8	P2RY8	1	1
PITPNB	PITPNB	1	1
ZFP62	ZFP62	1	1
NTAN1	NTAN1	1	1
GORASP1	GORASP1	1	1
PRDX6	PRDX6	1	1
GSPT2	GSPT2	1	1
IGFL1	IGFL1	1	1
CA5BP1	CA5BP1	1	1
CLEC7A	CLEC7A	1	1
F7	F7	1	1
ZBED5	ZBED5	1	1
MYOG	MYOG	1	1
NFIB	NFIB	1	1
NPPC	NPPC	1	1
LANCL2	LANCL2	1	1
RRP36	RRP36	1	1
MAP7D1	MAP7D1	1	1
ZNF407	ZNF407	1	1
ZNF606	ZNF606	1	1
ZDHHC22	ZDHHC22	1	1
SUZ12	SUZ12	1	1
GATA3	GATA3	1	1
CUX2	CUX2	1	1
AIF1	AIF1	1	1
TNFRSF4	TNFRSF4	1	1
CBR3	CBR3	1	1
TMEM191A	TMEM191A	1	1
GUCY2C	GUCY2C	1	1
SIRPB1	SIRPB1	1	1
PCLO	PCLO	1	1
ZNF343	ZNF343	1	1
TOP3B	TOP3B	1	1
ABHD14B	ABHD14B	1	1

LOC10050751	LOC10050751	1	1
CYP2C8	CYP2C8	1	1
LINC00491	LINC00491	1	1
C3orf36	C3orf36	1	1
TCFL5	TCFL5	1	1
MRPL34	MRPL34	1	1
ZFP92	ZFP92	1	1
GSTK1	GSTK1	1	1
LOC10099668	LOC10099668	1	1
SEPSECS-AS1	SEPSECS-AS1	1	1
CA9	CA9	1	1
CDC34	CDC34	1	1
SEC23B	SEC23B	1	1
DTX2P1-UPK3	DTX2P1-UPK3	1	1
PKD1L1	PKD1L1	1	1
PLP2	PLP2	1	1
GAMT	GAMT	1	1
RTP3	RTP3	1	1
VRK2	VRK2	1	1
COX6A1	COX6A1	1	1
BCL2L10	BCL2L10	1	1
PCYOX1	PCYOX1	1	1
ITGA9-AS1	ITGA9-AS1	1	1
KNCN	KNCN	1	1
FERMT1	FERMT1	1	1
FOXO1	FOXO1	1	1
DAXX	DAXX	1	1
LY9	LY9	1	1
NR1H4	NR1H4	1	1
NAA60	NAA60	1	1
CNTFR	CNTFR	1	1
CLEC14A	CLEC14A	1	1
LINC00674	LINC00674	1	1
EBI3	EBI3	1	1
POLR2E	POLR2E	1	1
VAC14	VAC14	1	1
WNT9A	WNT9A	1	1
LINC01138	LINC01138	1	1
TAPT1	TAPT1	1	1
GRPEL2	GRPEL2	1	1
GAL3ST4	GAL3ST4	1	1
CLDND2	CLDND2	1	1
LILRB2	LILRB2	1	1
LINC00540	LINC00540	1	1
RP9	RP9	1	1
IQCJ-SCHIP1-A	IQCJ-SCHIP1-A	1	1
PAX9	PAX9	1	1
FCGRT	FCGRT	1	1
LOC10192765	LOC10192765	1	1
LOC10192760	LOC10192760	1	1

RHOBTB3	RHOBTB3	1	1
VANGL1	VANGL1	1	1
ZC3H12C	ZC3H12C	1	1
DDX52	DDX52	1	1
BCLAF1	BCLAF1	1	1
HIPK1	HIPK1	1	1
PRDM5	PRDM5	1	1
BUD31	BUD31	1	1
PECR	PECR	1	1
RPS19	RPS19	1	1
ARHGAP26	ARHGAP26	1	1
PATZ1	PATZ1	1	1
ADTRP	ADTRP	1	1
AKAP6	AKAP6	1	1
BBS10	BBS10	1	1
SETX	SETX	1	1
DTNA	DTNA	1	1
MRPL14	MRPL14	1	1
TBC1D30	TBC1D30	1	1
SMIM3	SMIM3	1	1
IL5RA	IL5RA	1	1
FAM168A	FAM168A	1	1
UXS1	UXS1	1	1
CTNNBIP1	CTNNBIP1	1	1
CYP2A7	CYP2A7	1	1
ADAM10	ADAM10	1	1
CLN3	CLN3	1	1
VCL	VCL	1	1
CHIT1	CHIT1	1	1
ERP29	ERP29	1	1
PRDM2	PRDM2	1	1
CAPN8	CAPN8	1	1
MARK4	MARK4	1	1
PHB2	PHB2	1	1
MSI1	MSI1	1	1
BCL2L1	BCL2L1	1	1
PDE4A	PDE4A	1	1
LOC220077	LOC220077	1	1
MEIS3P1	MEIS3P1	1	1
TIGD6	TIGD6	1	1
MRPL33	MRPL33	1	1
TTYH1	TTYH1	1	1
UBR2	UBR2	1	1
RIN2	RIN2	1	1
TP73-AS1	TP73-AS1	1	1
BTLA	BTLA	1	1
PTPMT1	PTPMT1	1	1
MRPS16	MRPS16	1	1
GLRX2	GLRX2	1	1
C1orf115	C1orf115	1	1

OVOL2	OVOL2	1	1
VDAC1	VDAC1	1	1
GTF3C2-AS1	GTF3C2-AS1	1	1
PPP4R2	PPP4R2	1	1
HSPB3	HSPB3	1	1
HOXB8	HOXB8	1	1
NUDT9P1	NUDT9P1	1	1
CDH24	CDH24	1	1
PSMB8	PSMB8	1	1
TTC19	TTC19	1	1
CASP12	CASP12	1	1
KRT7	KRT7	1	1
MUC17	MUC17	1	1
RETN	RETN	1	1
USP33	USP33	1	1
RNF146	RNF146	1	1
SEC24C	SEC24C	1	1
COG7	COG7	1	1
HSPB2	HSPB2	1	1
BPIFC	BPIFC	1	1
LINC01015	LINC01015	1	1
HORMAD2	HORMAD2	1	1
CNTN2	CNTN2	1	1
LOC1019282€	LOC1019282€	1	1
WSB2	WSB2	1	1
TRPC1	TRPC1	1	1
PIP4K2A	PIP4K2A	1	1
PSMB6	PSMB6	1	1
RHOC	RHOC	1	1
TXNDC12	TXNDC12	1	1
SPINK1	SPINK1	1	1
MMP10	MMP10	1	1
LMNB1	LMNB1	1	1
SCARNA15	SCARNA15	1	1
ATOX1	ATOX1	1	1
ZNF763	ZNF763	1	1
SOCS2-AS1	SOCS2-AS1	1	1
SLC38A2	SLC38A2	1	1
ZFP69	ZFP69	1	1
CYP2B7P	CYP2B7P	1	1
SMIM13	SMIM13	1	1
FZD4	FZD4	1	1
RFTN2	RFTN2	1	1
COL16A1	COL16A1	1	1
RPS5	RPS5	1	1
UBE2T	UBE2T	1	1
RDH8	RDH8	1	1
LALBA	LALBA	1	1
HEXA	HEXA	1	1
ACBD3	ACBD3	1	1

FKBP8	FKBP8	1	1
NTN5	NTN5	1	1
TMEM143	TMEM143	1	1
C5orf22	C5orf22	1	1
MSN	MSN	1	1
MEX3B	MEX3B	1	1
PRORY	PRORY	1	1
CLCN6	CLCN6	1	1
MYO9A	MYO9A	1	1
CETP	CETP	1	1
LOC153910	LOC153910	1	1
RAB3IP	RAB3IP	1	1
TDRD3	TDRD3	1	1
CD6	CD6	1	1
RAP2C	RAP2C	1	1
ZEB1-AS1	ZEB1-AS1	1	1
SPAG6	SPAG6	1	1
LOC441179	LOC441179	1	1
IDO2	IDO2	1	1
SCN3B	SCN3B	1	1
TBC1D9	TBC1D9	1	1
FOXE3	FOXE3	1	1
ZFP36L1	ZFP36L1	1	1
KIAA1522	KIAA1522	1	1
ECI1	ECI1	1	1
MAFB	MAFB	1	1
RNF181	RNF181	1	1
KCTD19	KCTD19	1	1
CFDP1	CFDP1	1	1
SCARNA2	SCARNA2	1	1
BARX2	BARX2	1	1
GRB7	GRB7	1	1
OSBPL1A	OSBPL1A	1	1
ARHGEF3	ARHGEF3	1	1
BRAP	BRAP	1	1
CAMTA1	CAMTA1	1	1
ZNF354C	ZNF354C	1	1
SSR3	SSR3	1	1
LOC646736	LOC646736	1	1
SYCE1L	SYCE1L	1	1
SNRPD3	SNRPD3	1	1
EVI5L	EVI5L	1	1
CD44	CD44	1	1
AAMDC	AAMDC	1	1
LINC00927	LINC00927	1	1
DPP3	DPP3	1	1
BIN2	BIN2	1	1
TRIM6	TRIM6	1	1
MOGS	MOGS	1	1
KLHL3	KLHL3	1	1

KCNS1	KCNS1	1	1
ZNF615	ZNF615	1	1
ZMAT4	ZMAT4	1	1
LOC10272461	LOC10272461	1	1
GLOD5	GLOD5	1	1
LOC1001296C	LOC1001296C	1	1
CNTNAP4	CNTNAP4	1	1
TRIM45	TRIM45	1	1
AS3MT	AS3MT	1	1
TOR1B	TOR1B	1	1
COMMD10	COMMD10	1	1
ZYG11B	ZYG11B	1	1
RPRD2	RPRD2	1	1
ATP2B4	ATP2B4	1	1
LINC01003	LINC01003	1	1
ERICH5	ERICH5	1	1
PSG4	PSG4	1	1
EFEMP2	EFEMP2	1	1
CHID1	CHID1	1	1
CISD1	CISD1	1	1
UBE2NL	UBE2NL	1	1
SUMO3	SUMO3	1	1
ZNF776	ZNF776	1	1
GSTZ1	GSTZ1	1	1
TTLL2	TTLL2	1	1
CTSW	CTSW	1	1
EIF4G2	EIF4G2	1	1
EID2	EID2	1	1
TACR3	TACR3	1	1
PARVG	PARVG	1	1
WDR75	WDR75	1	1
HDAC11	HDAC11	1	1
CREBRF	CREBRF	1	1
WDR53	WDR53	1	1
C4orf45	C4orf45	1	1
CDH8	CDH8	1	1
IFNG	IFNG	1	1
TMSB10	TMSB10	1	1
ALG6	ALG6	1	1
PLD1	PLD1	1	1
CYCS	CYCS	1	1
RBMXL1	RBMXL1	1	1
RBPMS	RBPMS	1	1
IQCE	IQCE	1	1
AVEN	AVEN	1	1
PRKAB2	PRKAB2	1	1
TOR1AIP2	TOR1AIP2	1	1
GRWD1	GRWD1	1	1
MPV17	MPV17	1	1
LOC10050692	LOC10050692	1	1

CD2BP2	CD2BP2	1	1
TUB	TUB	1	1
ZNF469	ZNF469	1	1
TRIM5	TRIM5	1	1
GLIPR1L1	GLIPR1L1	1	1
IL22	IL22	1	1
COPS5	COPS5	1	1
MYF5	MYF5	1	1
TLX3	TLX3	1	1
REG1A	REG1A	1	1
HELZ2	HELZ2	1	1
EIF2AK1	EIF2AK1	1	1
GALNT14	GALNT14	1	1
MT1H	MT1H	1	1
RAB2A	RAB2A	1	1
ZMYND12	ZMYND12	1	1
NHLRC4	NHLRC4	1	1
NPEPPS	NPEPPS	1	1
TIA1	TIA1	1	1
ETHE1	ETHE1	1	1
GAS5	GAS5	1	1
FAM174A	FAM174A	1	1
CIDEB	CIDEB	1	1
RTN1	RTN1	1	1
HPS3	HPS3	1	1
METTL25	METTL25	1	1
HCP5B	HCP5B	1	1
HPYR1	HPYR1	1	1
DRG2	DRG2	1	1
TPD52L2	TPD52L2	1	1
MORF4L2-AS:	MORF4L2-AS:	1	1
EPHA6	EPHA6	1	1
LIX1L	LIX1L	1	1
DANCR	DANCR	1	1
SLC35A1	SLC35A1	1	1
YBX1	YBX1	1	1
SIPA1L2	SIPA1L2	1	1
SEC23IP	SEC23IP	1	1
ANKRD19P	ANKRD19P	1	1
KRT31	KRT31	1	1
SNX25	SNX25	1	1
EDARADD	EDARADD	1	1
TXNDC8	TXNDC8	1	1
RIMS2	RIMS2	1	1
U2AF2	U2AF2	1	1
COMMD3	COMMD3	1	1
AGBL5	AGBL5	1	1
ZNF462	ZNF462	1	1
EMC4	EMC4	1	1
GALNT2	GALNT2	1	1

NRTN	NRTN	1	1
CRELD2	CRELD2	1	1
TMEM88	TMEM88	1	1
ERMP1	ERMP1	1	1
ZNF93	ZNF93	1	1
BMP2	BMP2	1	1
FXYD2	FXYD2	1	1
BCL2	BCL2	1	1
ZNF287	ZNF287	1	1
ZFAND4	ZFAND4	1	1
PSTPIP1	PSTPIP1	1	1
AFF3	AFF3	1	1
IRF3	IRF3	1	1
WLS	WLS	1	1
CMTM1	CMTM1	1	1
RNF34	RNF34	1	1
HERC2	HERC2	1	1
NBPF1	NBPF1	1	1
ZCWPW2	ZCWPW2	1	1
KAT2B	KAT2B	1	1
SLFN11	SLFN11	1	1
SNX5	SNX5	1	1
LOC10050647	LOC10050647	1	1
PAG1	PAG1	1	1
WDR37	WDR37	1	1
KLK6	KLK6	1	1
ERCC4	ERCC4	1	1
SAAL1	SAAL1	1	1
CDK8	CDK8	1	1
MS4A5	MS4A5	1	1
P2RX4	P2RX4	1	1
SIPA1L1	SIPA1L1	1	1
N6AMT1	N6AMT1	1	1
CBFB	CBFB	1	1
RSPH3	RSPH3	1	1
HSD17B7P2	HSD17B7P2	1	1
VN1R2	VN1R2	1	1
PIN1P1	PIN1P1	1	1
PUF60	PUF60	1	1
MRPL23	MRPL23	1	1
CASC18	CASC18	1	1
CRK	CRK	1	1
NUP43	NUP43	1	1
EP300	EP300	1	1
GH2	GH2	1	1
MRGPRX1	MRGPRX1	1	1
FAM3A	FAM3A	1	1
USP34	USP34	1	1
MFAP4	MFAP4	1	1
IL17C	IL17C	1	1

RABGGTB	RABGGTB	1	1
FLG-AS1	FLG-AS1	1	1
NBR2	NBR2	1	1
MAPK4	MAPK4	1	1
CHGA	CHGA	1	1
SH3GL1	SH3GL1	1	1
CTSB	CTSB	1	1
BATF	BATF	1	1
PMS2P5	PMS2P5	1	1
EID2B	EID2B	1	1
ZNF18	ZNF18	1	1
PSMB8-AS1	PSMB8-AS1	1	1
ERCC8	ERCC8	1	1
JAK1	JAK1	1	1
HECTD1	HECTD1	1	1
RND1	RND1	1	1
EDF1	EDF1	1	1
CEBPG	CEBPG	1	1
LOC10192838	LOC10192838	1	1
ZNF826P	ZNF826P	1	1
PLA2G1B	PLA2G1B	1	1
FAM185A	FAM185A	1	1
ARG2	ARG2	1	1
C9orf43	C9orf43	1	1
SNHG18	SNHG18	1	1
DTX3	DTX3	1	1
MUTYH	MUTYH	1	1
BNIPL	BNIPL	1	1
POMGNT1	POMGNT1	1	1
SORCS1	SORCS1	1	1
ORM1	ORM1	1	1
DERL3	DERL3	1	1
LANCL1	LANCL1	1	1
OR7E12P	OR7E12P	1	1
CREB3L2	CREB3L2	1	1
PI15	PI15	1	1
PPP1R16A	PPP1R16A	1	1
NOS2	NOS2	1	1
TAF7	TAF7	1	1
BMPR1A	BMPR1A	1	1
PADI4	PADI4	1	1
PPRC1	PPRC1	1	1
MYOZ2	MYOZ2	1	1
LINC00663	LINC00663	1	1
FGFR4	FGFR4	1	1
MOSPD3	MOSPD3	1	1
LINC00308	LINC00308	1	1
POP7	POP7	1	1
IL13	IL13	1	1
CASKIN1	CASKIN1	1	1

ALKBH1	ALKBH1	1	1
LOC10012917	LOC10012917	1	1
RBP5	RBP5	1	1
IL17RE	IL17RE	1	1
CTPS1	CTPS1	1	1
PRADC1	PRADC1	1	1
ARHGAP10	ARHGAP10	1	1
DBR1	DBR1	1	1
PTK6	PTK6	1	1
FXR1	FXR1	1	1
RNASEL	RNASEL	1	1
ACRBP	ACRBP	1	1
NTRK3-AS1	NTRK3-AS1	1	1
ABCC1	ABCC1	1	1
SLC27A2	SLC27A2	1	1
GTF3C4	GTF3C4	1	1
NSDHL	NSDHL	1	1
OPRL1	OPRL1	1	1
ZNF394	ZNF394	1	1
RGL3	RGL3	1	1
OR10J1	OR10J1	1	1
SYNJ1	SYNJ1	1	1
GGT5	GGT5	1	1
GLS	GLS	1	1
LOC1001306[illegible]	LOC1001306[illegible]	1	1
PARP1	PARP1	1	1
SLC22A11	SLC22A11	1	1
PRTG	PRTG	1	1
EPB41L3	EPB41L3	1	1
OR2B6	OR2B6	1	1
CEP76	CEP76	1	1
CSNK2A1	CSNK2A1	1	1
ICE1	ICE1	1	1
SEMA6A	SEMA6A	1	1
FGF10-AS1	FGF10-AS1	1	1
GALNT13	GALNT13	1	1
NPLOC4	NPLOC4	1	1
ZNF669	ZNF669	1	1
ZNF546	ZNF546	1	1
BTNL3	BTNL3	1	1
PAXIP1	PAXIP1	1	1
KLF12	KLF12	1	1
NUP153	NUP153	1	1
UBQLN4	UBQLN4	1	1
PIK3R2	PIK3R2	1	1
FST	FST	1	1
ATG16L2	ATG16L2	1	1
OGDH	OGDH	1	1
PRAMEF11	PRAMEF11	1	1
LINC00313	LINC00313	1	1

SLC45A2	SLC45A2	1	1
SULT1A1	SULT1A1	1	1
PTCH2	PTCH2	1	1
TRRAP	TRRAP	1	1
LHX4-AS1	LHX4-AS1	1	1
ZBTB39	ZBTB39	1	1
SP100	SP100	1	1
NUDT6	NUDT6	1	1
ABCG4	ABCG4	1	1
CCS	CCS	1	1
FAM3B	FAM3B	1	1
SFT2D3	SFT2D3	1	1
OR5L2	OR5L2	1	1
ARMCX5	ARMCX5	1	1
POLR2I	POLR2I	1	1
HPS5	HPS5	1	1
OR1C1	OR1C1	1	1
FOLR3	FOLR3	1	1
SCYL2	SCYL2	1	1
IDE	IDE	1	1
CNGB3	CNGB3	1	1
CYSTM1	CYSTM1	1	1
LRRC28	LRRC28	1	1
C19orf81	C19orf81	1	1
TAS2R38	TAS2R38	1	1
TCF24	TCF24	1	1
CES3	CES3	1	1
THOC5	THOC5	1	1
ERVH-6	ERVH-6	1	1
STK25	STK25	1	1
ADHFE1	ADHFE1	1	1
KIDINS220	KIDINS220	1	1
MOBP	MOBP	1	1
ATP1A1	ATP1A1	1	1
TMEM128	TMEM128	1	1
PDS5A	PDS5A	1	1
SNAI3-AS1	SNAI3-AS1	1	1
PRKD2	PRKD2	1	1
GTF2H2B	GTF2H2B	1	1
CCNB2	CCNB2	1	1
PNPLA4	PNPLA4	1	1
SEC14L1P1	SEC14L1P1	1	1
JKAMP	JKAMP	1	1
ZNF649	ZNF649	1	1
CSE1L	CSE1L	1	1
PRR32	PRR32	1	1
CYP7B1	CYP7B1	1	1
SGSM3	SGSM3	1	1
NAPRT	NAPRT	1	1
OPN1SW	OPN1SW	1	1

SERPINB8	SERPINB8	1	1
FANCD2OS	FANCD2OS	1	1
CCER1	CCER1	1	1
MED22	MED22	1	1
SLC5A8	SLC5A8	1	1
CHD9	CHD9	1	1
VPS25	VPS25	1	1
CYP2B6	CYP2B6	1	1
KRTAP5-AS1	KRTAP5-AS1	1	1
UNC5D	UNC5D	1	1
ZBTB33	ZBTB33	1	1
CD300LF	CD300LF	1	1
ARR3	ARR3	1	1
PEX3	PEX3	1	1
TMEM213	TMEM213	1	1
FAM181A	FAM181A	1	1
GMIP	GMIP	1	1
KLHDC2	KLHDC2	1	1
A2M-AS1	A2M-AS1	1	1
LGSN	LGSN	1	1
GRM6	GRM6	1	1
PATE2	PATE2	1	1
COL6A5	COL6A5	1	1
SH3BGR	SH3BGR	1	1
AKIP1	AKIP1	1	1
OR1E1	OR1E1	1	1
HTRA3	HTRA3	1	1
SPP2	SPP2	1	1
MINPP1	MINPP1	1	1
LINC01000	LINC01000	1	1
IMPA2	IMPA2	1	1
ZNF493	ZNF493	1	1
SAP18	SAP18	1	1
CDK19	CDK19	1	1
ZNF597	ZNF597	1	1
JPH1	JPH1	1	1
LINC00561	LINC00561	1	1
LINC00423	LINC00423	1	1
ADD1	ADD1	1	1
ATXN1L	ATXN1L	1	1
FAM3C	FAM3C	1	1
SND1-IT1	SND1-IT1	1	1
SEC23A	SEC23A	1	1
ABHD2	ABHD2	1	1
KDM3A	KDM3A	1	1
CFL1	CFL1	1	1
COPS7A	COPS7A	1	1
TTC9C	TTC9C	1	1
HMGN4	HMGN4	1	1
NRL	NRL	1	1

TMEM161A	TMEM161A	1	1
TMEM40	TMEM40	1	1
WDR46	WDR46	1	1
STARD3NL	STARD3NL	1	1
ZNF792	ZNF792	1	1
TMEM19	TMEM19	1	1
C19orf53	C19orf53	1	1
CNNM3	CNNM3	1	1
FBXW7	FBXW7	1	1
SYNRG	SYNRG	1	1
AOC2	AOC2	1	1
USP43	USP43	1	1
SHROOM1	SHROOM1	1	1
GLT1D1	GLT1D1	1	1
TPM4	TPM4	1	1
LINC01234	LINC01234	1	1
FGF22	FGF22	1	1
DOCK6	DOCK6	1	1
KIAA1191	KIAA1191	1	1
RSPO1	RSPO1	1	1
CAPRIN2	CAPRIN2	1	1
SCN4B	SCN4B	1	1
PDZD9	PDZD9	1	1
UTF1	UTF1	1	1
RGS3	RGS3	1	1
PRKDC	PRKDC	1	1
MGAT4B	MGAT4B	1	1
SLC22A1	SLC22A1	1	1
JADE3	JADE3	1	1
BIRC6	BIRC6	1	1
SNAPC5	SNAPC5	1	1
NAT16	NAT16	1	1
ATOH8	ATOH8	1	1
CRNKL1	CRNKL1	1	1
ZNF879	ZNF879	1	1
LOC375196	LOC375196	1	1
RDM1	RDM1	1	1
THRAP3	THRAP3	1	1
DUOX1	DUOX1	1	1
ADIRF-AS1	ADIRF-AS1	1	1
FAM216A	FAM216A	1	1
CCDC89	CCDC89	1	1
TKTL1	TKTL1	1	1
AHCTF1	AHCTF1	1	1
ZNF570	ZNF570	1	1
ZNF555	ZNF555	1	1
PGAP3	PGAP3	1	1
IL1RL1	IL1RL1	1	1
RPS16	RPS16	1	1
CCL7	CCL7	1	1

APEX2	APEX2	1	1
LKAAEAR1	LKAAEAR1	1	1
TEK	TEK	1	1
PPARD	PPARD	1	1
ZBTB11-AS1	ZBTB11-AS1	1	1
CST1	CST1	1	1
RNF122	RNF122	1	1
ZSWIM6	ZSWIM6	1	1
S100G	S100G	1	1
FGD3	FGD3	1	1
LOC10192975	LOC10192975	1	1
OR2H1	OR2H1	1	1
LOC729870	LOC729870	1	1
VMA21	VMA21	1	1
FAM223B	FAM223B	1	1
OR1J2	OR1J2	1	1
LSM2	LSM2	1	1
CROT	CROT	1	1
IL37	IL37	1	1
PAQR9	PAQR9	1	1
LZTS3	LZTS3	1	1
VCPIP1	VCPIP1	1	1
CLVS2	CLVS2	1	1
PCAT4	PCAT4	1	1
CALM1	CALM1	1	1
GALNT18	GALNT18	1	1
CENPB	CENPB	1	1
EIF1B	EIF1B	1	1
METTL18	METTL18	1	1
GLUD2	GLUD2	1	1
ACSM2B	ACSM2B	1	1
DPM1	DPM1	1	1
SOX11	SOX11	1	1
SEC61A2	SEC61A2	1	1
NAGK	NAGK	1	1
TTC6	TTC6	1	1
VPS4A	VPS4A	1	1
MRPL24	MRPL24	1	1
LOC284379	LOC284379	1	1
PPP1R15A	PPP1R15A	1	1
PDK3	PDK3	1	1
NDUFS3	NDUFS3	1	1
TMED3	TMED3	1	1
CYLD	CYLD	1	1
CADM3-AS1	CADM3-AS1	1	1
GEMIN5	GEMIN5	1	1
PSG6	PSG6	1	1
PDRG1	PDRG1	1	1
ADAMTSL2	ADAMTSL2	1	1
LOC10192866	LOC10192866	1	1

RBM33	RBM33	1	1
HRH2	HRH2	1	1
ZNF248	ZNF248	1	1
ZNF559	ZNF559	1	1
RGL2	RGL2	1	1
BNIP3L	BNIP3L	1	1
MAGI1	MAGI1	1	1
SCN9A	SCN9A	1	1
LCE2B	LCE2B	1	1
ZNF366	ZNF366	1	1
CDC42EP4	CDC42EP4	1	1
FPR2	FPR2	1	1
LILRB3	LILRB3	1	1
NRGN	NRGN	1	1
KRT16	KRT16	1	1
U2AF1L4	U2AF1L4	1	1
FAM186B	FAM186B	1	1
CHCHD4	CHCHD4	1	1
WWP2	WWP2	1	1
LDHC	LDHC	1	1
UGT3A1	UGT3A1	1	1
CEPT1	CEPT1	1	1
PLRG1	PLRG1	1	1
MED16	MED16	1	1
RAB3C	RAB3C	1	1
MICAL1	MICAL1	1	1
ARHGAP4	ARHGAP4	1	1
GPR3	GPR3	1	1
PAX2	PAX2	1	1
TP53I13	TP53I13	1	1
DOK6	DOK6	1	1
TOMM40L	TOMM40L	1	1
DIP2B	DIP2B	1	1
MITF	MITF	1	1
LINC00908	LINC00908	1	1
ABCF1	ABCF1	1	1
SNORD8	SNORD8	1	1
LINC00996	LINC00996	1	1
ZFPM1	ZFPM1	1	1
LARP1B	LARP1B	1	1
HEATR3	HEATR3	1	1
TNFSF13	TNFSF13	1	1
ARFGEF1	ARFGEF1	1	1
F11	F11	1	1
DEFA5	DEFA5	1	1
CCDC120	CCDC120	1	1
POLD2	POLD2	1	1
ZFP91	ZFP91	1	1
RWDD2A	RWDD2A	1	1
HS6ST1	HS6ST1	1	1

UQCC1	UQCC1	1	1
STARD9	STARD9	1	1
LINC00474	LINC00474	1	1
SUCLG2	SUCLG2	1	1
PET117	PET117	1	1
ESAM	ESAM	1	1
YBX2	YBX2	1	1
RNF14	RNF14	1	1
BTN3A1	BTN3A1	1	1
WDR54	WDR54	1	1
FLAD1	FLAD1	1	1
CDK6	CDK6	1	1
OTUD4	OTUD4	1	1
TP53AIP1	TP53AIP1	1	1
PPA1	PPA1	1	1
LLGL2	LLGL2	1	1
IRAK1BP1	IRAK1BP1	1	1
PRDM13	PRDM13	1	1
MAP3K7	MAP3K7	1	1
TRAPPC6A	TRAPPC6A	1	1
SNX12	SNX12	1	1
EN2	EN2	1	1
TFCP2L1	TFCP2L1	1	1
LAMC2	LAMC2	1	1
KIR2DS4	KIR2DS4	1	1
LGALS3	LGALS3	1	1
SERBP1	SERBP1	1	1
DCBLD1	DCBLD1	1	1
POTEKP	POTEKP	1	1
SCGB1A1	SCGB1A1	1	1
HSF4	HSF4	1	1
TDG	TDG	1	1
POP1	POP1	1	1
VPS54	VPS54	1	1
FUCA1	FUCA1	1	1
HLA-DQB2	HLA-DQB2	1	1
GNE	GNE	1	1
SUN3	SUN3	1	1
RLN3	RLN3	1	1
SLC31A2	SLC31A2	1	1
ATXN2L	ATXN2L	1	1
LINC00857	LINC00857	1	1
LOC10050735	LOC10050735	1	1
PRR14L	PRR14L	1	1
UNC93B1	UNC93B1	1	1
BTBD6	BTBD6	1	1
TAS2R16	TAS2R16	1	1
LINC00970	LINC00970	1	1
C8orf74	C8orf74	1	1
STEAP1B	STEAP1B	1	1

FHL3	FHL3	1	1
PMCHL2	PMCHL2	1	1
PRKCD	PRKCD	1	1
ZNF844	ZNF844	1	1
CIZ1	CIZ1	1	1
KBTBD7	KBTBD7	1	1
FSCN2	FSCN2	1	1
LINC00598	LINC00598	1	1
COPE	COPE	1	1
SIRT3	SIRT3	1	1
CMTM4	CMTM4	1	1
SLC22A2	SLC22A2	1	1
STAC	STAC	1	1
LOC10192708	LOC10192708	1	1
MBTD1	MBTD1	1	1
MCEMP1	MCEMP1	1	1
TM4SF1	TM4SF1	1	1
GRID2	GRID2	1	1
KAZALD1	KAZALD1	1	1
SOX5	SOX5	1	1
CHST13	CHST13	1	1
YIPF2	YIPF2	1	1
BRI3BP	BRI3BP	1	1
FH	FH	1	1
CCSER2	CCSER2	1	1
SCARB2	SCARB2	1	1
FTL	FTL	1	1
NUCB1	NUCB1	1	1
ARHGAP24	ARHGAP24	1	1
FAM32A	FAM32A	1	1
ERLIN2	ERLIN2	1	1
BTAF1	BTAF1	1	1
SNAP29	SNAP29	1	1
WDR7	WDR7	1	1
FIG4	FIG4	1	1
NIP7	NIP7	1	1
FEM1C	FEM1C	1	1
ARC	ARC	1	1
LINC00659	LINC00659	1	1
ZNF671	ZNF671	1	1
MAP4K1	MAP4K1	1	1
KRTAP9-8	KRTAP9-8	1	1
CCDC113	CCDC113	1	1
IFI27L2	IFI27L2	1	1
DDHD2	DDHD2	1	1
LEMD3	LEMD3	1	1
USH2A	USH2A	1	1
TEX41	TEX41	1	1
DNAJB11	DNAJB11	1	1
LOC729683	LOC729683	1	1

LOC10192952	LOC10192952	1	1
RPS29	RPS29	1	1
GCNT1	GCNT1	1	1
NOB1	NOB1	1	1
AP2S1	AP2S1	1	1
LHX3	LHX3	1	1
YPEL2	YPEL2	1	1
CANT1	CANT1	1	1
STXBP1	STXBP1	1	1
ASB14	ASB14	1	1
GPR137C	GPR137C	1	1
ZNF252P	ZNF252P	1	1
ADCY5	ADCY5	1	1
ATAD3C	ATAD3C	1	1
SZRD1	SZRD1	1	1
NDUFB7	NDUFB7	1	1
LCMT2	LCMT2	1	1
CALHM2	CALHM2	1	1
KRBA2	KRBA2	1	1
GAD2	GAD2	1	1
ADAMTS14	ADAMTS14	1	1
CD33	CD33	1	1
RCVRN	RCVRN	1	1
NR0B1	NR0B1	1	1
KCNB1	KCNB1	1	1
HINT2	HINT2	1	1
LEPROTL1	LEPROTL1	1	1
LRP8	LRP8	1	1
TTC4	TTC4	1	1
ASAH1	ASAH1	1	1
RPH3A	RPH3A	1	1
LINC00115	LINC00115	1	1
CDKN2AIP	CDKN2AIP	1	1
GPATCH3	GPATCH3	1	1
CACNG5	CACNG5	1	1
BDNF	BDNF	1	1
TARP	TARP	1	1
SHISA5	SHISA5	1	1
ZFC3H1	ZFC3H1	1	1
UBXN7-AS1	UBXN7-AS1	1	1
BLM	BLM	1	1
ZNF655	ZNF655	1	1
RBPJL	RBPJL	1	1
SPATA5L1	SPATA5L1	1	1
EFCAB5	EFCAB5	1	1
RHOB	RHOB	1	1
LZTFL1	LZTFL1	1	1
IL15RA	IL15RA	1	1
STYK1	STYK1	1	1
DFFB	DFFB	1	1

CSMD1	CSMD1	1	1
TTLL1	TTLL1	1	1
FAM8A1	FAM8A1	1	1
ITGA2B	ITGA2B	1	1
TAOK1	TAOK1	1	1
JAKMIP2	JAKMIP2	1	1
ZSCAN21	ZSCAN21	1	1
KRT36	KRT36	1	1
CTSF	CTSF	1	1
AASDHPPT	AASDHPPT	1	1
LRRC41	LRRC41	1	1
MMS19	MMS19	1	1
SBK1	SBK1	1	1
CENPE	CENPE	1	1
DLG1-AS1	DLG1-AS1	1	1
ELF4	ELF4	1	1
MAN2A2	MAN2A2	1	1
RBM5	RBM5	1	1
IL1RAPL2	IL1RAPL2	1	1
SERPINI2	SERPINI2	1	1
KIAA1549	KIAA1549	1	1
CTSO	CTSO	1	1
TNFSF9	TNFSF9	1	1
NFKBIL1	NFKBIL1	1	1
SMG6	SMG6	1	1
TIMM44	TIMM44	1	1
TUBGCP3	TUBGCP3	1	1
SSX2IP	SSX2IP	1	1
METTL14	METTL14	1	1
NME5	NME5	1	1
MYH7B	MYH7B	1	1
ZBTB26	ZBTB26	1	1
GNRHR	GNRHR	1	1
SHC3	SHC3	1	1
LDHAL6A	LDHAL6A	1	1
ANO6	ANO6	1	1
TTI1	TTI1	1	1
SYCP1	SYCP1	1	1
AR	AR	1	1
ZMYND11	ZMYND11	1	1
PTBP2	PTBP2	1	1
MAPRE1	MAPRE1	1	1
FBRSL1	FBRSL1	1	1
IL1RAP	IL1RAP	1	1
PGR	PGR	1	1
BIK	BIK	1	1
SAFB	SAFB	1	1
RUNX1-IT1	RUNX1-IT1	1	1
SIK3	SIK3	1	1
NAA50	NAA50	1	1

RBP3	RBP3	1	1
FSD1	FSD1	1	1
LOC1019294€	LOC1019294€	1	1
ZNF432	ZNF432	1	1
GSPT1	GSPT1	1	1
ZNF449	ZNF449	1	1
ZNF829	ZNF829	1	1
LOC1019289ε	LOC1019289ε	1	1
TAF1A-AS1	TAF1A-AS1	1	1
RAD21L1	RAD21L1	1	1
APOBEC3D	APOBEC3D	1	1
GCC1	GCC1	1	1
FLT3LG	FLT3LG	1	1
NDUFA4L2	NDUFA4L2	1	1
IL1RL2	IL1RL2	1	1
ASMTL	ASMTL	1	1
RPL35	RPL35	1	1
LINC01169	LINC01169	1	1
IKBKG	IKBKG	1	1
SLC12A9	SLC12A9	1	1
KLHL34	KLHL34	1	1
RIPK3	RIPK3	1	1
ARMC9	ARMC9	1	1
PIWIL4	PIWIL4	1	1
OCLN	OCLN	1	1
STK39	STK39	1	1
ABCB8	ABCB8	1	1
CPT1C	CPT1C	1	1
IGF2BP3	IGF2BP3	1	1
MOB3A	MOB3A	1	1
HPCAL1	HPCAL1	1	1
PPM1B	PPM1B	1	1
B3GALNT2	B3GALNT2	1	1
SPHK1	SPHK1	1	1
ZNF460	ZNF460	1	1
DENR	DENR	1	1
TTPAL	TTPAL	1	1
TREML5P	TREML5P	1	1
FSCN3	FSCN3	1	1
PAIP2	PAIP2	1	1
PLEKHA8P1	PLEKHA8P1	1	1
PLD4	PLD4	1	1
MOGAT1	MOGAT1	1	1
RPL13AP17	RPL13AP17	1	1
PRDM6	PRDM6	1	1
NAP1L5	NAP1L5	1	1
HCN1	HCN1	1	1
MAGEA10	MAGEA10	1	1
S100A7	S100A7	1	1
MS4A3	MS4A3	1	1

BCL10	BCL10	1	1
TBR1	TBR1	1	1
HMGA1	HMGA1	1	1
FIZ1	FIZ1	1	1
SLC7A3	SLC7A3	1	1
ZNF704	ZNF704	1	1
TMEM120A	TMEM120A	1	1
IRGQ	IRGQ	1	1
FGF4	FGF4	1	1
LOC10050584	LOC10050584	1	1
UBQLN3	UBQLN3	1	1
SPRR2C	SPRR2C	1	1
LOC10050623	LOC10050623	1	1
SLC6A12	SLC6A12	1	1
TTC13	TTC13	1	1
GRK7	GRK7	1	1
ERVFRD-1	ERVFRD-1	1	1
TEDDM1	TEDDM1	1	1
TMX3	TMX3	1	1
HNRNPM	HNRNPM	1	1
OAF	OAF	1	1
ZNF100	ZNF100	1	1
GPR25	GPR25	1	1
ITGAL	ITGAL	1	1
PLXNA1	PLXNA1	1	1
CC2D2A	CC2D2A	1	1
FGF13-AS1	FGF13-AS1	1	1
AFF4	AFF4	1	1
CDK12	CDK12	1	1
LOC10272403	LOC10272403	1	1
ZSCAN10	ZSCAN10	1	1
SMARCA5	SMARCA5	1	1
KHNYN	KHNYN	1	1
MTCP1	MTCP1	1	1
BZW1	BZW1	1	1
LCMT1	LCMT1	1	1
CXCR6	CXCR6	1	1
MYL5	MYL5	1	1
S100Z	S100Z	1	1
DLL3	DLL3	1	1
MOB1A	MOB1A	1	1
LINC00052	LINC00052	1	1
ZNF652	ZNF652	1	1
HPRT1	HPRT1	1	1
FGF10	FGF10	1	1
ADAMTS7	ADAMTS7	1	1
SNRPC	SNRPC	1	1
ZHX3	ZHX3	1	1
PRRC1	PRRC1	1	1
C20orf181	C20orf181	1	1
