# Supplementary material for: Myeloid-specific S100A8/A9 deficiency attenuates atrial fibrillation through prevention of TLR4/NF-kB-mediated immune cell recruitment and inflammation
Source: Front Immunol. 2025 Sep 4;16:1623486. doi: 10.3389/fimmu.2025.1623486 (PMC12443547; doi:10.3389/fimmu.2025.1623486)
Supplement: Supplementary file 11 [file DataSheet6.pdf]

row.name:	ID	Description	GeneRatio	BgRatio	pvalue
hsa04657	hsa04657	IL-17 signaling pathway	3/12	94/8586	0.00026
hsa04145	hsa04145	Phagosome	2/12	152/8586	0.018292
hsa04921	hsa04921	Oxytocin signaling pathway	2/12	154/8586	0.018749
hsa05167	hsa05167	Kaposi sarcoma-associated herpesvirus infectio	2/12	194/8586	0.028884
hsa04020	hsa04020	Calcium signaling pathway	2/12	253/8586	0.046987
hsa04962	hsa04962	Vasopressin-regulated water reabsorption	1/12	44/8586	0.059829
hsa05144	hsa05144	Malaria	1/12	50/8586	0.067728
hsa05134	hsa05134	Legionellosis	1/12	56/8586	0.075566
hsa04978	hsa04978	Mineral absorption	1/12	60/8586	0.080758
hsa04929	hsa04929	GnRH secretion	1/12	64/8586	0.085923
hsa00830	hsa00830	Retinol metabolism	1/12	68/8586	0.091062
hsa05120	hsa05120	Epithelial cell signaling in Helicobacter pylori in	1/12	70/8586	0.093621
hsa05140	hsa05140	Leishmaniasis	1/12	77/8586	0.102527
hsa04260	hsa04260	Cardiac muscle contraction	1/12	87/8586	0.11511
hsa04512	hsa04512	ECM-receptor interaction	1/12	89/8586	0.117607
hsa04976	hsa04976	Bile secretion	1/12	89/8586	0.117607
hsa05032	hsa05032	Morphine addiction	1/12	91/8586	0.120098
hsa05323	hsa05323	Rheumatoid arthritis	1/12	93/8586	0.122583
hsa05150	hsa05150	Staphylococcus aureus infection	1/12	96/8586	0.126297
hsa04666	hsa04666	Fc gamma R-mediated phagocytosis	1/12	97/8586	0.127532
hsa04713	hsa04713	Circadian entrainment	1/12	97/8586	0.127532
hsa04061	hsa04061	Viral protein interaction with cytokine and cyto	1/12	100/8586	0.131227
hsa05146	hsa05146	Amoebiasis	1/12	102/8586	0.133682
hsa04064	hsa04064	NF-kappa B signaling pathway	1/12	104/8586	0.136132
hsa04725	hsa04725	Cholinergic synapse	1/12	113/8586	0.147074
hsa04668	hsa04668	TNF signaling pathway	1/12	114/8586	0.148282
hsa04724	hsa04724	Glutamatergic synapse	1/12	115/8586	0.149488
hsa04726	hsa04726	Serotonergic synapse	1/12	115/8586	0.149488
hsa04919	hsa04919	Thyroid hormone signaling pathway	1/12	121/8586	0.156694
hsa04650	hsa04650	Natural killer cell mediated cytotoxicity	1/12	132/8586	0.169759
hsa04728	hsa04728	Dopaminergic synapse	1/12	132/8586	0.169759
hsa04380	hsa04380	Osteoclast differentiation	1/12	135/8586	0.17329
hsa04915	hsa04915	Estrogen signaling pathway	1/12	137/8586	0.175636
hsa05322	hsa05322	Systemic lupus erythematosus	1/12	137/8586	0.175636
hsa04936	hsa04936	Alcoholic liver disease	1/12	142/8586	0.181475
hsa04723	hsa04723	Retrograde endocannabinoid signaling	1/12	148/8586	0.188432
hsa05152	hsa05152	Tuberculosis	1/12	180/8586	0.224627
hsa04621	hsa04621	NOD-like receptor signaling pathway	1/12	186/8586	0.231247
hsa04613	hsa04613	Neutrophil extracellular trap formation	1/12	191/8586	0.236724
hsa04062	hsa04062	Chemokine signaling pathway	1/12	192/8586	0.237815
hsa04510	hsa04510	Focal adhesion	1/12	203/8586	0.249722
hsa05417	hsa05417	Lipid and atherosclerosis	1/12	215/8586	0.262518
hsa05208	hsa05208	Chemical carcinogenesis - reactive oxygen spec	1/12	223/8586	0.270936
hsa04060	hsa04060	Cytokine-cytokine receptor interaction	1/12	297/8586	0.344737
hsa05165	hsa05165	Human papillomavirus infection	1/12	331/8586	0.376293
hsa04151	hsa04151	PI3K-Akt signaling pathway	1/12	359/8586	0.401228

p.adjust	qvalue	geneID	Count
0.011973	0.011233	6279/2919	3
0.237625	0.222944	1311/2215	2
0.237625	0.222944	1827/3760	2
0.237625	0.222944	1827/2919	2
0.237625	0.222944	10345/552	2
0.237625	0.222944	361	1
0.237625	0.222944	1311	1
0.237625	0.222944	2919	1
0.237625	0.222944	115019	1
0.237625	0.222944	3760	1
0.237625	0.222944	10170	1
0.237625	0.222944	2919	1
0.237625	0.222944	2215	1
0.237625	0.222944	10345	1
0.237625	0.222944	1311	1
0.237625	0.222944	361	1
0.237625	0.222944	3760	1
0.237625	0.222944	2919	1
0.237625	0.222944	2215	1
0.237625	0.222944	2215	1
0.237625	0.222944	3760	1
0.237625	0.222944	2919	1
0.237625	0.222944	2919	1
0.237625	0.222944	2919	1
0.237625	0.222944	3760	1
0.237625	0.222944	2919	1
0.237625	0.222944	3760	1
0.237625	0.222944	3760	1
0.237625	0.222944	1827	1
0.237625	0.222944	2215	1
0.237625	0.222944	3760	1
0.237625	0.222944	2215	1
0.237625	0.222944	3760	1
0.237625	0.222944	2215	1
0.23851	0.223774	2919	1
0.240774	0.225898	3760	1
0.273487	0.25659	2215	1
0.273487	0.25659	2919	1
0.273487	0.25659	2215	1
0.273487	0.25659	2919	1
0.280176	0.262866	1311	1
0.287519	0.269755	2919	1
0.289839	0.271931	115019	1
0.360407	0.33814	2919	1
0.384655	0.36089	1311	1
0.401228	0.376438	1311	1
