# Supplementary material for: Myeloid-specific S100A8/A9 deficiency attenuates atrial fibrillation through prevention of TLR4/NF-kB-mediated immune cell recruitment and inflammation
Source: Front Immunol. 2025 Sep 4;16:1623486. doi: 10.3389/fimmu.2025.1623486 (PMC12443547; doi:10.3389/fimmu.2025.1623486)

Full and uncropped western blot for Figure 5B

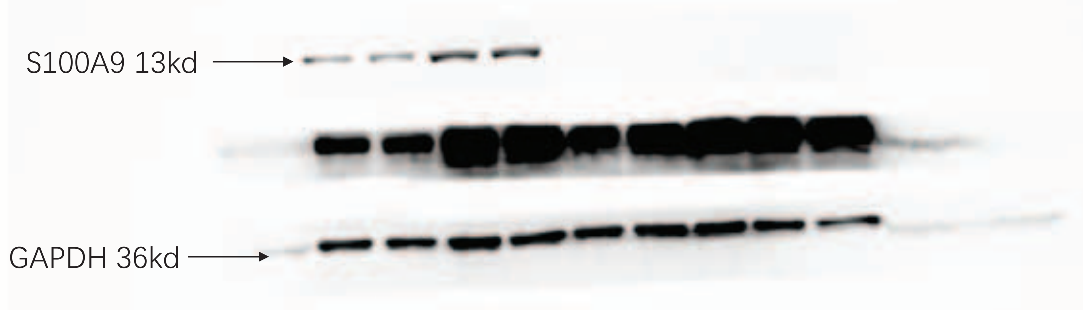

Full and uncropped western blot for Figure 5B

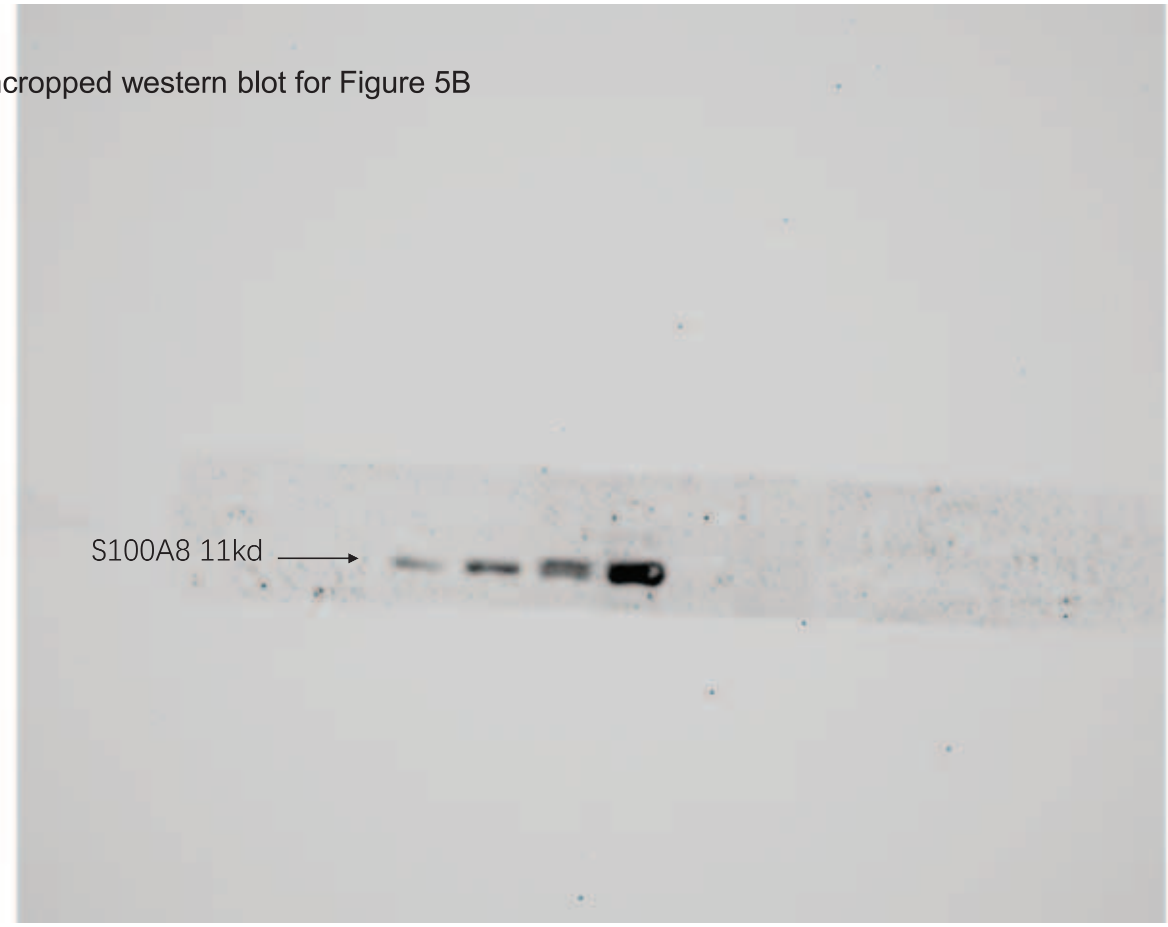

Full and uncropped western blot for Figure 6C

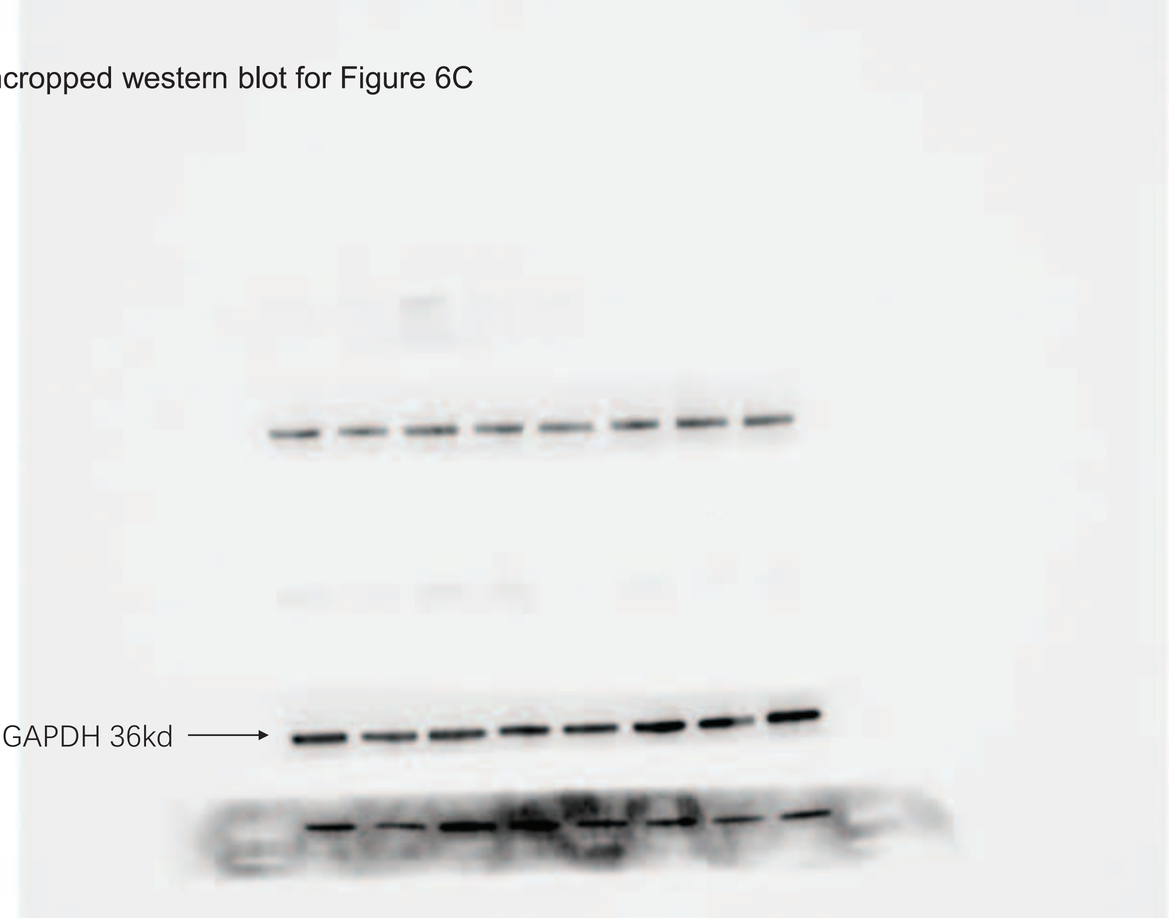

Full and uncropped western blot for Figure 6C

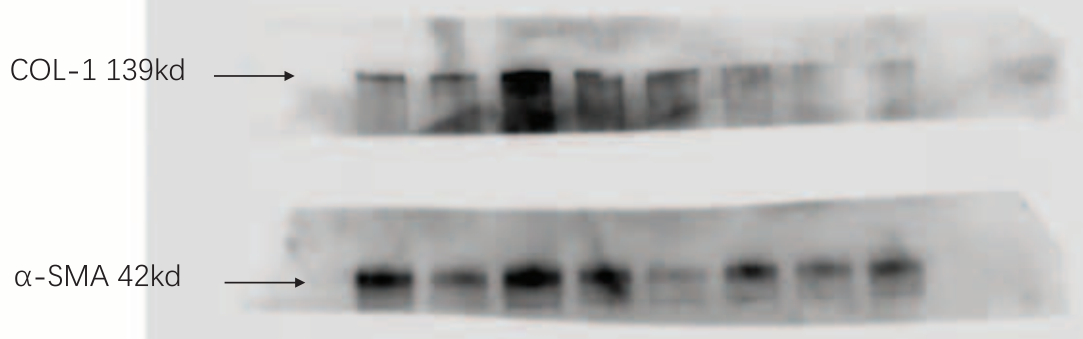

Full and uncropped western blot for Figure 7H

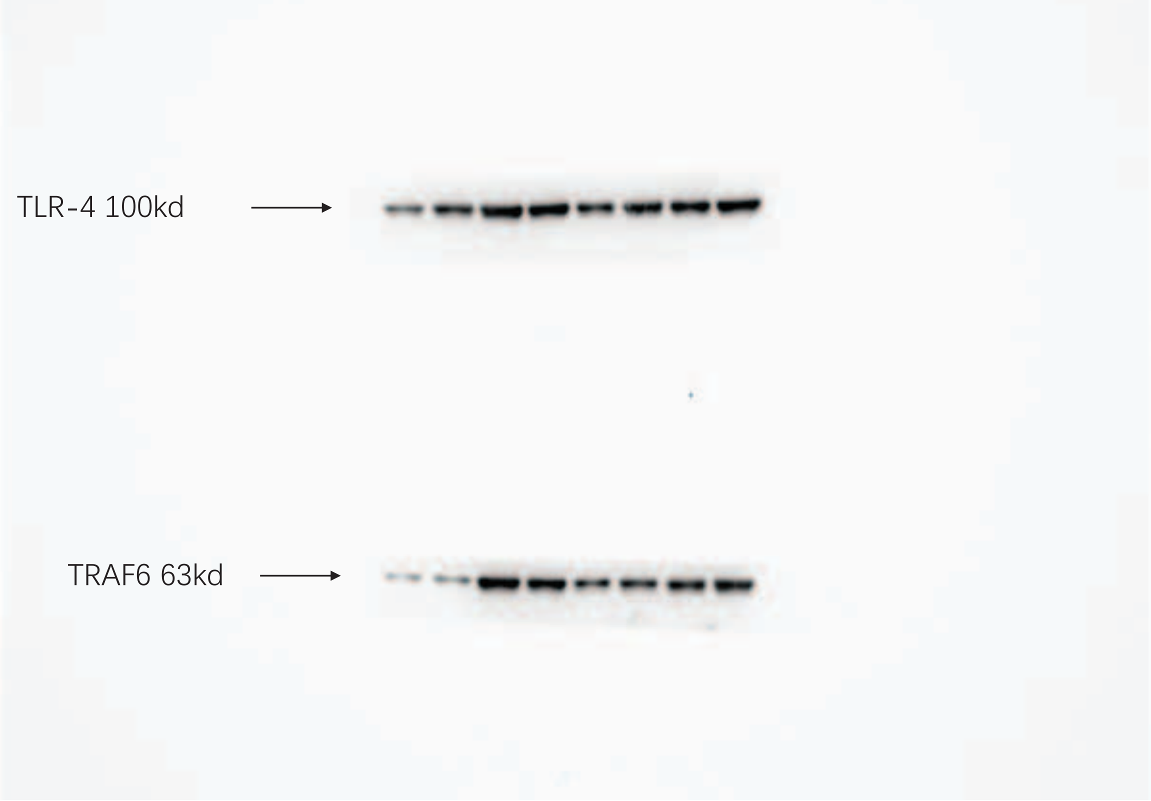

Full and uncropped western blot for Figure 7H

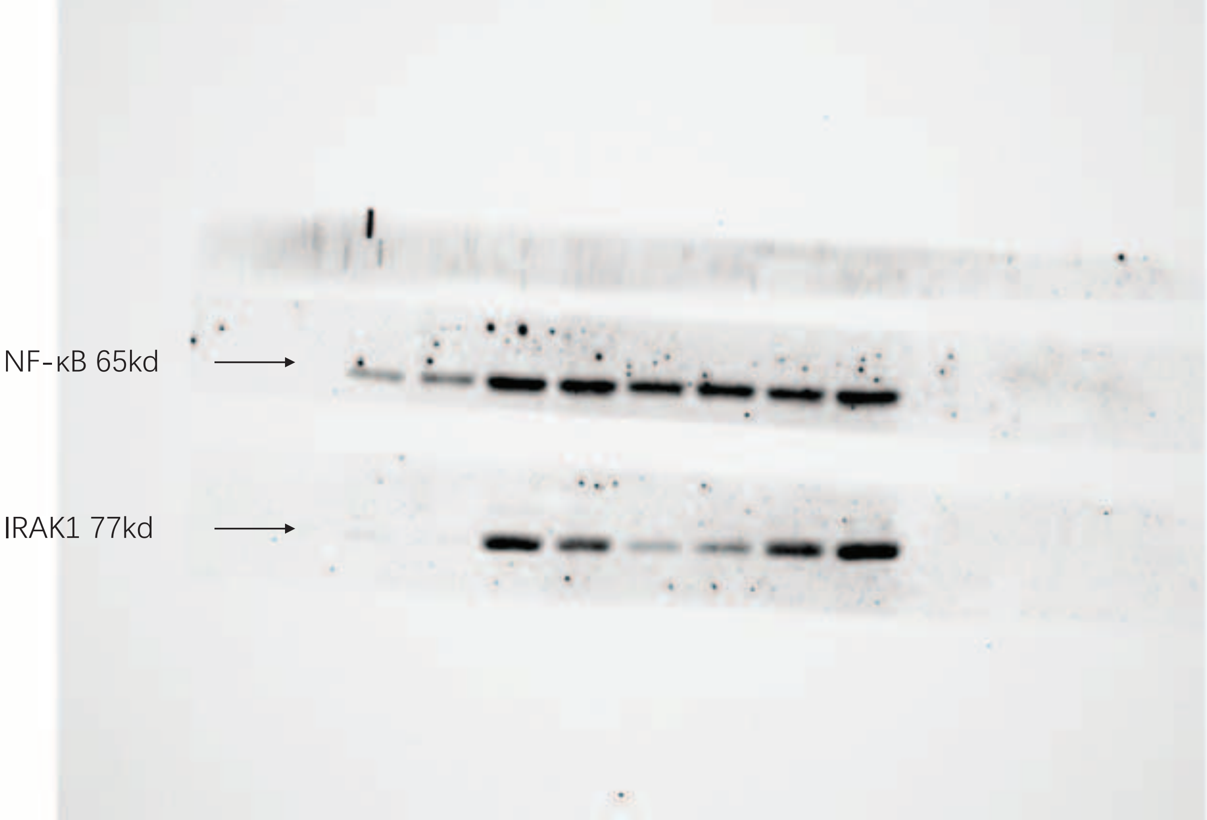

Full and uncropped western blot for Figure 7H

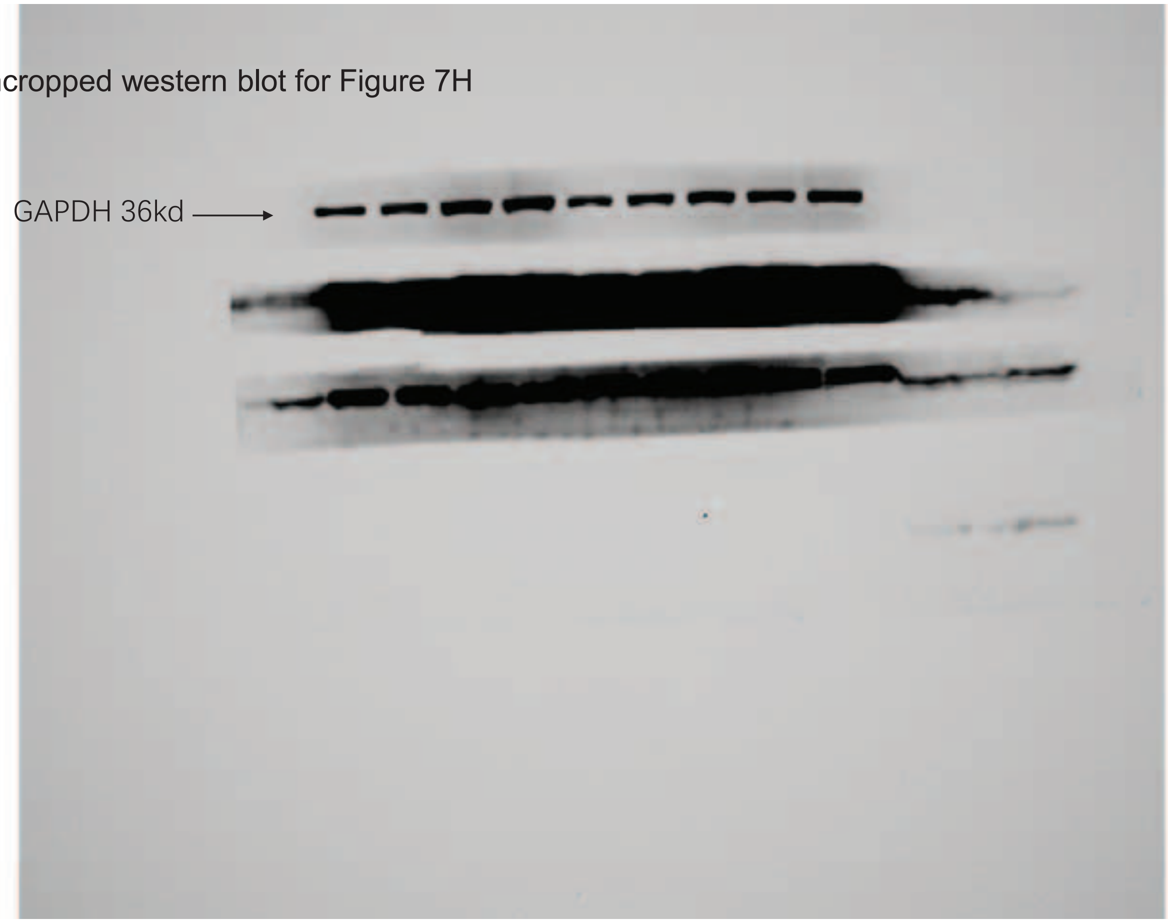

Supplement: Supplementary file 12 [file DataSheet7.pdf]
